# Single‐cell analysis reveals neuroprotective histone deacetylase inhibitor pathways

**DOI:** 10.1002/alz.71108

**Published:** 2026-02-03

**Authors:** Madeline Peyton, Nur Jury‐Garfe, Jiahui Liu, Caleb Beimfohr, Chitra Sunil, Steven Brooks, Pengyue Zhang, Sean D. McCabe, Timothy I. Richardson, Kun Huang, Cristian A. Lasagna‐Reeves, Jie Zhang, Travis S. Johnson

**Affiliations:** ^1^ Department of Biostatistics and Health Data Science Indiana University Indianapolis Indianapolis Indiana USA; ^2^ Center for Biomedical Informatics Regenstrief Institute Indianapolis Indiana USA; ^3^ Indiana Biosciences Research Institute Indianapolis Indiana USA; ^4^ Department of Anatomy Cell Biology & Physiology, Indiana University Indianapolis Indianapolis Indiana USA; ^5^ Department of Neurology Baylor College of Medicine Houston Texas USA; ^6^ Department of Medicine Division of Clinical Pharmacology School of Medicine Indiana University Indianapolis Indianapolis Indiana USA; ^7^ Department of Environmental & Public Health Sciences University of Cincinnati Cincinnati Ohio USA; ^8^ Indiana University Melvin and Bren Simon Comprehensive Cancer Center Indianapolis Indiana USA; ^9^ Department of Medical and Molecular Genetics Indiana University Indianapolis Indianapolis Indiana USA

**Keywords:** AD, Alzheimer's disease, amyloid‐beta, DISC1, drug repurposing, HDAC, histone deacetylase inhibitor, microglia, neuroinflammation, neuroprotection, scRNA‐seq, single‐cell RNA sequencing, trichostatin‐A, TSA

## Abstract

**INTRODUCTION:**

Alzheimer's disease (AD) involves β‐amyloid (Aβ) accumulation, tau pathology, and neuroinflammation, driving cognitive decline. Despite extensive research, disease‐modifying therapies remain elusive. We integrated single‐cell RNA sequencing (scRNA‐seq), spatial transcriptomics, and in vitro validation to identify repurposable drugs for AD^1^.

**METHODS:**

Computational drug repurposing was performed using cell‐type‐specific analysis of scRNA‐seq datasets from AD cortical regions. Trichostatin‐A (TSA) effects were validated in human induced pluripotent stem cells (iPSC) ‐derived cortical neurons exposed to Aβ oligomers. Cross‐dataset integration identified convergent therapeutic targets.

**RESULTS:**

TSA emerged as the top candidate, protecting neurons from Aβ toxicity and preserving synaptic integrity. *DISC1* (Disrupted‐In‐Schizophrenia 1) was uniquely upregulated across TSA‐treated neurons, AD‐associated neuronal subpopulations, and protective microglial subtypes.

**DISCUSSION:**

*DISC1* represents a convergent therapeutic target for AD, mediating TSA's neuroprotective effects through pathways regulating *GSK3β*, mitochondrial transport, and synaptic plasticity, providing a mechanistic framework for developing AD therapeutics.

## Highlights

- Single‐cell analysis identified trichostatin‐A as the top drug candidate for Alzheimer's disease (AD).
- DISC1 emerged as the only gene upregulated across all experimental conditions.
- Trichostatin‐A (TSA) protected human neurons from amyloid‐β toxicity and preserved synapses.
- DISC1 upregulation by TSA persisted even in the presence of amyloid‐β pathology.
- Novel multi‐modal framework integrates computational and experimental validation.

## 1 BACKGROUND

Alzheimer's disease (AD) is a progressive neurodegenerative disease affecting an estimated six million Americans aged 65 and older.^1^ The National Institute on Aging (NIA) ranks AD as the third leading cause of death for older adults in the United States,^2^ with the Centers for Disease Control and Prevention (CDC) estimating cases will nearly triple to 14 million by 2060.^3^

Despite extensive research, the etiology of AD remains unclear. Key hallmarks include amyloid plaques, neurofibrillary tangles, chronic inflammation, loss of neuronal connections, and cell death.^4^ AD brains have abnormal levels of β‐amyloid (Aβ) peptides that oligomerize and aggregate, forming plaques that disrupt neuronal function.^4^ Neurofibrillary tangles from tau protein accumulation block synaptic communication.^4^ Both tau and Aβ pathology are tied to chronic inflammation that is associated with glial cells.^4^ Microglia fail to remove Aβ plaques as the disease progresses,^5^ resulting in neuronal death, synaptic breakdown, and brain atrophy.

As genomic data accumulate, opportunities to identify novel drug targets increase. However, drug development is costly due to high attrition rates, with only 12% of new drugs entering clinical trials receiving United States Food and Drug Administration (FDA) approval.^6^ Drug repurposing identifies new indications for previously approved or investigational new molecular entities (NMEs) beyond their original scope.^7^ This allows shorter development timeframes, is more cost‐effective, and uses NMEs already proven safe in phase 1 trials.^7^

Several computational drug repurposing efforts for AD have shown degrees of success. Network‐based approaches have identified FDA‐approved candidates, including antihypertensives, anti‐inflammatory agents, and lipid‐lowering drugs.^8^ Clinical trials have explored diabetes medications, antimicrobials, and cardiovascular drugs.^9^, ^10^ However, many promising candidates have failed clinically, highlighting critical pipeline gaps.^11^

A major limitation has been reliance on bulk tissue transcriptomics, which averages gene expression across heterogeneous cell populations and obscures cell type‐specific disease mechanisms and drug responses. Platforms like Drug Repurposing in AD (DRIAD), using bulk RNA sequencing (RNA‐seq) data,^12^ cannot distinguish whether drugs act on neurons, glia, or vascular cells, which is critical given AD's multicellular pathology. Most predictions also lack spatial context and validation in human‐relevant systems before advancing to clinical trials.

To address these gaps, we developed a multi‐modal framework integrating single‐cell RNA sequencing (scRNA‐seq), spatial transcriptomics (ST), and human induced pluripotent stem cells (iPSC) ‐derived neuronal validation. scRNA‐seq resolves gene expression at individual cell resolution, enabling identification of cell type‐specific disease mechanisms and drug responses. This is crucial for AD, where distinct cell types contribute differentially to disease pathogenesis. ST preserves tissue architecture while measuring gene expression, determining which anatomical regions would be affected by candidate drugs. Finally, we validated computational predictions in human iPSC‐derived cortical neurons exposed to amyloid‐beta oligomers, testing whether predicted drugs protect human neurons and identifying underlying molecular mechanisms.

We applied this framework using scRNA‐seq data from Grubman et al,^13^ Mathys et al.^14^ and Green et al.^15^ for cell type‐specific drug repurposing, identifying drugs reversing disease‐associated gene expression in specific neuronal and glial populations.

**RESEARCH IN CONTEXT**

1. **Systematic review**: We reviewed computational drug repurposing studies for Alzheimer's disease (AD) using PubMed, Google Scholar, and recent conference abstracts. While machine learning approaches have been applied to bulk RNA‐seq data, few studies have leveraged single‐cell resolution to identify cell type‐specific therapeutic targets. Trichostatin‐A's (TSA's) neuroprotective mechanisms and DISC1's role as a potential mediator remain unexplored in AD contexts.
2. **Interpretation**: Our integrated analysis of single‐cell RNA sequencing (scRNA‐seq), spatial transcriptomics (ST), and functional validation identifies TSA as a promising AD therapeutic with DISC1 as a key convergent mediator. DISC1's consistent upregulation across TSA‐treated neurons, AD‐associated populations, and protective microglial subtypes provides novel mechanistic insights into the epigenetics of neurodegeneration.
3. **Future directions**: Critical next steps include validating DISC1's role in human neuronal models, developing DISC1‐selective modulators, testing efficacy in brain organoids and transgenic mice, optimizing TSA pharmacokinetics for brain penetration, and identifying DISC1 downstream effectors as therapeutic targets or diagnostics.

Using LINCS L1000 signatures,^16^ we identified trichostatin‐A (TSA) as a top candidate. Validation in iPSC‐derived cortical neurons confirmed TSA's protection against Aβ‐induced neurotoxicity and preservation of synaptic integrity. ST localized TSA's impacts in the hippocampus and cerebral cortex. We performed Diagnostic Evidence Gauge of Single cells (DEGAS) analysis^17^ to identify AD‐associated neuronal subpopulations and analyzed TSA‐induced transcriptional changes. We characterized microglial heterogeneity using datasets from Lee et al.^18^ and integrated findings across multiple datasets.

Comprehensive transcriptomic analysis of iPSC‐derived cortical neurons treated with TSA and β‐amyloid revealed that TSA significantly upregulates *DISC1* (Disrupted‐In‐Schizophrenia 1), critical for neurodevelopment and synaptic function. These transcriptional effects persist with Aβ presence, suggesting mechanisms counteracting AD pathology. *DISC1* regulates axon guidance, synapse formation, neuronal migration, and mitochondrial trafficking,^19^ making it a promising AD target.

These findings underscore the utility of integrating scRNA‐seq, ST, and in vitro validation to accelerate drug repurposing for AD, with TSA as a promising therapeutic candidate.

## 2 METHODS

### 2.1 Experimental approach and workflow

**FIGURE 1:**
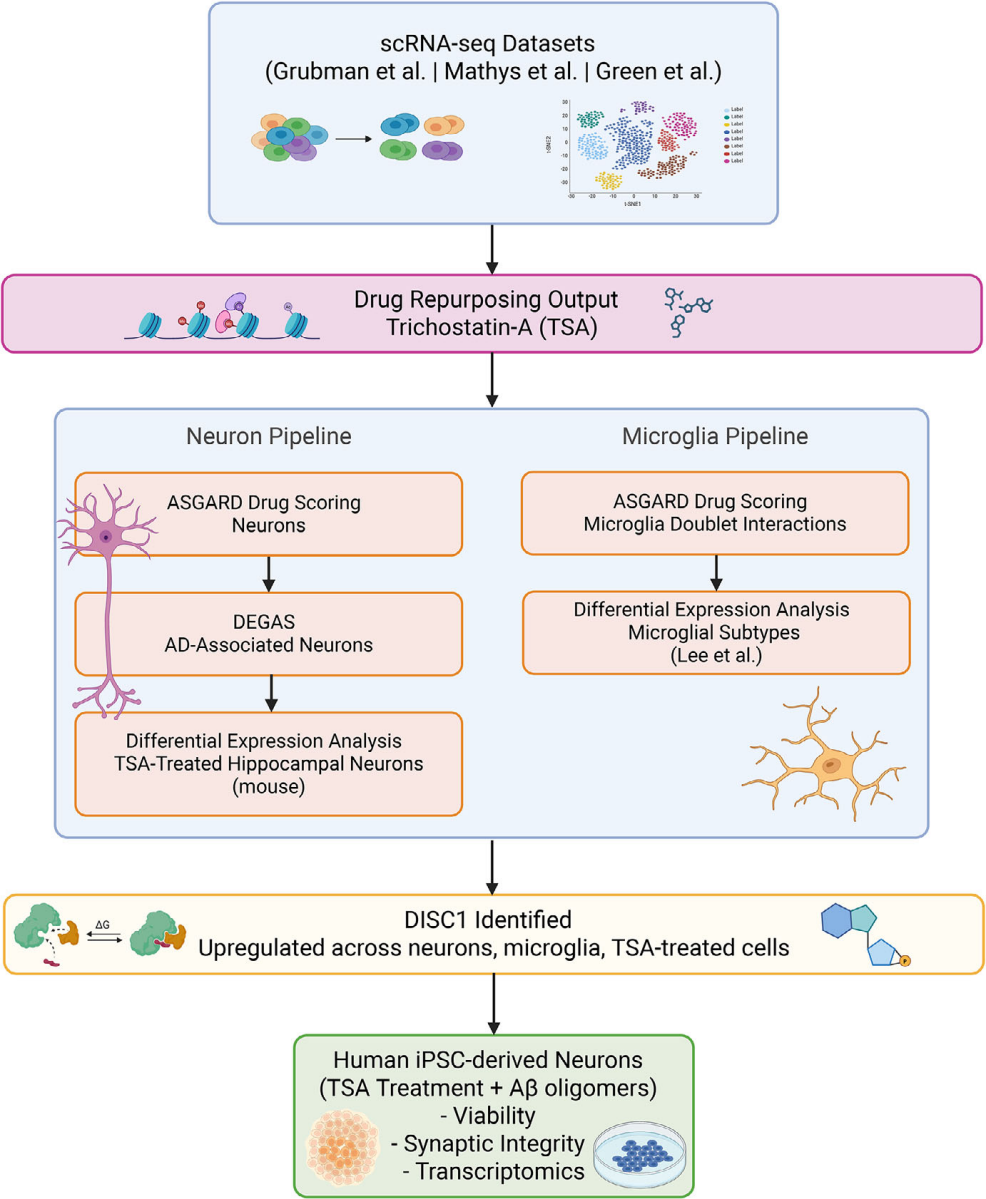
Integrative multi‐cell type analysis workflow identifies DISC1 as a convergent target of TSA in AD. Schematic representation of the multi‐pronged analytical framework used to identify and validate TSA as a therapeutic candidate for AD. The workflow began with cell‐type‐specific drug repurposing analysis of scRNA‐seq data from Grubman et al.,^13^ Mathys et al.,^14^ and Green et al.^15^ datasets, resulting in the identification of TSA as a promising compound. We strategically investigated TSA's effects through two parallel cell type‐specific pathways: neurons and microglia. These cell types were specifically selected based on their known vulnerability in AD pathology and significant drug scores in our initial ASGARD analysis. For neurons, we applied DEGAS analysis to identify AD‐associated neuronal subpopulations, followed by differential expression analysis of TSA‐treated neurons in mice. Concurrently, we examined microglia, which showed high TSA drug scores in doublet interactions, and conducted differential expression analysis across microglial subtypes. Both analytical branches converged on DISC1 as a key upregulated gene, which was subsequently validated in human iPSC‐derived neuronal models through both phenotypic assays and transcriptomic analysis. This cell type‐specific approach enabled the identification of DISC1 as a potential mechanistic target underlying TSA's neuroprotective effect in AD. Created in BioRender. Peyton, M. (2025) https://biorender.com/73irecf. AD, Alzheimer's disease; ASGARD, A Single Cell Guided Pipeline to Aid Repurposing of Drugs; DEGAS, Diagnostic Evidence Gauge of Single cells; DISC1, Disrupted‐In‐Schizophrenia 1; iPSC, induced pluripotent stem cell; scRNA‐seq, single‐cell RNA sequencing; TSA, trichostatin‐A.

To identify and validate potential drug repurposing candidates for the treatment of AD, we developed a comprehensive three‐phase analytical framework integrating computational discovery, in silico mechanistic investigation, and experimental validation (Figure 1).

Our workflow began with systematic drug repurposing across multiple independent single‐cell RNA‐sequencing datasets to identify convergent therapeutic candidates. We collected and integrated scRNA‐seq data from four major sources: Grubman et al.^13^ (entorhinal cortex), Mathys et al.^14^ (prefrontal cortex), Green et al.^15^ (aged prefrontal cortex), and Lee et al.^18^ (microglial subtypes). These datasets underwent rigorous quality control, normalization, and cell type annotation procedures before drug screening using the A Single Cell Guided Pipeline to Aid Repurposing of Drugs (ASGARD) platform. ASGARD enabled cell type‐specific drug scoring to identify candidate compounds with potential therapeutic relevance in AD.

To understand the molecular mechanisms underlying top drug candidates, we employed multiple computational approaches. We applied the DEGAS framework to stratify neuronal populations and identify cellular subpopulations with greater AD associations. Single‐cell characterization of myeloid cells across two independent datasets enabled detailed transcriptional profiling of AD‐associated microglial subtypes. We performed differential expression analysis of TSA‐treated hippocampal neurons to identify transcriptional responses and conducted cross‐species ortholog mapping to assess conservation of drug effects between mouse and human. Integrative multi‐dataset analysis identified consistently dysregulated genes and pathways across cohorts. Finally, we analyzed spatially resolved transcriptional signatures using 10X Genomics Visium ST technology to map brain region‐specific enrichment of drug‐responsive gene signatures.

Top candidates identified through computational methods underwent validation in human iPSC‐derived cortical neuronal models. We assessed drug effects on cell viability and synaptic integrity in the presence of Aβ pathology. Transcriptomic profiling through RNA‐seq characterized genome‐wide transcriptional responses to drug treatment, both alone and in combination with Aβ exposure. Immunofluorescence microscopy provided orthogonal validation of key molecular targets and cellular phenotypes. This integrated three‐phase approach enabled rigorous identification and mechanistic characterization of drug candidates with translational potential.

### 2.2 Single‐cell transcriptomics data

We integrated four independent single‐cell RNA‐sequencing datasets encompassing diverse brain regions, disease stages, and cellular populations. Single‐nucleus RNA‐seq data from entorhinal cortex tissue (GEO accession GSE138852)^13^ included AD patients (*n* = 6) and age‐matched controls (*n* = 6). This dataset provided cell‐type‐specific transcriptional profiles from a brain region with early vulnerability in AD pathogenesis. Single‐nucleus RNA‐seq data from prefrontal cortex tissue (Synapse ID: syn18485175)^14^ included 48 individuals spanning a range of AD pathology. This dataset enabled examination of drug candidates in a brain region affected later in AD progression. Single‐nucleus RNA‐seq data from aged prefrontal cortex samples (Synapse ID: syn31512863)^15^ provided additional validation in an independent cohort with detailed neuropathological characterization, enabling assessment of drug candidates across varying stages of AD pathology. Microglial‐enriched single‐cell RNA‐seq data (Synapse ID: syn52795292)^18^ from both fresh and frozen human brain tissue enabled high‐resolution transcriptional profiling of AD‐associated microglial subtypes, including disease‐associated microglia (GPNMB+), homeostatic microglia (PICALM+), and perivascular macrophages (CD163+).

### 2.3 Data processing and quality control

R software was used for all statistical analyses, and the scRNA‐seq data were processed using the Seurat package (version 4.3.0).^20^ In each dataset, cells that contained fewer than 200 identified genes and genes that were detected in less than three cells were removed. These quality control thresholds represent standard practices widely employed in scRNA‐seq studies.^20^, ^21^ The 200‐gene minimum effectively filters low‐quality cells, empty droplets, and cellular debris while retaining viable cells with adequate transcriptome coverage, while the three‐cell minimum for gene detection removes sparsely expressed genes likely representing technical noise.

After data cleaning, pairwise cell correspondences between diseased and normal cells were constructed by identifying mutual *K*‐nearest neighbors (KNNs) through Seurat's integration workflow. Integration parameters followed Seurat^20^ defaults *k*.anchor = 5 (neighbors for anchor identification), *k*.filter = 200 (neighbors for anchor filtering), and *k*.score = 30 (neighbors for anchor scoring). These parameters represent optimized values validated across diverse scRNA‐seq datasets.^20^, ^21^

Principal component analysis (PCA) was applied using the first 15 principal components for linear dimensional reduction. A KNN graph was constructed in PCA space using *k* = 20 neighbors (Seurat default), which balances local structure preservation with computational efficiency.^20^ The non‐linear dimensional reduction method Uniform Manifold Approximation and Projection (UMAP) was then performed to produce the final low‐dimensional representation of cells.^22^ Pre‐assigned cell types from the provided metadata from each dataset were used to annotate the cells.

### 2.4 Doublet identification

Doublet cells are artifacts that occur when two (or more) individual cells are inadvertently captured and sequenced together as if they were one cell. In standard single‐cell preprocessing, doublets are considered technical artifacts where two cells are inadvertently captured within the same droplet. These technical artifacts are characteristically identified using the Scrublet algorithm and removed to prevent false clustering. However, to explore potential biological cell‐cell interactions, we subsequently performed a targeted doublet re‐annotation analysis following the approach described by Grubman et al.^13^ In this context, “doublet cells” refer not to technical artifacts but to computationally inferred heterotypic cell profiles that reflect mixed transcriptional signatures from distinct cell types (e.g., microglia–endothelial or microglia—oligodendrocyte progenitor cells [OPC] pairs). These “informative doublets” were analyzed separately to infer potential intercellular communication relevant to AD pathophysiology. Recent studies have begun exploring their potential utility for understanding cell‐cell interactions.^20^

Building on this emerging idea, we applied a new approach to re‐annotate doublet cell types based on their top two most likely contributing cell identities to determine if specific cell type interactions in AD brains might drive disease pathology or represent therapeutic targets. The BRETIGEA package (version 1.0.3)^21^ in R was used to annotate the doublet cells within the Grubman et al.^13^ dataset. The BRETIGEA and Seurat packages allow the input of a gene expression matrix and marker genes to estimate a score for each cell type within the doublet cells by calculating the difference of the mean expression levels of each cell type gene set and the expression of the overall gene set.^21^ Then, each cell was annotated based on the two highest cell type scores present that most likely represent the two cells in the doublet.

### 2.5 Drug repurposing analysis

The Limma (version 3.54.1) *R* package was used to generate differential gene expression profiles.^22^ For each cell type, a linear model was fit to identify differentially expressed genes (DEGs) associated with AD. Log_2_ fold‐changes (log_2_FC) and estimated standard errors were used to make pairwise comparisons between the diseased and healthy samples, and Bayesian smoothing was applied to the standard errors.^23^ Profiles were transformed into a gene rank list, including log_2_FC, average expression, *t*‐statistic (log fold‐change over standard error), *p*‐value, Benjamini–Hochberg false discovery rate (FDR) adjusted *p*‐value, and B (log‐odds) for each gene. For downstream analyses, genes were considered significantly differentially expressed if they met dual criteria: statistical significance (*p* < 0.05) and biological significance (|log_2_FC| > 0.58, equivalent to a 1.5‐fold change).

The 21,304 drugs/compounds‐response gene expression profiles in 98 cell lines from the LINCS L1000 project were used to create another gene rank list from the differential gene expression list in response to drug treatment.^16^ To enhance the biological relevance of drug predictions for AD and minimize cell type‐specific confounding effects, we restricted the LINCS L1000 database to include only drug response profiles derived from central nervous system (CNS) cell lines. This tissue‐specific filtering approach ensures that the transcriptional signatures used for drug repurposing predictions better reflect the cellular context of neurological tissue, thereby improving the likelihood that identified drug candidates will exhibit relevant therapeutic effects in the brain. The CNS‐restricted reference profiles were used for all subsequent drug repurposing analyses.

To identify repurposable drugs, we employed a cell type‐specific drug repurposing framework (ASGARD),^24^ which detects consistent DEGs that are either up‐regulated in cells of diseased samples and down‐regulated in cells with treatment, or down‐regulated in cells from diseased samples and up‐regulated in cells with treatment. To evaluate these identified drugs, the *p*‐value, FDR, and ASGARD drug score were compared for each cell type.

The ASGARD drug score estimates drug activity across all cell types using cell type proportion, FDR, and the reversal ratio, defined as the number of DEGs in a given cell type that are reversed by drug treatment divided by the total number of DEGs in that cell type.^24^ Overall, drugs with a higher drug score are predicted to have better therapeutic effects than those with a lower value.

### 2.6 Neuronal cell prioritization

To identify neuronal subpopulations with transcriptomic profiles most strongly associated with AD pathology, we employed a deep transfer learning classification approach (DEGAS).^17^ This machine learning framework learns to distinguish AD from control transcriptomic profiles and assigns each individual cell an AD‐association score based on its similarity to known AD signatures.

Using the Grubman et al.^13^ dataset and Mount Sinai Brain Bank (MSBB)^25^ patient data, we trained the model on 61 AD‐associated gene features identified in previous studies.^17^ First, the counts were log_2_ transformed, converted to sample‐wise *z*‐scores, and standardized to [0,1] per the original DEGAS study.^17^ A five‐fold bootstrap aggregated ClassClass DEGAS model was trained using these data, and the AD‐associations were overlaid onto the Grubman et al.^13^ cells. The ClassClass DEGAS model was designed to distinguish between AD and control transcriptomic profiles by learning discriminative features based on known sample labels.^17^

We extended this cell prioritization approach to three independent datasets, analyzing six distinct neuronal populations using a consistent median‐split stratification strategy. Following DEGAS hazard score assignment, cells from each dataset were stratified within major neuronal cell types to identify DEGs between AD‐associated and healthy‐like neuronal populations.

For the Grubman et al.^13^ dataset, we analyzed the neuronal population as a single group based on the original study's cell type annotations, using the initial DEGAS model trained as described above.

For the Mathys et al.^14^ dataset, we analyzed excitatory (Ex) and inhibitory (In) neurons separately. A five‐fold bootstrap aggregated ClassClass DEGAS model was trained using the Mathys dataset and MSBB patient data following identical preprocessing steps.

For the Green et al.^15^ dataset, we performed separate analyses for three neuronal populations: CUX2‐positive excitatory neurons (CUX2+), CUX2‐negative excitatory neurons (CUX2‐), and inhibitory neurons. A five‐fold bootstrap aggregated ClassClass DEGAS model was trained using the Green dataset and MSBB patient data with the same 61 AD‐associated gene features and standardization procedures.

Within each cell type across all datasets, neurons were stratified by their DEGAS‐derived hazard scores using a median‐split approach. Cells with hazard scores above the median were classified as “high AD‐associated,” while those below the median were classified as “low AD‐associated.” This approach maximizes statistical power while avoiding arbitrary threshold selection, enabling identification of neurons showing early disease‐associated transcriptional changes that are most likely to benefit from therapeutic intervention.

Differential gene expression analysis (*p* < 0.05 and |log_2_FC| > 0.58) was performed using the Seurat package between high and low AD‐associated groups within each cell type. This threshold of |log_2_FC| > 0.58 corresponds to an approximately 1.5‐fold change and ensures both statistical and biological significance. For each significantly dysregulated gene, directionality was assigned based on log_2_FC values: genes with positive values were classified as “Up” (higher expression in high AD‐associated cells), while those with negative values were classified as “Down” (higher expression in low AD‐associated/control‐like cells).

The resulting cell‐type‐specific gene signatures from all six neuronal populations were independently queried against the L1000 Characteristic Direction Signature Search Database (L1000CDS^2^)^26^, ^27^, ^28^ to identify therapeutic compounds capable of reversing AD‐associated transcriptional signatures. For each analysis, we submitted ranked DEG lists and evaluated both reverse queries (identifying compounds whose transcriptomic effects oppose the AD‐associated signature, representing potential therapeutic candidates) and mimic queries (identifying compounds that reproduce the AD‐associated signature, representing potentially harmful agents). The L1000CDS^2^ characteristic direction method computed similarity scores between our DEG signatures and drug perturbation profiles across multiple cell lines, doses, and time points, with compounds ranked by similarity score, where higher scores indicate stronger signature reversal capacity.

### 2.7 Microglial subtype analysis

To characterize microglial and perivascular macrophage heterogeneity in AD, we leveraged the single‐cell RNA sequencing dataset from Lee et al.^18^ See the Availability of Data and Materials section for accession information. This dataset encompassed two independent cohorts: the FreshMG cohort, comprising 543,012 myeloid cells from 137 postmortem brain specimens, and the PsychAD cohort, which includes 289,493 myeloid nuclei from 1470 donors.

To identify microglial subtypes associated with AD, differential expression analysis was performed by applying the Mann–Whitney U test and area under the receiver operating characteristic curve (AUROC) method to rank genes by their specificity for distinct myeloid subtypes. Analysis of gene expression patterns across various tissues and brain regions was performed using data from the Human Protein Atlas (HPA) and Genotype‐Tissue Expression (GTEx) databases to establish the relevance of this gene in AD‐affected brain regions.^29^, ^30^ For each gene of interest, we extracted normalized expression values (transcripts per million [TPM]) and analyzed their distribution patterns across tissues, with a particular focus on brain regions affected in AD (see Supplementary Methods for details).

To assess AD‐related compositional changes, myeloid cell subtype proportions were modeled using a linear mixed effects model accounting for technical and demographic covariates. This analysis allowed for the identification of subtype‐specific transcriptional signatures and their association with AD progression.

### 2.8 Mechanistic pathway analysis

To determine common molecular signatures across TSA‐treated hippocampal neurons, microglial subtypes from the Lee et al.^18^ dataset, and AD‐associated neurons identified via DEGAS analysis, we performed differential expression analysis. DEGs from TSA‐treated neurons were intersected with those from the Lee et al.^18^ microglial subtypes and DEGAS‐defined AD‐associated neurons. Only genes significantly upregulated (log2FC > 0.58 i.e., log2(1.5), FDR < 0.05) in at least two datasets were considered.

### 2.9 Cross‐species validation

RNA‐seq data were used to investigate the effects of histone deacetylase (HDAC) inhibition by TSA in mature hippocampal neurons from mice.^31^ See the Availability of Data and Materials section for accession information. Raw count data comparing TSA and DMSO‐treated samples was imported and processed. Genes expressed at low levels were filtered, and counts were normalized by the trimmed mean of M‐values (TMM) method. Differential gene expression analysis was performed using the voom transformation to stabilize variance, followed by linear modeling with the limma package.^22^ Significant DEGs were identified based on an FDR cutoff of 0.05. Additionally, DEGs were required to have |log_2_FC| > 0.58 to ensure biological relevance, consistent with the thresholds applied throughout this study.

To enable interpretation of the results, Ensembl gene IDs were converted to gene symbols using the biomaRt package.^32^, ^33^ Significant DEGs were visualized with volcano plots, highlighting genes with an FDR < 0.05, and additional labels were applied to genes with a log_2_FC > 8 and *p* < 0.01. This high fold change threshold was selected to prioritize the strongest transcriptional effects, as TSA is known to induce widespread gene expression changes due to its role as an HDAC inhibitor. Enrichment results were obtained via gene ontology (GO) using the clusterProfiler package and visualized with the enrichplot package.^34^, ^35^, ^36^, ^37^

To enable cross‐species comparison of TSA transcriptional effects between mouse hippocampal neurons^31^ and human iPSC‐derived cortical neurons, we performed ortholog mapping using the homologene R package (version 1.4.68.19.3.27). Mouse gene symbols were mapped to human orthologs using the pre‐compiled HomoloGene database, and we retained only 1:1 orthologous pairs to ensure unambiguous gene correspondence. This approach yielded 15,049 1:1 mouse‐human ortholog pairs.

Differential expression analysis results from both datasets were filtered to include only genes with significant changes (*p* < 0.05, |log_2_FC| > 0.58). Genes were classified as upregulated or downregulated based on the direction of log_2_ fold‐change, and conserved genes were defined as those showing significant differential expression in the same direction in both species.

Statistical significance of gene overlap was assessed using the hypergeometric test, with the background gene set defined as all 1:1 orthologs detected in both experiments (*n* = 9333). Correlation analysis was performed using Pearson correlation on log_2_FC values for orthologous genes. GO enrichment analysis of conserved TSA‐responsive genes was performed using clusterProfiler (version 4.0) with the org.Hs.eg.db annotation database, with terms considered significant at p.adjust < 0.05 after Benjamini–Hochberg correction.

### 2.10 Spatial transcriptomics analysis

ST analysis was performed using 10x Genomics Visium data (see the Availability of Data and Materials section for accession information) to investigate the possible regional effects of TSA in the brain. These data were generated using Visium Spatial Gene Expression for formalin fixed & paraffin embedded (FFPE), in combination with immunofluorescence staining on coronal sections of CRND8 and wild‐type (WT) mouse brains at 2.5, 5.7, 13.2, and 17.9 months, per 10x Genomics Application Note protocols. A TSA gene signature, that is, eigengene utilizing the TSA gene set from LINCS, was overlaid onto ST data from mouse brain sections to identify regions with similar gene signatures to TSA. Because TSA was identified as our lead therapeutic candidate from the computational drug repurposing analysis, we utilized this gene signature in the ST data. This approach allowed us to spatially map the potential regional therapeutic targets of TSA within specific brain regions (see Supplementary Methods for details).

### 2.11 Cell culture and treatment

To investigate the transcriptional effects of TSA and Aβ oligomers on human neuronal cells, we conducted RNA sequencing of iPSC‐derived cortical neurons subjected to various treatment conditions. See the Availability of Data and Materials section for accession information. The experimental design included four distinct groups: untreated control, TSA treatment alone, Aβ exposure alone, and combined TSA treatment and Aβ exposure. Each condition was evaluated with four biological replicates, with an additional technical replicate for each condition to assess reproducibility.

Cell culture and treatment were performed as described in the validation studies section using 24‐well‐plates. Following treatment, total RNA was extracted using the Total RNA Purification Kit (Stem Cell Tech, Catalog #79040) according to the manufacturer's protocol.

RNA sequencing data were processed using a comprehensive bioinformatics pipeline. Count matrices were generated using featureCounts and analyzed in *R* using the Seurat package.^38^ Technical replicates showed high correlation and were analyzed both separately and after merging to optimize statistical power. Quality control was performed following standard Seurat workflow procedures. Dimensionality reduction was conducted using PCA followed by UMAP for visualization.

Differential expression analysis was performed using the limma‐voom method with pairwise comparisons between all treatment conditions (Control, TSA‐only, Aβ‐only, and TSA‐plus‐Aβ).^22^ Genes with *p* < 0.05 and absolute log_2_FC > 0.58 were considered significantly differentially expressed.

The resulting gene lists were subjected to pathway enrichment analysis using clusterProfiler with the org.Hs.eg.db annotation database to identify biological processes and molecular functions affected by the treatments. GO terms were considered significant with *p* < 0.05. Particular attention was paid to TSA‐responsive genes and genes of interest across treatment conditions. Heatmaps and visualization of expression patterns were generated using pheatmap and ggplot2 packages.

### 2.12 Viability assays

Human cortical neurons derived from iPSCs (Fujifilm, Catalog #: R1061) were used in this study. The cells were thawed according to the manufacturer's protocol and plated in pre‐coated 96‐well plates and 24‐well plates with poly‐D‐ornithine and Matrigel. Neurons were maintained in BrainPhys media (Catalog #: 05790), with half of the medium exchanged daily.

For experimental treatments, neurons were cultured until in vitro day (DIV) 4, at which point they were treated with various concentrations of TSA (TSA, Sigma–Aldrich, cat. #: T1952) and Aβ oligomers (StressMarq, cat. #: SPR‐488). For TSA treatments, concentrations of 20, 60, and 100 ng/mL were used. For Aβ oligomer exposure, concentrations of 0.2, 1, and 5 µM were tested.^39^ In combined treatment experiments, neurons were pre‐incubated with TSA for 24 h before the addition of Aβ oligomers.

Cell survival was assessed using the CellTiter 96 AQueous One Solution assay (Promega, cat. #: G3582) following the manufacturer's instructions. Neurons were seeded in 96‐well tissue culture plates at a density of 80,000 cells per well. After 24 h of treatment incubation, the media was replaced with 100 µL of fresh culture medium per well, and 20 µL of CellTiter 96 AQueous One Solution was added. The MTS tetrazolium salt in the solution is bioreduced by cells to form a colored formazan product. After 4 h of incubation at 37°C, the absorbance at 490 nm was measured using a plate reader. Viability was expressed as a percentage relative to control wells, and statistical significance was determined using appropriate statistical tests with significance set at *p*‐value < 0.05.

The experimental timeline was as follows: neurons were first pre‐incubated with TSA for 24 h, after which Aβ oligomers were added to the same wells, and cells were maintained for an additional 24 h before assessing viability. Thus, all CellTiter 96 AQueous One Solution (MTS) measurements reflect cumulative viability after 48 h of total exposure (24 h TSA pre‐treatment + 24 h Aβ oligomer incubation).

### 2.13 Immunofluorescence

To evaluate synaptic integrity, the co‐localization of PSD95‐Syn1 positive clusters was assessed at 7 DIV. Neurons were pre‐incubated with 60 ng/mL TSA for 24 h, followed by exposure to 5 µM Aβ oligomers for an additional 24 h. Cells were then fixed in 4% paraformaldehyde and 30% sucrose. Fixed neuron coverslips were washed, permeabilized, and blocked with an animal‐free blocker diluent (Vector Labs, cat. #: SP‐5035).

Fixed neurons were incubated overnight at 4°C in a humidified chamber with primary antibodies: anti‐Synapsin‐1 (1:500, Abcam, cat. #: ab64581) and anti‐PSD95 (1:500, Abcam, cat. #: ab2723). The next day, the coverslips were washed and incubated with the appropriate secondary antibodies, Alexa Fluor 488 and 568 (1:100, Invitrogen, cat. #: A32723 and cat. #: A11036, respectively). The coverslips were examined using a Zeiss LSM 900 confocal microscope. Synaptic cluster quantification was performed with Fiji (ImageJ) as previously described.^39^ The number of synaptic clusters per 20 µm of dendrite was quantified to assess the protective effect of TSA on synaptic structures.

## 3 RESULTS

### 3.1 Multi‐dataset drug repurposing identifies HDAC inhibitors as convergent therapeutic candidates

**FIGURE 2:**
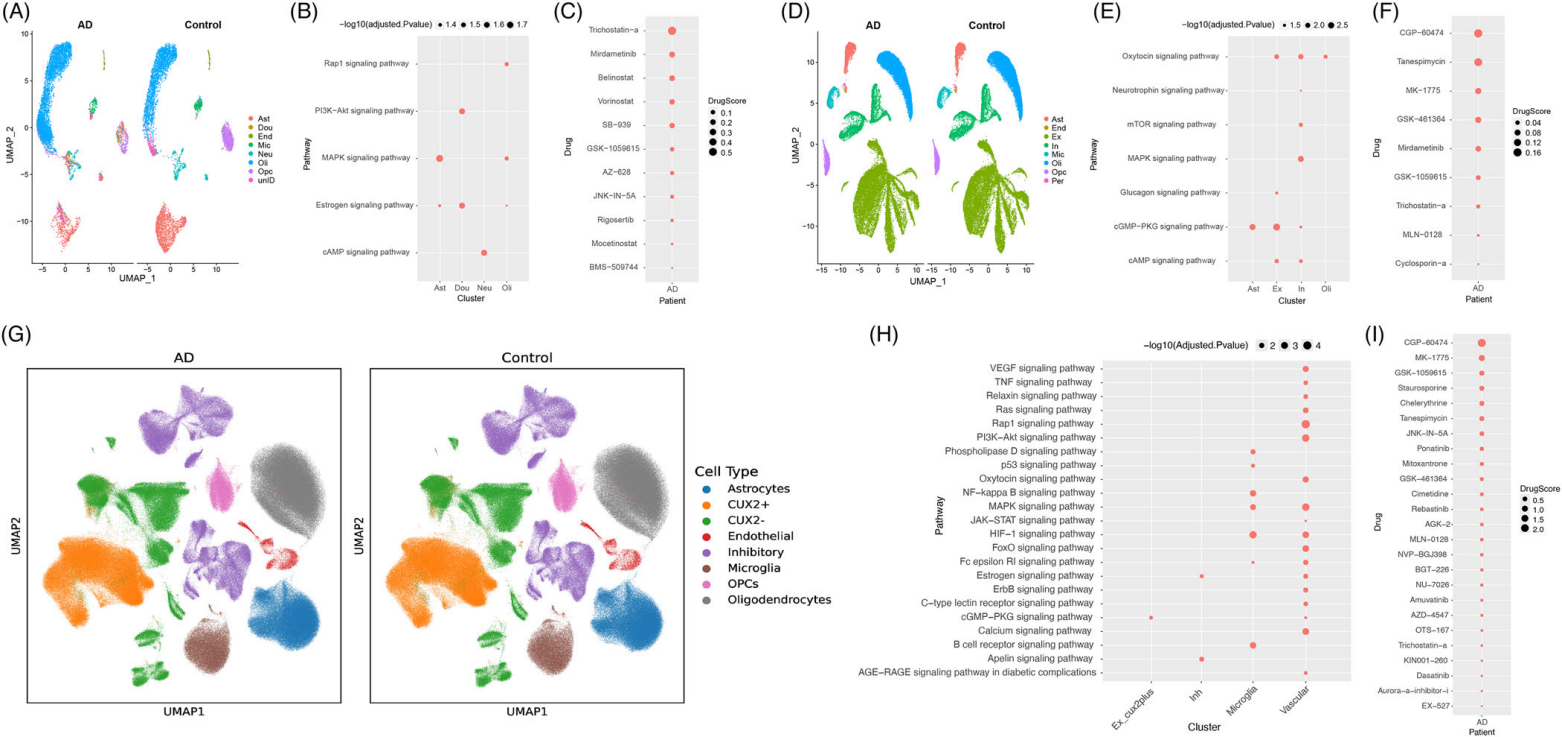
Single‐cell analysis identifies TSA as top drug repurposing candidate across cortical brain regions. (A) UMAP projections of all cells from six control and six AD samples in the Grubman et al.^13^ entorhinal cortex dataset, with clusters representing cell‐type‐specific groupings. (B) Pathway enrichment analysis highlighting key signaling pathways significantly enriched within each cell type cluster in the Grubman dataset, with significance represented as ‐log10(FDR). (C) Drug Score analysis for AD samples in the Grubman dataset, displaying compounds with FDR < 0.1 and Drug Score ranking within the 90th percentile. TSA (trichostatin‐A) emerges among the top‐ranked candidates. (D) UMAP visualizations of all cells from 24 control and 24 AD samples in the Mathys et al.^14^ prefrontal cortex dataset, with distinct clusters representing cell types. (E) Pathway enrichment analysis for the Mathys dataset showing key signaling pathways significantly enriched within each cell type cluster, with significance represented as ‐log10(FDR). (F) Drug Score analysis for AD samples in the Mathys dataset, displaying compounds with FDR < 0.1 and Drug Score values within the 90th percentile. (G) UMAP projections from the Green et al.^15^ aged prefrontal cortex dataset showing cell‐type‐specific clustering across AD and control samples. (H) Pathway enrichment analysis for the Green dataset, highlighting significantly enriched signaling pathways across cell types, with significance represented as ‐log10(FDR). (I) Drug Score analysis for the Green dataset showing top‐ranked therapeutic candidates with FDR < 0.1 and Drug Scores within the 90th percentile. Cell types: Ast, astrocytes; CUX2+, CUX2‐positive excitatory neurons; CUX2‐, CUX2‐negative excitatory neurons; Dou, doublets; End, endothelial cells; Ex, excitatory neurons; In, inhibitory neurons; Inh, inhibitory neurons; Mic, microglia; Neu, neurons; Oli, oligodendrocytes; Opc, oligodendrocyte progenitor cells; Per, pericytes; unID, unidentified cells. AD, Alzheimer's disease; FDR, false discovery rate; TSA, trichostatin‐A; UMAP, Uniform Manifold Approximation and Projection.

The Grubman et al.^13^ dataset includes cells of the entorhinal cortex from control and AD brains of twelve individuals, yielding a total of 13,214 high‐quality nuclei. Grubman et al.^13^ found and annotated 449 microglia, 2171 astrocytes, 656 neurons, 7432 oligodendrocytes, 1078 oligodendrocyte progenitor cells, 98 endothelial cells, 925 unidentified cells, and 405 hybrid cells. Oligodendrocytes are the largest cluster, accounting for 56.24% of cells, whereas endothelial cells are the smallest cluster, making up 0.74% of cells (Figure 2A). The DEGs are significantly enriched in five pathways, including Rap1, phosphatidylinositol 3‐kinase (PI3K)‐AKT, mitogen‐activated protein kinase (MAPK), estrogen, and cAMP signaling pathways (Figure 2B). The MAPK signaling pathway is the most significant pathway in this dataset, with ‐log10(FDR) of 1.79 in astrocytes.

Eleven compounds were predicted with significant FDR values (< 0.1) for AD using the calculated drug score and a quantile cutoff of 0.9 (Figure 2C). The top drug candidate identified is TSA, an HDAC inhibitor, with a drug score of 0.57 and significant FDR values (< 0.1) in the neuron, doublet, and oligodendrocyte cell types (Table S1). Notably, multiple additional HDAC inhibitors emerged among the top candidates, including SB‐939 (pracinostat), vorinostat (SAHA), and belinostat, all of which are pan‐HDAC inhibitors with distinct selectivity profiles. This convergence of multiple structurally distinct HDAC inhibitors as independent top candidates strongly implicates HDAC inhibition as a therapeutically relevant mechanism for AD intervention. Other significant treatments outside the HDAC inhibitor class include JNK‐IN‐5A (a c‐Jun N‐terminal kinase [JNK] inhibitor, drug score = 0.21), Mirdametinib (a MEK inhibitor, drug score = 0.19), GSK‐1059615 (a dual PI3K/ mechanistic target of rapamycin [mTOR] inhibitor, drug score = 0.17), AZ‐628 (a RAF kinase inhibitor, drug score = 0.14), and rigosertib (a multi‐kinase inhibitor, drug score = 0.13) (Table S1).

The Mathys et al.^14^ dataset includes 48 prefrontal cortex samples from 24 individuals with high levels of Aβ and other pathological hallmarks of AD and 24 individuals with no or very low Aβ burden or other pathologies, resulting in 80,660 droplet‐based scRNA‐seq profiles. Excitatory neurons are the largest cluster, accounting for 49.52% of cells, whereas endothelial cells were the smallest cluster, making up 0.17% of cells (Figure 2D). The DEGs are significantly enriched in seven pathways, including cGMP‐PKG, Oxytocin, cAMP, Glucagon, MAPK, mTOR, and Neurotrophin signaling pathways (Figure 2E). The cyclic guanosine monophosphate‐protein kinase G (cGMP‐PKG) signaling pathway is the most significant, with ‐log10(FDR) of 2.90, 2.41, and 1.47 in the excitatory neurons, astrocytes, and inhibitory neurons, respectively.

Nine compounds with significant FDR values (< 0.1) for AD were predicted using the calculated drug score and a quantile cutoff of 0.9 (Figure 2F). The top drug candidate identified is CGP‐60474, a cyclin‐dependent kinase (CDK) inhibitor, with a drug score of 0.17 and a significant FDR value (< 0.1) in oligodendrocyte progenitor cells (Table S2). CGP‐60474 is a small molecule that has been shown to be associated with regulating cell cycle transitions.^28^ TSA also emerged as a drug candidate using the Mathys et al.^14^ dataset with a drug score of 0.01 and significant FDR values in multiple cell types. Furthermore, mirdametinib (drug score = 0.09) and GSK‐1059615 (drug score = 0.08) also appear as candidates in both the Grubman et al.^13^ and Mathys et al.^14^ datasets. Other significant compounds include MK‐1775 (a WEE1 kinase inhibitor, drug score = 0.12), tanespimycin (a HSP90 inhibitor, drug score = 0.10), and MLN‐0128 (an mTOR kinase inhibitor, drug score = 0.07). A complete summary of all compounds with significant FDR (< 0.1) identified from the Mathys et al.^14^ dataset, including their drug scores and associated cell types, is provided in Table S2.

The Green et al.^15^ dataset includes 48 post‐mortem human brain samples from individuals with varying levels of AD pathology, yielding a total of 1,173,122 high‐quality nuclei from prefrontal cortex samples. Green et al.^15^ identified and annotated seven major brain cell types: astrocytes, CUX2+ excitatory neurons, CUX2‐ excitatory neurons, inhibitory neurons, microglia, oligodendrocytes, and vascular cells (Figure 2G). The DEGs were significantly enriched in multiple pathways across different cell types (Figure 2H). The Rap1 signaling pathway was the most significant pathway in this dataset. Notably, the MAPK signaling pathway showed enrichment in both microglia and vascular cells, consistent with its identification as significant in the Grubman and Mathys datasets.

Twenty‐five distinct compounds emerged as top candidates with significant FDR values (< 0.1) for AD using the calculated drug score and a quantile cutoff of 0.9 (Figure 2I). The top drug candidate identified was CGP‐60474, with a drug therapeutic score of 2.43 and highly significant FDR values, showing broad activity across neuronal and glial cell types. Other highly ranked candidates included MK‐1775 (drug score = 1.07), GSK‐1059615 (drug score = 0.67), chelerythrine (drug score = 0.56), tanespimycin (drug score = 0.54), staurosporine (drug score = 0.51), and JNK‐IN‐5A (drug score = 0.49). Notably, the top‐ranked compounds showed particular enrichment in oligodendrocytes, with CGP‐60474, MK‐1775, chelerythrine, JNK‐IN‐5A, tanespimycin, and staurosporine all achieving significance in this cell type (Table S3).

HDAC inhibitors also emerged as candidates in the Green dataset. SB‐939 (pracinostat, drug score = 0.040), belinostat (drug score = 0.037), and vorinostat (SAHA, drug score = 0.031) all showed significance specifically in microglial populations. TSA achieved significance with a drug therapeutic score of 0.053. The identification of multiple structurally distinct HDAC inhibitors across all three independent datasets, despite substantial methodological differences, diverse brain regions, and varied cohort characteristics, provides strong evidence for HDAC inhibition as a therapeutically relevant mechanism for AD intervention (Table S3).

### 3.2 Cross‐dataset validation reveals TSA as a top convergent candidate with consistent pathway targeting

TSA was one of only three compounds to achieve statistical significance (FDR < 0.1) across all three independent datasets, alongside CGP‐60474 and GSK‐1059615 (Table S4). This convergence, despite substantial methodological differences, diverse brain regions, and varied cohort characteristics, provides strong evidence for their therapeutic potential. While TSA ranked 1st in the Grubman dataset (drug score = 0.569), 11th in the Mathys dataset (drug score = 0.034), and 26th of 865 tested compounds in the Green dataset (drug score = 0.053), the consistent statistical significance across all three studies is remarkable given the differences in cellular composition, disease stage heterogeneity, and brain regions analyzed (entorhinal cortex vs. prefrontal cortex).

The substantial variation in TSA's drug therapeutic scores across datasets (ranging from 0.034 to 0.569) likely reflects differences in cellular composition, disease stage heterogeneity, and the specific cell types driving the therapeutic signal across brain regions and cohorts. In the Grubman dataset, TSA showed significant FDR values in neurons, doublets, and oligodendrocytes. In contrast, the Green dataset demonstrated TSA activity across a broader range of cell types. This variation in cell‐type specificity across datasets may contribute to the observed differences in overall drug scores while maintaining statistical significance.

GSK‐1059615 demonstrated remarkable cell‐type specificity in astrocytes, the most significant association for any compound in any single cell type across all datasets. Its consistent identification across all three datasets in both astrocytes and oligodendrocyte progenitor cells reinforces its potential therapeutic relevance. CGP‐60474, the top‐ranked candidate in both the Green (drug score = 2.43) and Mathys (drug score = 0.17) datasets, showed broad activity across neuronal and glial cell types with 79.77% drug coverage across cell types in the Green dataset, further supporting its investigation as a therapeutic target.

### 3.3 Pathway analysis reveals shared dysregulation of cAMP, MAPK, and neurotrophin signaling across brain regions

Multiple signaling pathways demonstrated enrichment across datasets, revealing common molecular mechanisms underlying AD pathology in different brain regions. The MAPK signaling pathway was significantly enriched across all three datasets, identified in astrocytes in the Grubman dataset, across multiple cell types in the Mathys dataset, and in both microglia and vascular cells in the Green dataset. The MAPK signaling pathway involves the extracellular signal‐regulated kinase (ERK), JNK, and p38 pathways, which regulate cell proliferation, differentiation, development, transformation, and death (Figure S1).^40^

The cAMP signaling pathway showed enrichment in both the Grubman and Mathys datasets, while the related cGMP‐PKG signaling pathway was the most significant pathway in the Mathys dataset, with ‐log_10_(FDR) of 2.90, 2.41, and 1.47 in excitatory neurons, astrocytes, and inhibitory neurons, respectively. The cGMP‐PKG signaling pathway regulates cellular processes such as vascular smooth muscle contraction, reduced cardiac hypertrophy, and inhibition of cellular apoptosis (Figure S2).^41^ Notably, the cGMP‐PKG signaling pathway also showed enrichment in CUX2+ excitatory neurons in the Green dataset, consistent with the Mathys dataset, where this pathway was most significantly enriched in excitatory neurons, further supporting the role of cGMP‐PKG signaling dysregulation in excitatory neuronal dysfunction in the prefrontal cortex.

The neurotrophin signaling pathway was identified as significantly enriched in the Mathys dataset, while the related PI3K‐Akt signaling pathway showed enrichment in both the Grubman dataset and extensively in vascular cells of the Green dataset. Vascular cells in the Green dataset showed extensive pathway dysregulation, with 18 significantly enriched pathways including MAPK signaling, PI3K‐Akt signaling, Calcium signaling, FoxO signaling, HIF‐1 signaling, and Oxytocin signaling pathways, among others. Microglia displayed enrichment in immune‐related and stress‐response pathways, with HIF‐1 signaling, B cell receptor signaling, NF‐kappa B signaling, and MAPK signaling showing the strongest enrichments.

The convergence on MAPK/ERK pathway inhibitors (JNK‐IN‐5A and Mirdametinib) and PI3K/mTOR pathway modulators (GSK‐1059615 and MLN‐0128) across datasets strengthens the evidence for these pathways as therapeutic targets in AD. Mirdametinib, a MEK inhibitor targeting the MAPK/ERK pathway, achieved significance in both the Grubman (drug score = 0.19) and Mathys (drug score = 0.09) datasets, directly linking the observed pathway dysregulation to potential therapeutic intervention.

### 3.4 TSA and mirdametinib exhibit distinct cell‐type targeting profiles across brain regions

**FIGURE 3:**
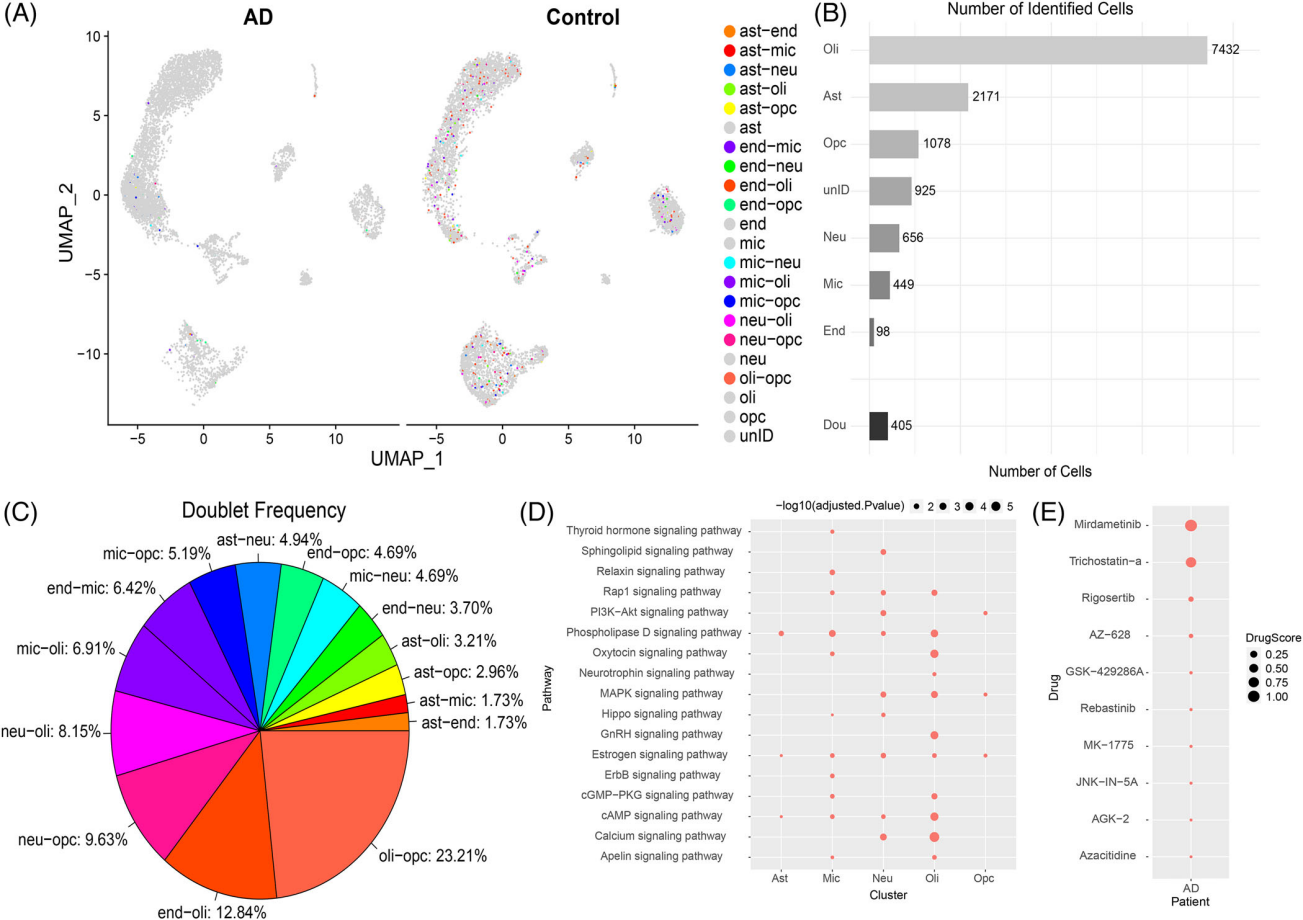
Doublet cell analysis reveals cellular interaction patterns and drug targeting opportunities in AD. (A) UMAP projections of all cells from six control and six AD samples in the Grubman et al.^13^ dataset, showing cell‐type‐specific clusters with doublets re‐annotated into their most likely two contributing cell types (e.g., ast‐mic for astrocyte–microglia doublets). (B) Bar chart displaying the counts of identified cell types, including doublets without splitting into their component cell types. (C) Pie chart illustrating the distribution of identified doublet cell types, providing an overview of the most common cellular interactions observed in the dataset. (D) Pathway enrichment analysis for re‐annotated cell type clusters, highlighting key signaling pathways with significant enrichment, represented as ‐log10(FDR). (E) Drug Score analysis for AD samples, highlighting compounds with FDR < 0.1 and Drug Scores within the 90th percentile. TSA shows particularly strong enrichment in doublet populations involving microglia. Cell types: Ast, astrocytes; Dou, doublets; End, endothelial cells; Mic, microglia; Neu, neurons; Oli, oligodendrocytes; Opc, oligodendrocyte progenitor cells; unID, unidentified cells. AD, Alzheimer's disease; FDR, false discovery rate; UMAP, Uniform Manifold Approximation and Projection.

To investigate the cellular specificity of therapeutic candidates and explore potential cell‐cell interaction contexts relevant to AD pathology, doublet cells from the Grubman et al.^13^ dataset were analyzed to identify significant cell type interactions (Figure 3A). This approach represents an application of doublet cell analysis, not merely treating doublets as technical artifacts but leveraging them as a potential source of insight into cell‐cell interactions. This strategy is particularly relevant in the context of AD, where dysregulated cellular communication may contribute to disease progression or represent therapeutic entry points. The doublet cells make up 405 of the 13,214 cells in the Grubman et al.^13^ dataset (Figure 3B). After re‐annotating the doublet cell types as their top two interacting cells, the oligodendrocytes are still the largest cluster, accounting for 56.24% of cells, whereas astrocyte‐microglia and astrocyte‐endothelial cells are the smallest clusters, each making up approximately 0.05% of cells (Figure 3C). Of the doublet cells, the oligodendrocyte–oligodendrocyte progenitor, endothelial–oligodendrocyte, and neuron–oligodendrocyte progenitor cells are the largest three clusters, making up approximately 23.2, 12.8, and 9.6 percent of doublets, respectively (Figure 3C).

**FIGURE 4:**
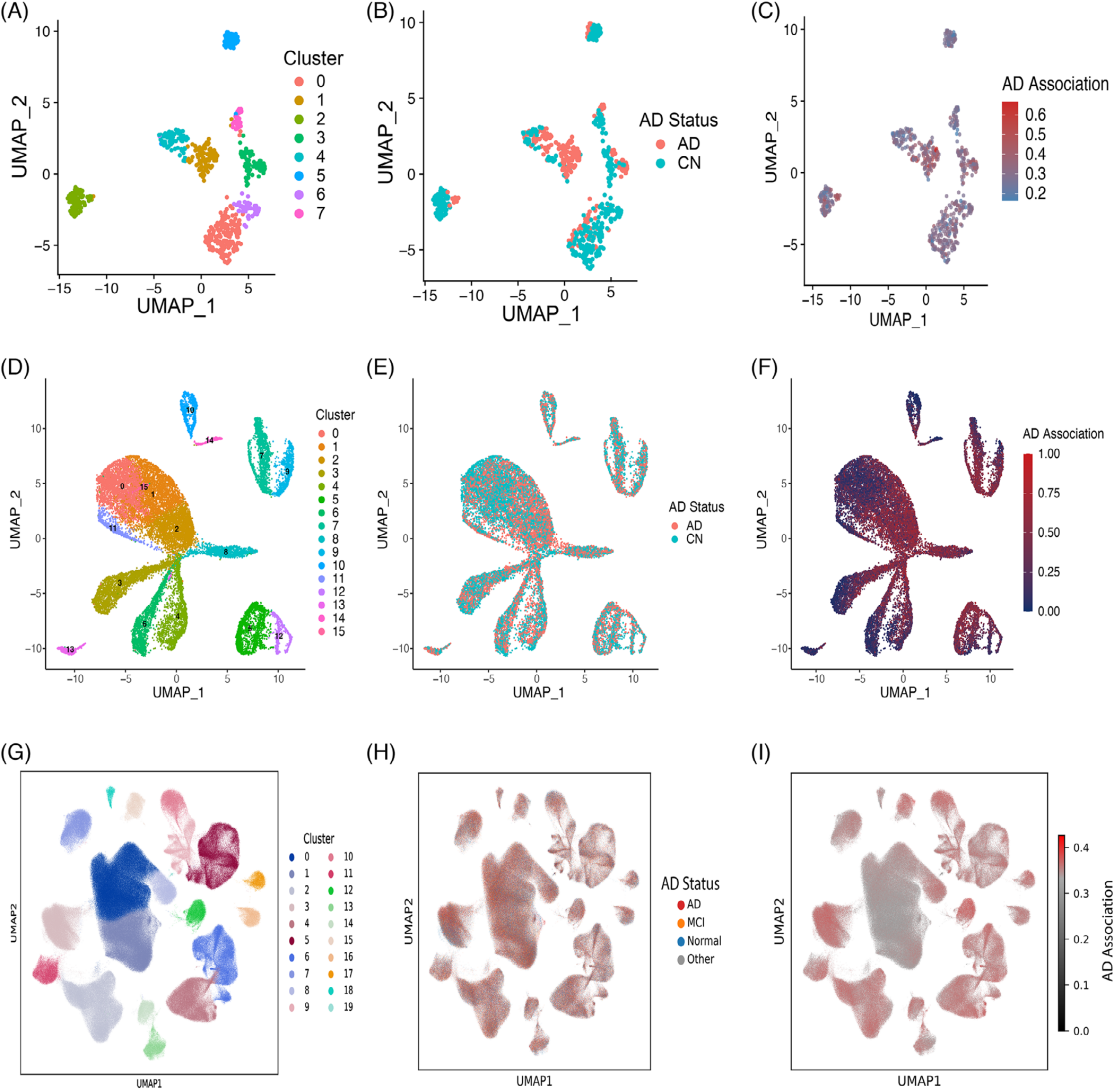
Machine learning‐based cell prioritization identifies AD‐associated neuronal subpopulations across three independent datasets. (A–C) Cell prioritization analysis of neurons from the Grubman et al. entorhinal cortex dataset. (A) UMAP projection showing eight neuronal clusters (0–7) identified by unsupervised clustering. (B) Neurons colored by clinical diagnosis, showing distribution of AD (red) and cognitively normal (CN, blue) samples across the UMAP space. (C) Machine learning‐predicted AD association scores for each neuron, where higher scores (red) indicate stronger transcriptomic similarity to AD‐associated expression patterns, while lower scores (blue) indicate greater similarity to cognitively normal patterns. (D–F) Cell prioritization analysis of neurons from the Mathys et al. prefrontal cortex dataset. (D) UMAP projection displaying 16 neuronal clusters (0–15) identified through unsupervised clustering, demonstrating greater neuronal heterogeneity in this larger dataset. (E) Neurons colored by clinical diagnosis (AD in red, CN in blue), showing intermixed disease states across the transcriptomic landscape. (F) Machine learning‐predicted AD association scores ranging from 0.00 (low AD association, blue) to 1.00 (high AD association, red). The scoring reveals distinct subpopulations with varying degrees of disease association independent of clinical diagnosis. (G–I) Cell prioritization analysis of neurons from the Green et al. aged prefrontal cortex dataset. (G) UMAP projection showing 20 neuronal clusters (0‐19), reflecting the complexity and diversity of neuronal populations in this aged cohort. (H) Neurons colored by clinical classification, including AD (red), MCI (orange), normal (blue), and other (gray), demonstrating the representation of multiple disease stages. (I) Machine learning‐predicted AD association scores ranging from 0.0 to 0.4, with higher scores (red/brown) indicating greater AD‐associated transcriptomic signatures. The reproducibility of AD association scoring across three independent datasets from different brain regions, cohorts, and disease stages validates the robustness of the DEGAS cell prioritization approach for identifying vulnerable neuronal subpopulations in AD. AD, Alzheimer's disease; CN, cognitively normal; MCI, mild cognitive impairment; UMAP, Uniform Manifold Approximation and Projection.

The DEGs from the doublet analysis are significantly enriched in 17 pathways, including Calcium, cAMP, Oxytocin, GnRH, Phospholipase D, MAPK, cGMP‐PKG, Rap1, estrogen, Apelin, neurotrophin, PI3K‐AKT, sphingolipid, Hippo, Relaxin, ErbB, and Thyroid hormone signaling pathways (Figure 4D). The calcium signaling pathway is the most significant pathway in this dataset, with ‐log_10_(FDR) of 5.63 and 2.78 in the oligodendrocytes and neurons, respectively. Calcium signaling plays a fundamental role in regulating a wide range of cellular processes, including proliferation, differentiation, metabolism, gene expression, and apoptosis (Figure S3).^41^

After identifying and labeling the doublet cells as their top two cell types, 10 drugs with significant FDR values (< 0.1) for AD were predicted using the calculated drug score and a quantile cutoff of 0.9 (Figure 3E). The top drug candidate identified is Mirdametinib with a drug score of 1.07. Mirdametinib is a small‐molecule inhibitor of the MAPK/ERK kinase, which prevents activation of the mitogen‐activated protein kinase (MAPK). This drug shows significance in oligodendrocyte progenitor, unidentified, microglia, astrocytes, neuron‐oligodendrocyte progenitor, endothelial‐microglia, and oligodendrocyte cell types (Table S5). Consistent with findings from the standard cell type analysis, TSA and other HDAC inhibitors (including mocetinostat and entinostat) were again identified in the doublet analysis, with TSA showing significant activity across multiple cell type interactions, including endothelial‐microglia, microglia‐oligodendrocyte progenitor, and endothelial‐oligodendrocyte progenitor doublets. TSA was also identified as significant in unidentified microglia‐oligodendrocyte progenitor, endothelial‐oligodendrocyte progenitor, oligodendrocyte, endothelial, and oligodendrocyte progenitor cells (Table S5). The repeated identification of HDAC inhibitors across both standard and doublet‐based analytical approaches provides additional evidence for the robustness of HDAC inhibition as a therapeutic strategy in AD.

Comparative analysis across the Grubman et al.^13^ entorhinal cortex dataset, the Mathys et al.^14^ prefrontal cortex dataset, and the Green et al.^15^ prefrontal cortex dataset identified CGP‐60474, GSK‐1059615, and TSA as potential repurposable drugs achieving significance across multiple brain regions and cohorts (Table S6). Cell type‐specific analysis revealed distinct targeting patterns. CGP‐60474 showed consistent activity across Mathys oligodendrocyte precursor cells and multiple Green neuronal populations (excitatory neurons, oligodendrocytes, and inhibitory neurons), while GSK‐1059615 targeted oligodendrocyte precursor cells in Mathys and astrocytes in Green. TSA demonstrated broad cellular coverage in the Grubman dataset, affecting oligodendrocytes and multiple rare cell populations, with additional activity in Mathys oligodendrocyte precursor cells (Table S6).

Pathway enrichment analysis identified three core signaling pathways (MAPK, oxytocin, and cGMP‐PKG signaling) as significantly dysregulated across all three independent datasets, representing the most consistently affected mechanisms in AD pathology (Table S7). An additional four pathways showed dysregulation in two of three datasets. Estrogen, PI3K‐Akt, and Rap1 signaling pathways were enriched in both Grubman and Green datasets, while cAMP signaling was dysregulated in Grubman and Mathys (Table S7). Notably, vascular cells in the Green dataset exhibited particularly strong enrichment across multiple pathways (MAPK, Oxytocin, PI3K‐Akt, Rap1), with ‐log_10_(FDR) values ranging from 1.89 to 4.20, highlighting potential vascular contributions to AD pathology that may be underappreciated in existing therapeutic strategies (Table S7). The identification of shared pathways across both entorhinal cortex and prefrontal cortex, validated in three independent cohorts spanning different disease stages and demographic characteristics, suggests that dysregulation of these signaling mechanisms represents a conserved and reproducible feature of AD pathology, further strengthening the therapeutic rationale for targeting these pathways.

To assess the spatial distribution of TSA therapeutic effects across brain regions, spatial transcriptomics analysis using a TSA‐associated gene signature revealed distinct enrichment patterns. The hippocampus and cerebral cortex showed the strongest enrichment, suggesting these regions as key areas for the potential therapeutic effects of TSA in AD (Figure S4). This spatial enrichment pattern is consistent with the known vulnerability of these regions to AD pathology and supports the targeting of these areas for TSA‐based therapeutic intervention. See Supplementary Results for comprehensive spatial transcriptomics analysis.

### 3.5 Cell‐prioritization identifies AD‐associated neuronal subpopulations with distinct vulnerability profiles

Based on the Grubman et al.^13^ data, eight neuronal clusters were identified (Figure 4A), encompassing neurons derived from AD and cognitively normal patients (Figure 4B). Using a cell prioritization approach trained on known AD transcriptomic signatures, we assigned each neuron an AD‐association score reflecting its similarity to disease‐associated expression patterns. This cell‐level scoring revealed distinct neuronal subpopulations with varying degrees of AD‐association within the same tissue sample (Figure 4C).

After differential expression was performed between the highest 50% AD‐associated neurons (median‐split stratification) and the lowest 50% AD‐associated neurons, 410 upregulated and 512 downregulated genes were identified. Analysis of the Grubman et al.^13^ dataset revealed strong enrichment for HDAC inhibitors across neuronal populations (Files S1–S3). When these genes were queried against the L1000CDS^2^ database using mimic queries (identifying compounds that reproduce the disease signature), vorinostat emerged as the #1 ranked compound (score = 0.0484), followed by TSA at rank #2 (score = 0.0401) (File S1). Notably, HDAC inhibitors did not appear among the top‐ranked compounds in reverse queries for this entorhinal cortex dataset, representing a distinct pattern compared to prefrontal cortex analyses (File S2). This observation demonstrates that cellular prioritization tools can provide more robust or complementary results compared to bulk differential expression analysis and may reveal region‐specific or stage‐dependent therapeutic mechanisms.

To assess the reproducibility of our cell prioritization findings across independent cohorts and brain regions, we performed parallel median‐split analyses of excitatory and inhibitory neurons in the Mathys et al.^14^ prefrontal cortex dataset (Figure 4D–F) and three neuronal populations in the Green et al.^15^ aged prefrontal cortex dataset (Figure 4G–I).

Analysis of the Mathys et al.^14^ dataset revealed particularly strong enrichment for HDAC inhibitors across both excitatory and inhibitory neuronal populations (Files S4–S9). In excitatory neurons, vorinostat ranked #2 (score = 0.0317) and #4 (score = 0.0299), and TSA ranked #5 (score = 0.0299) and #6 (score = 0.0280) in reverse queries, with multiple additional TSA and vorinostat signatures appearing in the top 50 compounds. HDAC6 inhibitor ISOX also appeared at rank #10 (score = 0.0280) (File S5). Differential expression identified 130 upregulated genes including mitochondrial transcripts (MTRNR2L8, MTRNR2L1, MTRNR2L12), metabolic enzymes (DHFR), and signaling molecules (RASD2, RASGEF1B, LRFN4), alongside 698 downregulated genes including transcription factors (ETV1, NEUROD6), extracellular matrix components (COL24A1), and Y‐chromosome‐linked genes (SPDYE1, SPDYE2, SPDYE3), showing patterns consistent with impaired neuronal specification, extracellular matrix remodeling, and cellular stress responses characteristic of AD pathology (File S6).

In inhibitory neurons, TSA achieved the #1 and #2 ranked positions (score = 0.0364) in L1000CDS^2^ reverse queries (identifying compounds that oppose the disease signature), with vorinostat at rank #3 (score = 0.0350), representing the strongest HDAC inhibitor signals observed across all cell‐type‐specific analyses (File S8). Differential expression analysis identified 90 significantly upregulated genes, including mitochondrial transcripts (MTRNR2L8, MTRNR2L12), metabolic regulators (DHFR, RASD2), and transcription factors (TSHZ2, MAF, SMAD2), alongside 821 downregulated genes including the inhibitory neuron marker somatostatin (SST), stress hormones (CRH), interneuron markers (LAMP5), and transcriptional regulators (ETV1, ID2), indicating widespread loss of inhibitory neuron identity markers and functional specialization in AD‐associated cells (File S9).

The Green et al.^15^ dataset provided additional validation across three neuronal populations from an independent cohort (Files S10–S18). In CUX2‐positive excitatory neurons, vorinostat appeared at rank #2 (score = 0.2353), with TSA at rank #6 (score = 0.1765) and belinostat at rank #17 (File S11). Multiple additional TSA and vorinostat signatures populated the top 50 hits (Files S10–S18).

Differential expression identified only 3 upregulated genes in high AD‐associated neurons: QKI (RNA‐binding protein involved in oligodendrocyte function and myelination), MALAT1 (long non‐coding RNA regulating gene expression and splicing), and DOCK2 (immune cell regulator), alongside 31 downregulated genes including cytoskeletal components (ACTG1, TUBA1B), metabolic enzymes (ALDOC), and structural proteins (STMN2, PID1), indicating impaired neuronal cytoskeletal integrity and metabolic dysfunction in AD‐associated neurons (File S12).

CUX2‐negative excitatory neurons showed vorinostat at rank #3 (score = 0.2308) and HDAC6 inhibitor ISOX at rank #2 (File S14). Differential expression revealed six upregulated genes including extracellular matrix components (EDIL3, FN1), long non‐coding RNAs (MALAT1, NEAT1), and immune‐related factors (HVCN1, MAP3K20), alongside 26 downregulated genes enriched in cytoskeletal proteins (ACTG1, TUBA1B) and signaling molecules (DKK3, DIRAS2, PPEF1), displaying similar patterns of structural and functional dysregulation (File S15).

Inhibitory neurons demonstrated vorinostat at rank #2 (score = 0.2353) among top candidates (File S17). Differential expression identified 10 upregulated genes, including extracellular matrix and vascular factors (EDIL3, FN1, NPNT, ANGPT1), long non‐coding RNAs (NEAT1, MALAT1), and growth factors (IGFBP5), alongside 32 downregulated genes notably enriched in myelin‐associated proteins (MBP, MOBP), ion transport (ATP1A2), and glial metabolic markers (GLUL, SLC1A2), suggesting altered glial‐neuronal interactions and myelination in AD‐associated inhibitory neuron populations (File S18). While sharing some common dysregulated genes with excitatory populations, inhibitory neurons showed unique patterns in genes related to gamma‐aminobutyric acid‐ergic (GABAergic) neurotransmission and interneuron‐specific functions, indicating both shared and cell‐type‐specific responses to AD pathology across neuronal subtypes.

Cell prioritization across datasets demonstrated reproducible HDAC inhibitor enrichment in AD‐associated neuronal populations despite regional and cohort differences (Figure S5).

### 3.6 TSA induces significant transcriptional changes in hippocampal neurons with conserved effects across species

**FIGURE 5:**
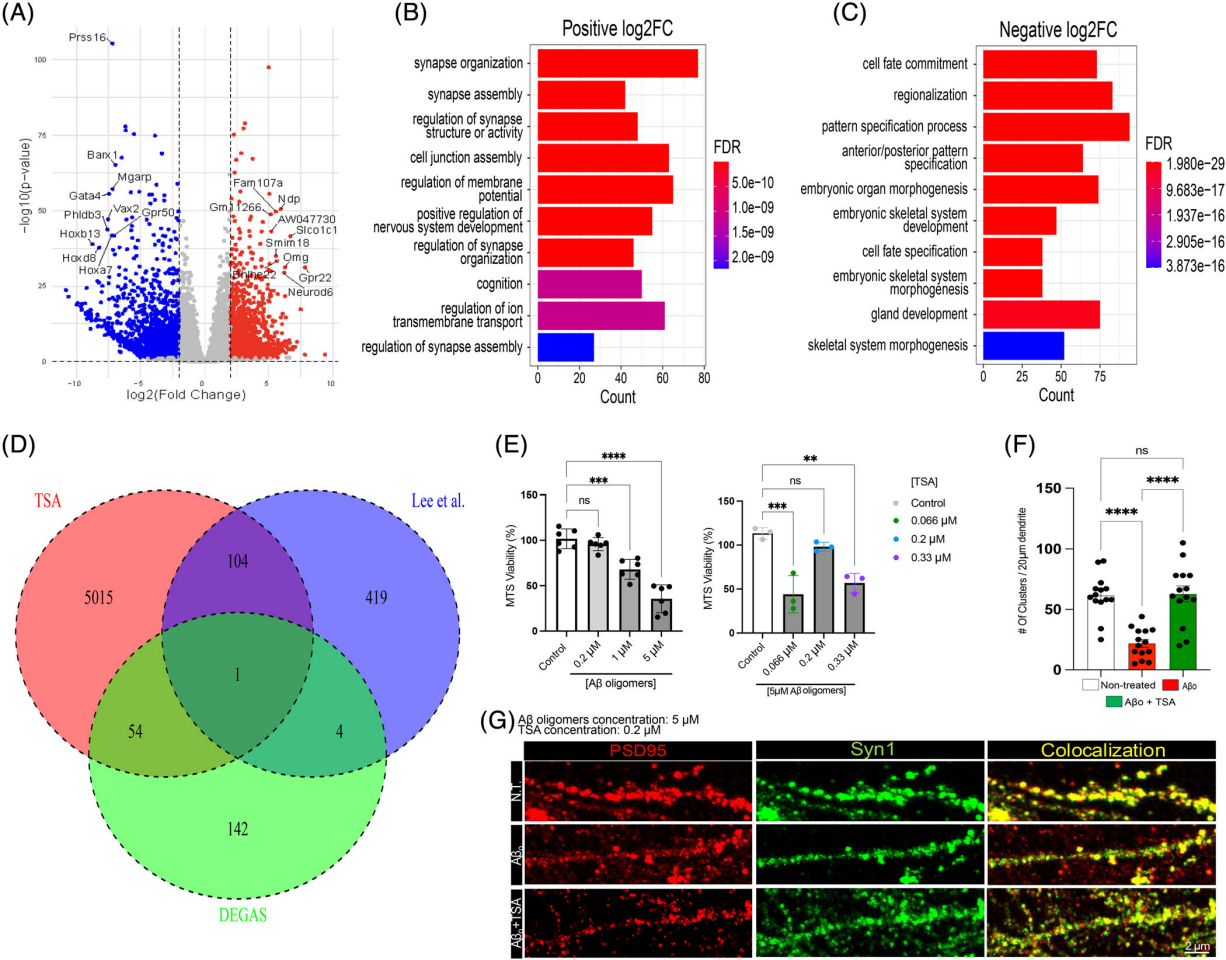
TSA modulates synaptic and developmental gene programs and prevents Aβ‐induced neurotoxicity in human iPSC‐derived cortical neurons. (A) Volcano plot of DEGs in TSA‐treated mouse hippocampal neurons versus control. Blue points represent significantly downregulated genes, while red points represent significantly upregulated genes (adjusted *p*‐value < 0.05). (B) GO Biological Process enrichment analysis for genes with positive log2 fold change (upregulated by TSA treatment). (C) GO Biological Process enrichment analysis for genes with negative log2 fold change (downregulated by TSA treatment). (D) Venn diagram showing overlapping upregulated genes across three independent analyses: TSA‐treated mouse hippocampal neurons (red, 5015 unique genes), microglial subtypes from the Lee et al. human dataset (blue, 419 unique genes), and AD‐associated neurons identified via DEGAS cell prioritization analysis (green, 142 unique genes). The diagram reveals 104 genes shared between the TSA and Lee datasets, 54 genes shared between the TSA and DEGAS, 4 genes shared between Lee and DEGAS, and critically, 1 gene (DISC1) upregulated across all three experimental contexts, identifying it as a convergent therapeutic target. (E) MTS cell viability assay results in human iPSC‐derived cortical neurons. Left panel: Dose‐response curve showing Aβ oligomer‐induced toxicity at concentrations of 0.2, 1, and 5 µM compared to control. Right panel: TSA neuroprotection against 5 µM Aβ oligomers, with neurons pre‐treated with varying TSA concentrations (0.066, 0.2, 0.33) showing dose‐dependent rescue of cell viability. Statistical significance: ns (not significant), ^**^ *p* < 0.01, ^***^ *p* < 0.001, ^****^ *p* < 0.0001. (F) Quantification of synaptic cluster density (number of clusters per 20 µm dendrite) across treatment conditions. NT neurons show baseline synaptic density (gray), 5 µM) cause significant synaptic loss (red), and co‐treatment with 0.2 µM TSA (Aβo + TSA, green) significantly rescues synaptic density, demonstrating TSA's protective effect on synaptic integrity. Each dot represents an individual measurement. Statistical significance: ns (not significant), ****p < 0.0001. (G) Representative confocal immunofluorescence images of synapses in human iPSC‐derived cortical neurons. Neurons were immunostained for the postsynaptic marker PSD95 (red, left column) and presynaptic marker Syn1 (green, middle column), with colocalization (yellow, right column) indicating functional synapses. Rows show: NT controls (top), 5 µM Aβo treatment (middle), and combined treatment with Aβo plus 0.2 µM TSA (Aβo+TSA, bottom). TSA treatment preserves synaptic density and colocalization despite Aβ exposure. Scale bar = 2 µm. Aβ, β‐amyloid; Aβo, Aβ oligomers; AD, Alzheimer's disease; DEG, differentially expressed genes; DEGAS, Diagnostic Evidence Gauge of Single cells; GO, gene ontology; iPSC, induced pluripotent stem cell; NT, non‐treated; TSA, trichostatin‐A.

To assess the transcriptional effects of TSA treatment on hippocampal neurons, we performed differential gene expression analysis comparing TSA‐treated neurons to dimethyl sulfoxide (DMSO) ‐treated controls.^31^ This analysis revealed a robust transcriptional response to TSA treatment (Figure 5A). Among the most significantly upregulated genes, we identified GPR22 (log2FC = 7.83, FDR < 0.001), SLCO1C1 (log_2_FC = 6.68, FDR < 0.001), NDP (log_2_FC = 5.93, FDR < 0.001), and KAT2B (log_2_FC = 2.00, FDR < 0.01) (File S19). Conversely, among the most significantly downregulated genes, we found PHLDB3 (log_2_FC = −7.58, FDR < 0.001), GATA4 (log_2_FC = −7.47, FDR < 0.001), and BARX1 (log_2_FC = −6.97, FDR < 0.001) (File S19).

Gene ontology enrichment analysis of positively regulated genes identified significant overrepresentation of biological processes associated with neuronal differentiation, pattern specification, cell fate commitment, and regionalization (Figure 5B). These pathways are essential for neuronal repair, synaptic remodeling, and neurogenesis, supporting TSA's role in enhancing neurodevelopmental programs through epigenetic regulation. Gene ontology enrichment analysis of negatively regulated genes highlighted a significant reduction in pathways related to synaptic transmission, neurotransmitter release, and synaptic vesicle recycling (Figure 5C). These results suggest that TSA modulates synaptic homeostasis, potentially through compensatory mechanisms that alter excitatory signaling and synaptic plasticity. The observed decrease in GRIA2, SYT1, and SYN1 expression further supports this hypothesis.

To assess the conservation of TSA‐induced transcriptional changes across species and cellular contexts, we performed a comparative analysis of TSA‐responsive genes in mouse primary hippocampal neurons^31^ and human iPSC‐derived cortical neurons. Using ortholog mapping, we identified 9333 genes with 1:1 mouse‐human orthology detected in both datasets (File S20). Among these, we identified 3468 genes significantly upregulated in mouse and 2876 genes significantly upregulated in human iPSC neurons in response to TSA treatment (*p* < 0.05, log_2_FC > 0.58). We identified 262 genes with conserved TSA responses showing the same direction of regulation in both species (219 upregulated, 43 downregulated), representing 6.3% of mouse and 7.6% of human TSA‐upregulated genes (Figure S6A).

Correlation analysis across all detected orthologs revealed substantial divergence (Pearson *r* = −0.651), indicating that TSA induces largely distinct transcriptional programs in hippocampal versus cortical neurons (Figure S6B). However, when restricted to the 262 genes with conserved responses, the correlation increased dramatically to *r* = 0.779, demonstrating strong consistency in both magnitude and direction of TSA effects for this conserved gene set. This pattern indicates that, while hippocampal and cortical neurons exhibit substantial cell‐type‐specific TSA responses, a core subset of genes responds consistently across neuronal subtypes and species.

DISC1 showed significant TSA responsiveness in both mouse primary hippocampal neurons (log_2_FC = 0.675, *p* = 0.032) and human iPSC‐derived cortical neurons (log2FC = 0.65, *p* = 0.007), confirming that DISC1 is epigenetically sensitive to HDAC inhibition across both species and cellular contexts (File S20). The consistent upregulation in both systems demonstrates that TSA modulates DISC1 expression regardless of whether neurons are primary or iPSC‐derived, supporting the translational relevance of our iPSC model for studying DISC1 regulation. Among other conserved upregulated genes, we identified multiple factors involved in neuronal development and synaptic function, including ion channels (KCNQ5, SCN9A), transcription factors (TCF4, ZEB2), and signaling molecules (IL16, MAS1, CRYM, CAPN3) (Figure S6C). Conserved downregulated genes included SLC5A7 (choline transporter critical for acetylcholine synthesis), UNCX (homeobox transcription factor), and ONECUT1 (neuronal transcription factor).

At the biological process level, conserved genes showed enrichment in regulation of membrane potential, endothelium development, telencephalon development, and potassium ion transport, providing functional validation of conserved TSA‐responsive pathways related to neuronal excitability and development (Figure S6D). Gene ontology enrichment analysis of the 262 conserved TSA‐responsive genes revealed significant enrichment at the molecular function level, including potassium ion transmembrane transporter activity, monoatomic ion channel activity, integrin binding, and metal ion transmembrane transporter activity (Figure S6E).

The substantial divergence in overall TSA responses between mouse hippocampal and human cortical neurons (*r* = −0.651) reflects fundamental biological differences between these neuronal populations. Hippocampal and cortical neurons possess distinct transcriptional programs, functional roles in neural circuits, and chromatin landscapes that shape their responses to HDAC inhibition. Several genes illustrated this cell‐type specificity: GAP43, a marker of axonal growth and regeneration, was strongly upregulated in mouse hippocampal neurons (log_2_FC = 2.476, *p* < 0.001) but downregulated in human cortical neurons (log_2_FC = −3.123, *p* < 0.001). Similarly, GRIA2, an AMPA receptor subunit critical for synaptic plasticity, showed opposite regulation (mouse: log_2_FC = 2.109, human: log_2_FC = −0.922). These divergent patterns likely reflect the distinct developmental programs, synaptic properties, and functional specialization of hippocampal versus cortical neurons rather than artifacts of iPSC reprogramming.

Importantly, the high correlation among the 262 conserved genes (*r* = 0.779) demonstrates that a core transcriptional program induced by HDAC inhibition is preserved across neuronal subtypes and species. The fact that DISC1 falls within this conserved set, showing consistent upregulation with similar effect sizes (log_2_FC ≈ 0.7 in both systems), validates that DISC1 epigenetic regulation by TSA represents a fundamental mechanism rather than a cell‐type‐specific artifact. The conservation of 262 genes despite the overall transcriptional divergence between hippocampal and cortical neurons demonstrates that iPSC reprogramming does not abolish TSA responsiveness, but rather that the chromatin regulatory machinery targeted by HDAC inhibitors is functionally re‐established during iPSC differentiation in a manner that preserves core regulatory mechanisms while allowing cell‐type‐specific responses.

### 3.7 Transcriptional profiling reveals distinct AD‐associated microglial subtypes with differential gene expression

To investigate how microglial heterogeneity may contribute to AD, we analyzed transcriptional profiles of myeloid cells from the Lee et al.^18^ dataset. Differential expression analysis was performed for each microglial subtype to identify gene signatures specific to homeostatic, disease‐associated, inflammatory, proliferative, and perivascular states (File S21, S22). This analysis revealed distinct transcriptional patterns that were reproducible across both fresh and frozen tissue preparations, highlighting the robustness of the identified subtype markers. Homeostatic microglia were enriched for canonical genes such as P2RY12 and PICALM, while disease‐associated microglia upregulated genes involved in phagocytosis and lipid metabolism, including GPNMB and TREM2. In contrast, pro‐inflammatory microglia exhibited elevated expression of AIF1, CCL3, and other chemokines, consistent with a reactive immune phenotype.

Subtype‐specific differential expression patterns were further validated by comparing AUROC scores and percentage expression shifts across conditions, revealing several genes consistently enriched or depleted in distinct microglial populations (Files S21, S22). This analysis, broken down by specific microglial subtypes, highlights the distinct gene expression patterns of microglial cell states in AD and underscores the value of integrating microglial and neuronal data to uncover shared molecular targets with therapeutic potential. The reproducibility of the expression trends across fresh and frozen tissue further strengthens confidence in the robustness of the microglial subtype annotations and their downstream interpretations.

### 3.8 Convergence on DISC1 as a key molecular target

To explore shared molecular mechanisms across our experimental datasets, we performed an integrative analysis comparing DEGs from TSA‐treated neurons, microglial subtypes in the Lee et al.^18^ dataset, and AD‐associated neurons identified via DEGAS analysis. We identified overlap between these three distinct analyses, highlighting genes that are recurrently upregulated across multiple experimental conditions (Figure 5D, File S23). DISC1, notably present in the intersection of all three datasets (TSA, Lee et al., and DEGAS), plays critical roles in neurodevelopment and synaptic function and has previously been linked to both psychiatric disorders and neurodegeneration.

Examination of DISC1 expression using HPA and GTEx databases revealed significant enrichment in several brain regions vulnerable to AD pathology (Figure S7). DISC1 showed the highest expression in the parahippocampal gyrus, superior temporal gyrus, and frontal pole, which are regions critically involved in memory processing and early AD pathology (Figure S7). At the cellular level, DISC1 exhibited pronounced expression in neuronal populations, with lower expression in glial cells, suggesting cell type‐specific functions relevant to AD pathogenesis (Figure S7).

Analysis of DISC1 expression across diverse microglial subtypes in the Lee et al.^18^ dataset revealed distinct patterns that suggest a nuanced role in AD pathology. We evaluated DISC1 expression in microglial populations obtained from both fresh and frozen brain tissue samples, uncovering consistent expression signatures that persisted across sample preparation methods. DISC1 showed significant differential expression across microglial subtypes with clear patterns suggesting distinct roles in AD pathophysiology (Table 1).

**TABLE 1:** DISC1 expression patterns across microglial subtypes.

Tissue	Subclass	Subtype	log_2_FC	AUROC	*p*‐value
Fresh	ADAM	GPNMB	0.664	0.554	< 0.001
Fresh	Adaptive	AIF1	−2.057	0.303	< 0.001
Fresh	Adaptive	CCL3	−1.417	0.355	< 0.001
Fresh	Adaptive	HIF1A	−0.630	0.412	< 0.001
Fresh	Adaptive	HIST	−0.109	0.483	< 0.001
Fresh	Adaptive	HSPA1A	0.228	0.504	< 0.001
Fresh	Adaptive	IFI44L	−0.127	0.483	< 0.001
Fresh	Adaptive	TMEM163	0.633	0.559	< 0.001
Fresh	Homeostatic	FRMD4A	0.538	0.548	< 0.001
Fresh	Homeostatic	PICALM	1.056	0.623	< 0.001
Fresh	PVM	CD163	1.092	0.620	< 0.001
Fresh	Proliferative	MKI67	−0.487	0.433	< 0.001
Fresh	exAM	ERN1	−0.442	0.455	< 0.001
Frozen	ADAM	GPNMB	0.487	0.573	< 0.001
Frozen	Adaptive	CCL3	−1.186	0.355	< 0.001
Frozen	Adaptive	HIF1A	−0.720	0.400	< 0.001
Frozen	Adaptive	HIST	−0.285	0.464	< 0.001
Frozen	Adaptive	HSPA1A	−0.623	0.429	< 0.001
Frozen	Adaptive	TMEM163	0.174	0.529	< 0.001
Frozen	Homeostatic	FRMD4A	−0.032	0.488	< 0.001
Frozen	Homeostatic	PICALM	0.174	0.524	< 0.001
Frozen	PVM	CD163	0.244	0.526	< 0.001
Frozen	Proliferative	MKI67	−0.103	0.470	< 0.001
Frozen	exAM	ERN1	−0.819	0.441	< 0.001

*Note*: Differential expression analysis of DISC1 across distinct microglial subtypes, adapted from the Lee et al. dataset comparing fresh and frozen tissue preparations. Subclass categories: ADAM (disease‐associated microglia), Adaptive (stress‐responsive microglia), Homeostatic (resting microglia), PVM, Proliferative (dividing microglia), exAM. Subtype markers represent characteristic genes defining each microglial population. log2FC indicates log2 fold change in DISC1 expression relative to reference population. AUROC values indicate the discriminative power of DISC1 expression for each subtype. All comparisons showed statistical significance with *p* < 0.001.

Abbreviations: AUROC, area under receiver operating characteristic curve; DISC1, Disrupted‐In‐Schizophrenia 1; exAM, excessively activated microglia; log2FC, log2 fold change; PVM, perivascular macrophages.

Three key microglial populations consistently upregulated DISC1 across sample types. Disease‐associated microglia expressing GPNMB showed notable upregulation in both fresh (log2FC = 0.664) and frozen (log2FC = 0.487) samples (Table 1), suggesting the involvement of DISC1 in protective phagocytic functions associated with this AD‐enriched microglial population. Homeostatic microglia marked by PICALM expression demonstrated even stronger DISC1 upregulation, particularly in fresh tissues (log2FC = 1.056), with maintained expression in frozen samples (log2FC = 0.174) (Table 1). This association with PICALM, a known genetic risk factor for AD, may offer insights into the role of DISC1 in disease mechanisms. Perivascular macrophages identified by CD163 expression also exhibited consistent DISC1 upregulation across sample preparations (fresh: log2FC = 1.092; frozen: log2FC = 0.244) (Table 1), pointing to potential functions in blood–brain barrier maintenance and neuroimmune interactions at the vascular interface.

However, DISC1 was consistently downregulated across microglial subtypes associated with inflammation and proliferation. Pro‐inflammatory microglia expressing AIF1 (also known as IBA1) showed pronounced DISC1 downregulation (log2FC = −2.057 in fresh samples) (Table 1), suggesting DISC1 expression becomes suppressed during pro‐inflammatory microglial activation. Similarly, CCL3‐expressing microglia exhibited substantial DISC1 downregulation in both sample types (fresh: log2FC = −1.417; frozen: log2FC = −1.186) (Table 1), indicating a negative relationship between DISC1 and inflammatory chemokine production. Hypoxia‐responsive microglia expressing HIF1A also showed consistent DISC1 downregulation across preparations (fresh: log2FC = −0.630; frozen: log2FC = −0.720) (Table 1), further supporting an inverse relationship between DISC1 and inflammatory states. This pattern extended to proliferating microglia marked by MKI67 and excessively activated microglia expressing ERN1, both showing consistent DISC1 downregulation across sample types.

Some microglial subtypes exhibited variability in DISC1 expression patterns related to tissue processing. Notably, adaptive microglia expressing HSPA1A showed contrasting DISC1 regulation: upregulation in fresh tissue but downregulation in frozen samples (Table 1). Similarly, homeostatic FRMD4A‐positive microglia displayed moderate DISC1 upregulation in fresh tissue but nearly neutral expression in frozen preparations (Table 1). These discrepancies likely reflect technical factors in sample processing or inherent biological variability in these specific activation states.

Transcriptomic analyses across defined microglial states indicate that DISC1 is preferentially expressed in homeostatic, neuroprotective microglial subtypes, implicating a role in maintaining CNS immune surveillance and neuronal integrity (Table 1). In contrast, DISC1 expression is markedly downregulated upon transition to disease‐associated activation states (Table 1), suggesting its suppression is a feature of the microglial response to pathological stimuli, providing potential insights for therapeutic approaches targeting microglial function.

### 3.9 TSA prevents Aβ‐induced neurotoxicity in human iPSC‐derived cortical neurons through DISC1 upregulation

The results of the viability assay revealed that TSA pre‐treatment significantly improved cell viability when neurons were exposed to Aβ oligomers. Specifically, TSA at 0.2 µM (60 ng/mL) demonstrated optimal protection, maintaining higher viability percentages compared to 0.066 µM (20 ng/mL) and 0.33 µM (100 ng/mL) (Figure 5E). Values represent mean plus or minus standard error of the mean (SEM) viability expressed as a percentage relative to untreated control wells, as described in the Methods section. The results demonstrate that increasing concentrations of Aβ oligomers reduced neuronal viability, whereas TSA pretreatment preserved viability across the tested range. Neurons pre‐treated with TSA showed a higher number of synaptic clusters compared to non‐treated neurons (Figure 5F). Furthermore, the colocalization studies indicated that pre‐treatment of TSA mitigated the synaptic toxicity of Aβ oligomers (Figure 5G). These results indicate that TSA provides a protective effect against Aβ‐induced synaptic toxicity and helps maintain synaptic integrity.

**FIGURE 6:**
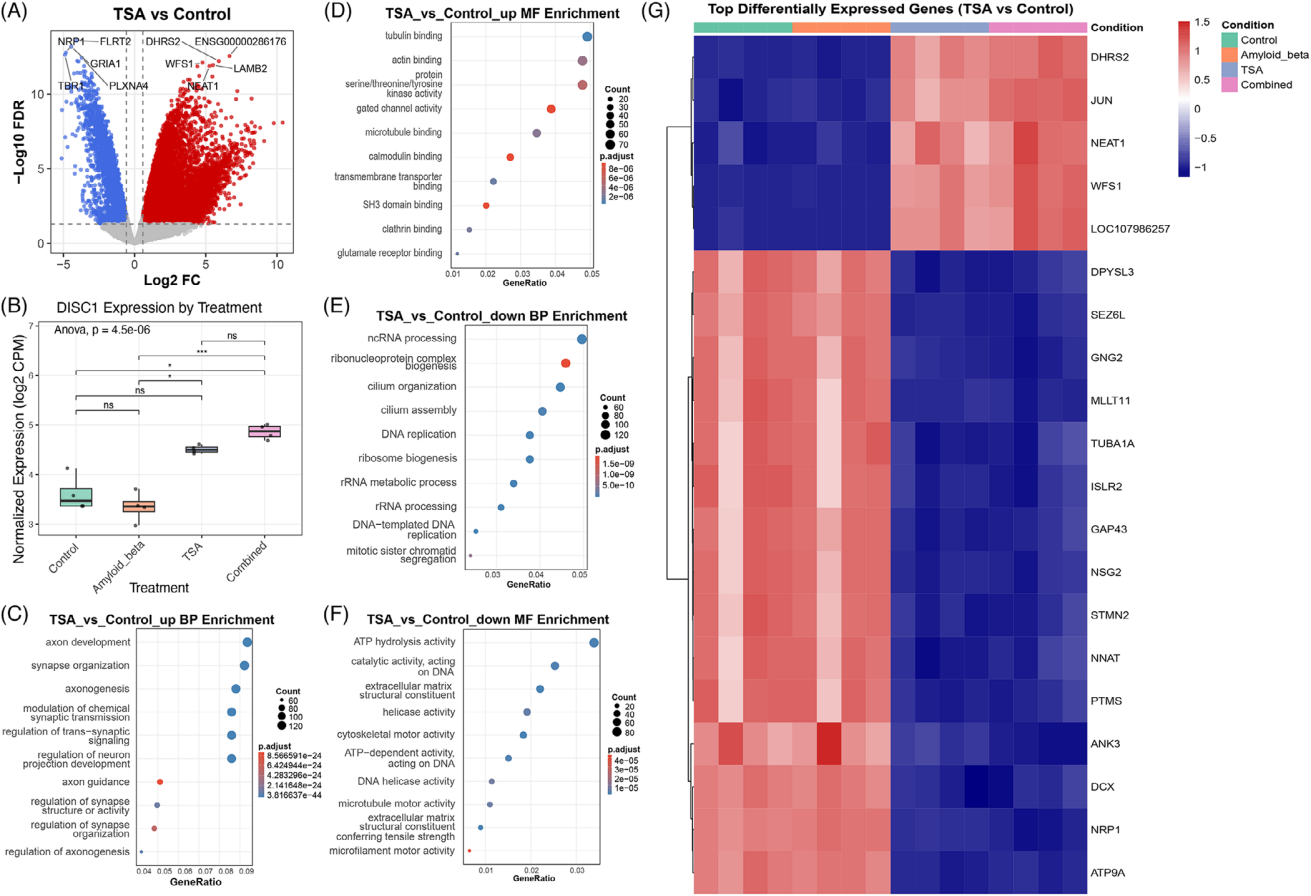
**Transcriptomic analysis of TSA effects on iPSC‐derived cortical neurons reveals distinct gene expression patterns**. (A) Volcano plot displaying DEGs between control and TSA‐treated human iPSC‐derived cortical neurons. Significantly upregulated genes are shown in red and downregulated genes in blue (adjusted *p*‐value < 0.05, |log_2_FC| > 0.58). (B) Box plots showing DISC1 expression levels (normalized log2CPM) across four treatment conditions: Control, Amyloid_beta (Aβ alone), TSA (TSA alone), and Combined (TSA + Aβ). Statistical comparisons are indicated with brackets and significance levels. (C) GO Biological Process enrichment analysis for genes upregulated by TSA treatment. Dot size represents gene count, and color indicates ‐log_10_(FDR). (D) GO Biological Process enrichment analysis for genes downregulated by TSA treatment. (E) GO Molecular Function enrichment for genes upregulated by TSA treatment. (F) GO Molecular Function enrichment for genes downregulated by TSA treatment. (G) Heatmap showing expression patterns of top differentially expressed genes across all samples. Samples are grouped by treatment condition (Control, Amyloid_beta, TSA, Combined) with color‐coded bars at the top. Gene expression is displayed as normalized *z*‐scores, with red indicating high expression and blue indicating low expression. Aβ, β‐amyloid; DEG, differentially expressed genes; DISC1, Disrupted‐In‐Schizophrenia 1; FDR, false discovery rate; GO, gene ontology; iPSC, induced pluripotent stem cell; TSA, trichostatin‐A.

Our transcriptomic analysis of iPSC‐derived cortical neurons revealed significant insights into how TSA reshapes the neuronal transcriptome in response to Aβ exposure. Differential expression analysis between the four treatment groups (control, TSA‐only, Aβ‐only, and combined TSA+Aβ) uncovered distinct gene expression patterns with important implications for AD pathology. There is a substantial transcriptional response to TSA treatment, with 2773 genes significantly upregulated and 3,167 genes downregulated compared to control conditions (Figure 6A).

DISC1, known for its roles in neurodevelopment and synaptic plasticity, showed significant differential expression across treatment conditions (analysis of variance [ANOVA] p = 4.5×10^−^^6^). Aβ alone did not significantly alter DISC1 expression compared to control (log_2_FC = −0.26, 0.84‐fold, adjusted *p* = 1.00), indicating that Aβ pathology does not directly affect DISC1 transcription in this model (Figure 6B).

In contrast, the combined TSA+Aβ treatment significantly upregulated DISC1 expression compared to control (log_2_FC = 1.25, 2.39‐fold increase, adjusted *p* = 0.018), demonstrating a 138.6% increase over baseline. More strikingly, the combined treatment elevated DISC1 by 2.86‐fold compared to Aβ treatment alone (log2FC = 1.51, 185.6% increase, adjusted *p* = 0.003), representing the strongest effect observed among all pairwise comparisons (Figure 6B). TSA alone also significantly increased DISC1 expression relative to Aβ‐treated cells (log_2_FC = 1.16, 2.23‐fold increase, adjusted *p* = 0.019), further supporting TSA's robust capacity to upregulate DISC1 and counteract Aβ‐associated transcriptional changes (Figure 6B).

When comparing TSA alone to control, a substantial 86.4% increase in DISC1 expression was observed (log_2_FC = 0.90, 1.86‐fold), though this did not reach statistical significance after Bonferroni correction for multiple comparisons (adjusted *p* = 0.079) (Figure 6B). Similarly, no significant difference was detected between TSA alone and the combined treatment (log_2_FC = 0.36, 1.28‐fold, adjusted *p* = 0.064), suggesting that TSA's upregulatory effect on DISC1 is preserved in the presence of Aβ pathology (Figure 6B).

To understand the broader biological implications of TSA treatment, we performed GO enrichment analysis on both downregulated and upregulated gene sets. Genes downregulated by TSA treatment showed significant enrichment in biological processes related to ncRNA processing, cilium organization, ribosome biogenesis, and DNA replication (Figure 6C). At the molecular function level, downregulated genes were enriched in catalytic activities acting on RNA and DNA, ATP hydrolysis, and various transferase activities (Figure 6D). These findings suggest TSA treatment may suppress certain cellular metabolic processes while upregulating neuron‐specific functions.

In contrast, genes upregulated by TSA treatment showed striking enrichment in biological processes critical for neuronal function and connectivity, including synapse organization, modulation of chemical synaptic transmission, regulation of trans‐synaptic signaling, and axonogenesis (Figure 6E). These pathways are directly relevant to maintaining and restoring neuronal communication that is disrupted in AD. At the molecular function level, upregulated genes were significantly enriched in tubulin binding, actin binding, protein kinase activity, and guanosine triphosphate (GTP)‐related functions (Figure 6F), suggesting enhanced cytoskeletal dynamics and signaling capacity. Notably, we also observed enrichment in amyloid‐beta binding among upregulated genes, indicating potential mechanisms for mitigating Aβ toxicity.

Hierarchical clustering of the top differentially expressed genes across all treatment conditions revealed distinct expression patterns that clearly separated TSA‐treated samples from untreated samples (Figure 6G). TSA‐treated neurons (both TSA‐only and TSA+Aβ conditions) showed similar transcriptional profiles characterized by upregulation of genes involved in neuroplasticity (including JUN, NEAT1, and WFS1) and downregulation of genes such as ANK3, TUBA1A, and GAP43 (Figure 6G). This clustering pattern further supports the observation that TSA exerts dominant transcriptional effects that persist even in the presence of Aβ, providing a potential molecular basis for its neuroprotective properties.

**FIGURE 7:**
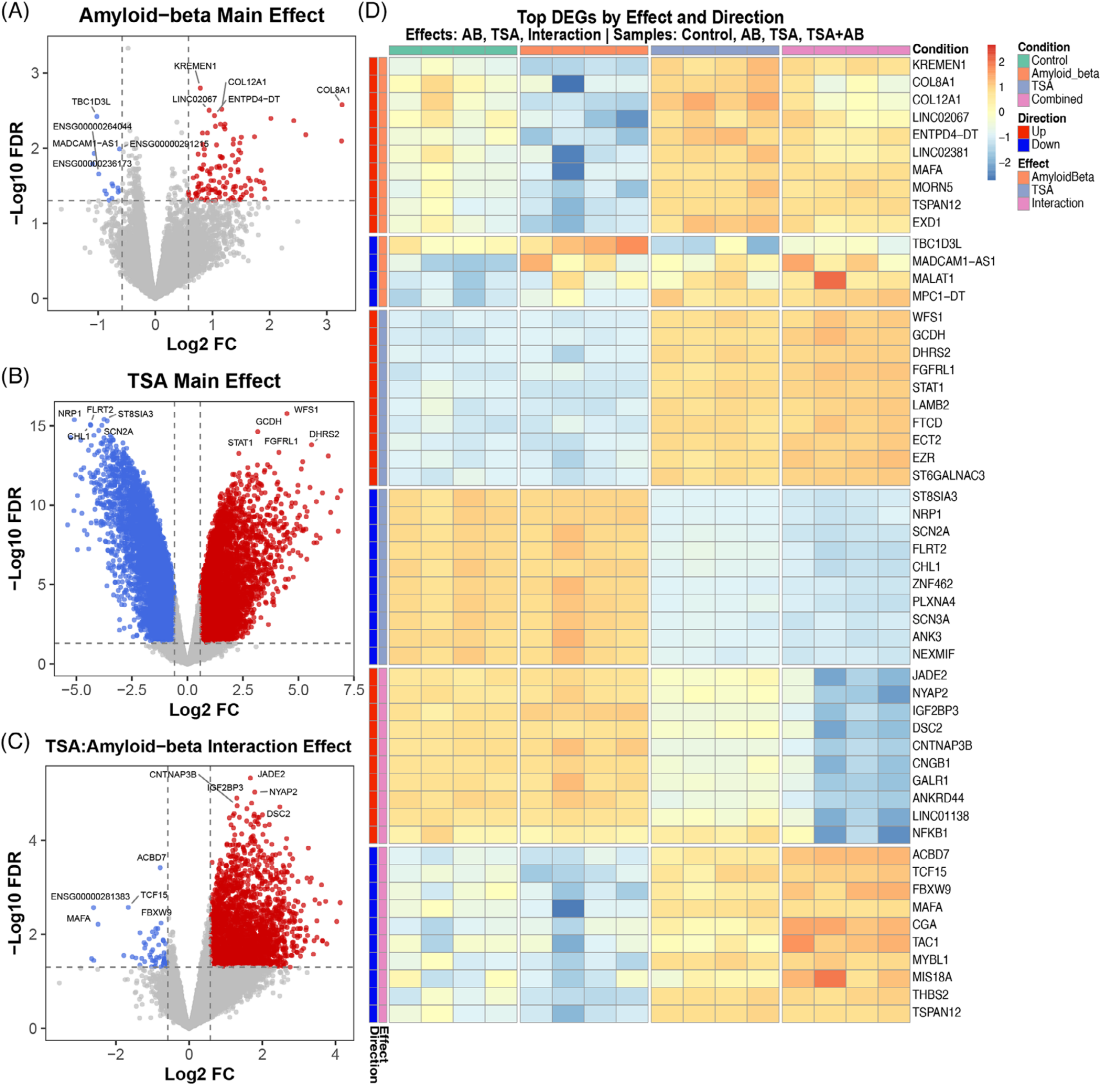
Differential gene expression analysis of TSA and amyloid‐beta treatment in neural cells. (A) Volcano plot showing the main effect of Aβ treatment on gene expression (Amyloid‐beta Main Effect). Significantly downregulated genes are shown in blue and upregulated genes in red (adjusted *p*‐value < 0.05, |log_2_FC| > 0.58). (B) Volcano plot displaying the main effect of TSA treatment on gene expression (TSA Main Effect). Significantly downregulated genes are shown in blue and upregulated genes in red. (C) Volcano plot illustrating the interaction effect between TSA and Aβ treatments (TSA:Amyloid‐beta Interaction Effect). Significantly downregulated genes are shown in blue and upregulated genes in red. (D) Heatmap displaying expression patterns of top differentially expressed genes across experimental conditions, clustered by effect type. Samples are organized by treatment condition: Control, AB (Aβ), TSA, and TSA+AB (Combined). Left sidebar color bars indicate Condition (Control, Amyloid_beta, TSA, Combined). Right sidebar color bars indicate Direction (Up, Down) and Effect (TSA, Amyloid‐beta, Interaction). Expression values are shown as normalized *z*‐scores with yellow indicating high expression and blue indicating low expression. Aβ, β‐amyloid; TSA, trichostatin‐A.

Our comprehensive gene expression analysis comparing the effects of TSA and Aβ treatments on iPSC‐derived neural cells revealed distinct transcriptional signatures. The amyloid‐beta main effect showed a relatively moderate transcriptional response with several significantly upregulated genes and downregulated genes (Figure 7A). In contrast, the TSA main effect produced a substantially more robust transcriptional response, with numerous genes showing significant differential expression, particularly those that are upregulated (Figure 7B).

The interactions between TSA and Aβ treatment revealed an extensive set of genes whose expression was specifically modulated by the combination of both, indicating additive, synergistic, or opposing regulatory mechanisms beyond the individual effects of either treatment alone (Figure 7C). Of particular interest, we observed that the interaction effect produced a pattern of differential expression distinct from either individual treatment, suggesting that TSA may remodel the transcriptional landscape in response to Aβ exposure.

Hierarchical clustering of top DEGs across all experimental conditions further illustrated the complex transcriptional patterns governing cellular responses to TSA and amyloid‐beta (Figure 7D). Distinct gene expression modules were revealed that were systematically regulated across treatment conditions. Gene expression patterns in TSA‐treated samples showed notable differences from control samples, with several genes displaying opposite regulation patterns between TSA and amyloid‐beta treatments (Figure 7D). These results provided visual confirmation that TSA treatment could counteract some of the amyloid‐beta‐induced transcriptional changes, as evidenced by the similarity in expression patterns between the TSA‐only and TSA‐plus‐amyloid‐beta conditions compared to control and amyloid‐beta‐only conditions for multiple genes (Figure 7D). Several Ensembl IDs in this analysis could not be mapped to conventional gene names as they represent genomic regions that have not been sufficiently characterized or annotated in current databases (Figure S8).

Among the DEGs identified in our analysis, several targets with established roles in neurodegenerative pathology exhibited clear reversal patterns, where TSA treatment counteracted Aβ‐induced changes (Figure 7D). ANK3 (ankyrin‐3), which encodes ankyrin‐G protein critical for neuronal structure and synaptic function,^42^ displayed elevated expression under Aβ conditions relative to control, while showing marked downregulation in TSA‐treated conditions. Ankyrin‐G functions as a scaffolding protein essential for axon initial segment organization and synaptic transmission, with genetic variants linked to multiple neuropsychiatric disorders.^43^ The observed expression pattern suggests that TSA treatment may modulate ankyrin‐G activity that becomes dysregulated under Aβ stress, potentially restoring normal cytoskeletal organization.

GCDH (glutaryl‐CoA dehydrogenase), a mitochondrial enzyme crucial for lysine and tryptophan catabolism, exhibited suppressed expression in Aβ conditions but was substantially upregulated following TSA treatment. Glutamate dehydrogenase dysfunction has been implicated in neurodegenerative diseases, including AD, where impaired glutamate metabolism contributes to synaptic dysfunction.^44^ The restoration of GCDH expression by TSA suggests activation of mitochondrial metabolic programs that are compromised in amyloid pathology.

TAC1 (tachykinin precursor 1), which encodes substance P and other neuropeptides involved in synaptic transmission and neuronal signaling, demonstrated reduced expression under Aβ conditions but showed partial restoration following TSA treatment. TAC1 has been identified as a key hub gene in AD, with significant downregulation observed in the frontal cortex of AD patients and in transgenic mouse models.^45^ The gene is functionally connected to synaptic transmission and neuronal development pathways critical for cognitive function. The observed expression pattern suggests that TSA treatment may counteract Aβ‐induced suppression of TAC1, potentially restoring neuropeptide signaling networks that become compromised in neurodegenerative contexts.

LAMB2 (laminin beta‐2), an extracellular matrix component essential for basement membrane integrity, showed reduced expression under Aβ conditions but was markedly upregulated following TSA treatment. Laminin‐β2 is critical for blood–brain barrier function and has direct interactions with Aβ peptides in Alzheimer's pathology.^46^ The pronounced reversal in LAMB2 expression suggests that TSA modulates extracellular matrix remodeling pathways that become dysregulated in neurodegenerative contexts, potentially restoring vascular integrity.

These findings demonstrate that TSA treatment can reverse specific Aβ‐induced transcriptional changes across multiple cellular pathways, including cytoskeletal organization, mitochondrial metabolism, and extracellular matrix homeostasis. This pattern of molecular reversal supports the therapeutic potential of HDAC inhibitors in mitigating transcriptional dysfunction associated with AD pathogenesis.

## 4 DISCUSSION

This study implemented an integrative computational and experimental approach to identify therapeutic candidates for AD, revealing DISC1 as a convergent molecular target consistently upregulated across TSA‐treated neurons, AD‐associated neuronal subpopulations, and homeostatic microglial subtypes. Analysis of three independent single‐cell datasets spanning entorhinal cortex (Grubman et al.,^13^ 13,214 nuclei) and prefrontal cortex (Mathys et al.,^14^ 80,660 profiles; Green et al.,^15^ 1,173,122 nuclei) identified TSA as one of only three compounds achieving statistical significance (FDR < 0.1) across all datasets, with multiple structurally distinct HDAC inhibitors, including SB‐939 (pracinostat), vorinostat [SAHA], belinostat, mocetinostat, and entinostat, emerging independently among top candidates. This convergence on HDAC inhibition, combined with functional validation in human iPSC‐derived neurons demonstrating neuroprotection against Aβ‐induced toxicity, positions DISC1 enhancement as a promising disease‐modifying strategy.

The three‐way convergence on DISC1 is particularly compelling because these datasets represent fundamentally different experimental contexts: pharmacological perturbation, disease‐associated cellular states, and computationally defined vulnerable neuronal subpopulations. TSA significantly upregulated DISC1 in both mouse hippocampal neurons (log_2_FC = 0.675, *p* = 0.032) and human iPSC‐derived cortical neurons (log_2_FC = 0.65, *p* = 0.007), with this upregulation persisting in the presence of Aβ oligomers (log2FC = 0.54, *p* = 0.021). The maintained DISC1 expression under combined treatment (56% increase vs. Aβ alone, *p* = 0.018) suggests DISC1 induction represents a primary neuroprotective mechanism rather than a secondary consequence of reduced cellular stress. Microglial analysis revealed DISC1 upregulation in homeostatic (PICALM‐positive, log2FC = 1.056), disease‐associated phagocytic (GPNMB‐positive, log2FC = 0.664), and perivascular (CD163‐positive, log2FC = 1.092) populations, while being markedly downregulated in pro‐inflammatory (AIF1‐positive, log2FC = −2.057) and chemokine‐producing (CCL3‐positive, log2FC = −1.417) microglia. This dual targeting of neuronal and microglial compartments distinguishes TSA from single‐compartment therapeutic candidates.

DISC1 functions as a multifunctional scaffolding protein coordinating cellular processes disrupted in AD through three interconnected pathways supported by substantial prior evidence. First, DISC1 physically interacts with glycogen synthase kinase 3 beta (GSK3β), a key kinase responsible for pathological tau hyperphosphorylation in AD.^47^ The interaction occurs through DISC1's C‐terminal proline‐rich region, which competes with GSK3β’s binding to scaffolding proteins like AXIN, thereby disrupting formation of active GSK3β signaling complexes.^48^ This direct inhibition prevents GSK3β from phosphorylating tau at multiple AD‐relevant epitopes, including Ser396, Ser404, and Thr231.^49^ Studies in diabetic encephalopathy models demonstrated that DISC1 overexpression significantly reduced GSK3β activity and prevented tau hyperphosphorylation at these pathogenic sites, while DISC1 knockdown resulted in elevated GSK3β activity and increased tau phosphorylation.^49^ This mechanism is particularly relevant because GSK3β hyperactivity occurs early in AD pathogenesis, before significant amyloid plaque formation.^50^ TSA‐mediated DISC1 upregulation would therefore increase the cellular pool of endogenous GSK3β inhibitors, potentially interrupting the critical pathway linking Aβ toxicity to tau pathology.

Second, DISC1 regulates mitochondrial dynamics through interaction with mitochondrial Rho GTPase 1 (MIRO1) and motor protein complexes.^51^, ^52^ DISC1 localizes to the outer mitochondrial membrane, where it forms complexes with MIRO1, TRAK1/2 trafficking proteins, and dynein/kinesin motors to facilitate bidirectional mitochondrial transport along microtubules.^52^ This DISC1‐MIRO1 complex is critical for activity‐dependent mitochondrial positioning at synapses, where energy demands fluctuate based on synaptic activity.^53^ MIRO1 contains EF‐hand calcium‐binding domains that enable the DISC1‐MIRO1 complex to modulate calcium transfer from endoplasmic reticulum to mitochondria during elevated cytosolic calcium, preventing mitochondrial calcium overload that triggers apoptosis.^52^, ^54^ DISC1 deficiency results in mitochondrial calcium dysregulation, increased oxidative stress, and enhanced vulnerability to excitotoxic insults.^52^ In AD, mitochondria become dysfunctional, exhibit reduced motility, and accumulate in dystrophic neurites, contributing to synaptic failure.^55^, ^56^ By upregulating DISC1, TSA would enhance mitochondrial transport capacity and improve positioning at synapses where energy demands are highest, counteracting the mitochondrial dysfunction characteristic of AD and potentially explaining the synaptic preservation we observed in TSA‐treated neurons exposed to Aβ oligomers.

Third, DISC1 modulates cyclic AMP signaling through physical interaction with phosphodiesterase 4 (PDE4), an enzyme that degrades Camp.^57^ This interaction localizes PDE4 activity to specific subcellular compartments, creating cAMP microdomains that enable precise regulation of downstream effectors, including protein kinase A (PKA) and exchange protein activated by cAMP (EPAC).^57^, ^58^ Through this mechanism, DISC1 influences AMPA receptor trafficking, dendritic spine morphology, and presynaptic vesicle release probability.^59^, ^60^ Disruption of DISC1‐PDE4 interactions impairs synaptic plasticity and cognitive function in rodent models,^61^, ^62^ while interventions enhancing DISC1 expression improve synaptic function and memory performance.^63^ In AD, Aβ oligomers directly interfere with long‐term potentiation and cause rapid dendritic spine loss.^64^, ^65^ TSA‐mediated DISC1 upregulation could restore cAMP signaling dynamics, preserving synaptic plasticity mechanisms required for cognitive function, consistent with our observation of maintained PSD95‐Syn1 colocalization in TSA‐treated neurons.

These three DISC1‐regulated pathways form an integrated network rather than independent mechanisms. GSK3β activity influences mitochondrial function through phosphorylation of mitochondrial proteins,^66^ while mitochondrial ATP production is required to maintain cAMP gradients and support energy‐intensive synaptic vesicle recycling.^67^ In AD, Aβ oligomers simultaneously activate GSK3β,^68^ impair mitochondrial transport,^69^ and suppress cAMP signaling,^65^ creating cascading failures culminating in synaptic dysfunction. By upregulating DISC1, TSA addresses multiple points of vulnerability simultaneously, providing systems‐level neuroprotection potentially more effective than single‐target interventions. This multi‐pathway modulation aligns with growing recognition that successful AD therapeutics will likely require addressing multiple pathogenic mechanisms.^70^, ^71^

The median‐split cell prioritization analysis revealed a notable directional pattern across brain regions. In the Grubman et al.^13^ entorhinal cortex dataset, where AD pathology originates, and neurons face early vulnerability, HDAC inhibitors (vorinostat #1, TSA #2, belinostat #9) emerged in mimic queries identifying compounds reproducing the disease signature. Conversely, in the Mathys et al.^14^ and Green et al.^15^ prefrontal cortex datasets representing later‐affected regions, HDAC inhibitors achieved top rankings in reverse queries identifying compounds opposing the disease signature (TSA #1‐2 in Mathys inhibitory neurons; vorinostat #2‐3 across multiple populations). This pattern may reflect stage‐dependent mechanisms: in early‐vulnerable entorhinal neurons approaching cell death, HDAC dysregulation may be integral to the pathological cascade, whereas in later‐affected prefrontal neurons mounting compensatory responses, HDAC inhibition may restore homeostatic gene expression. Alternatively, this may reflect Simpson's paradox, regional chromatin landscape differences, or differential HDAC isoform expression. Despite directional differences, HDAC inhibitors emerge as convergent therapeutic candidates across all six neuronal analyses, suggesting benefits across multiple disease stages through complementary mechanisms.

The cross‐species conservation of DISC1 responses validates translational relevance. While overall TSA responses diverged between mouse hippocampal and human cortical neurons (*r* = −0.651), reflecting genuine biological differences between functionally distinct neuronal subtypes, 262 genes showed conserved responses with strong positive correlation (*r* = 0.779). DISC1's presence in this conserved set with similar effect sizes (log_2_FC ≈ 0.7) demonstrates that DISC1 epigenetic regulation represents a fundamental mechanism preserved across neuronal subtypes and species, occurring despite dramatic epigenetic resetting during iPSC reprogramming.

Translating these findings to clinical applications presents several challenges requiring systematic address in future studies. First, TSA and related HDAC inhibitors have limited blood‐brain barrier permeability and broad off‐target epigenetic effects, complicating safety and dosing optimization in vivo. Rational design of isoform‐selective HDAC inhibitors targeting specific isoforms responsible for DISC1 repression, or compounds specifically enhancing DISC1 function through stabilization of DISC1‐GSK3β or DISC1‐PDE4 interactions, may offer improved therapeutic windows. The therapeutic window for DISC1 modulation requires careful determination, as excessive DISC1 expression in some neurodevelopmental contexts has shown adverse effects.^72^ Second, while iPSC‐derived neurons capture early neurodegenerative features, they lack the multicellular aged microenvironment characteristic of human AD. Follow‐up validation in three‐dimensional organoid models and transgenic mice recapitulating neuroinflammation, vascular pathology, and chronic Aβ accumulation is essential to test DISC1‐dependent mechanisms under physiologically relevant conditions that better approximate human disease progression. Third, definitive causal validation requires gain‐ and loss‐of‐function experiments, including DISC1 knockdown studies to determine whether TSA's neuroprotective effects depend on DISC1 upregulation, and DISC1 overexpression studies to assess whether DISC1 enhancement alone is sufficient for neuroprotection in the absence of HDAC inhibition. Fourth, pharmacokinetic and pharmacodynamic translation remains critical. Effective modulation of DISC1‐regulated pathways must be demonstrated at clinically achievable drug concentrations with minimal systemic toxicity, requiring careful dose‐response studies in preclinical models followed by Phase I safety trials in humans. The identification of biomarkers reflecting DISC1 pathway engagement, such as CSF or plasma measures of GSK3β activity, phosphorylated tau species, or metabolites reflecting mitochondrial function, would enable pharmacodynamic monitoring in early clinical trials.

Despite these challenges, our work establishes DISC1 as a convergent therapeutic target through integrative analysis spanning computational drug repurposing, spatial transcriptomics, cross‐species validation, and functional testing in human neurons. The identification of TSA as a neuroprotective agent operating through DISC1 upregulation in both neurons and microglia provides a mechanistic framework for understanding how epigenetic modulation can counteract multiple pathogenic processes. While TSA itself may have limited clinical utility due to broad epigenetic effects, the identification of DISC1 as a key mediator opens avenues for developing selective interventions enhancing DISC1 function with improved safety profiles. This study provides a methodological framework for future drug repurposing efforts and advances understanding of molecular mechanisms underlying cellular resilience against AD pathology.

## CONFLICT OF INTEREST STATEMENT

**Timothy I. Richardson**: Dr. Richardson reports: a pending US provisional patent application (no payments received); equity/stock options as Co‐Founder of Monument Biosciences; advisory role with equity/stock options at Enveda Biosciences; and consultant role with equity/stock options at Cadenza. No other financial or non‐financial interests to declare. **Kun Huang**: Dr. Huang reports equity/stock in Monument Biosciences. **Jie Zhang**: Dr. J. Zhang reports equity/stock in Monument Biosciences. **Cristian Lasagna‐Reeves**: Dr. Lasagna‐Reeves reports consulting for Monument Biosciences. Travis S. Johnson, Sean McCabe, Pengyue Zhang, Nur Jury‐Garfe, Steven Brooks, Caleb Beimfohr, Jiahui Liu, Chitra Sunil, Madeline Peyton: These coauthors have no conflicts to report.Author disclosures are available in the Supporting Information.

## CONSENT STATEMENT

All authors consent to publication.

## Supporting information



Supporting Information

Supporting Information

Supporting Information

## References

[1] Staff MC . Alzheimer's disease. Mayo Clinic. 2024.

[2] National Institue on Aging . What Is Alzheimer's Disease? 2021.

[3] Centers for Disease control and Prevention . Alzheimer's Disease and Related Dementias . 2020.

[4] National Institue on Aging . What Happens to the Brain in Alzheimer's Disease? 2017.

[5] Fakhoury M . Microglia and astrocytes in Alzheimer's disease: implications for therapy. Curr Neuropharmacol. 2018;16(5):508‐518. 28730967 10.2174/1570159X15666170720095240 PMC5997862

[6] Congressional Budget Office . Research and Development in the Pharmaceutical Industry . 2021.

[7] Pushpakom S , Iorio F , Eyers PA , et al. Drug repurposing: progress, challenges and recommendations. Nat Rev Drug Discov. 2019;18(1):41‐58. 30310233 10.1038/nrd.2018.168

[8] Fang J , Zhang P , Wang Q , et al. Artificial intelligence framework identifies candidate targets for drug repurposing in Alzheimer's disease. Alzheimers Res Ther. 2022;14(1):7. 35012639 10.1186/s13195-021-00951-z PMC8751379

[9] Lawlor B , Kennelly S , O'Dwyer S , et al. NILVAD protocol: a European multicentre double‐blind placebo‐controlled trial of nilvadipine in mild‐to‐moderate Alzheimer's disease. BMJ Open. 2014;4(10):e006364. 10.1136/bmjopen-2014-006364 PMC4194801 25300460

[10] Forloni G . Doxycycline: an essential tool for Alzheimer's disease. Biomed Pharmacother. 2025;188:118159. 40367557 10.1016/j.biopha.2025.118159 PMC12165865

[11] Cummings J , Zhou Y , Lee G , Zhong K , Fonseca J , Cheng F . Alzheimer's disease drug development pipeline: 2023. Alzheimers Dement Transl Res Clin Interv. 2023;9(2):e12385. 10.1002/trc2.12385 PMC10210334 37251912

[12] Rodriguez S , Hug C , Todorov P , et al. Machine learning identifies candidates for drug repurposing in Alzheimer's disease. Nat Commun. 2021;12(1):1033. 33589615 10.1038/s41467-021-21330-0 PMC7884393

[13] Grubman A , Chew G , Ouyang JF , et al. A single‐cell atlas of entorhinal cortex from individuals with Alzheimer's disease reveals cell‐type‐specific gene expression regulation. Nat Neurosci. 2019;22(12):2087‐2097. 31768052 10.1038/s41593-019-0539-4

[14] Mathys H , Davila‐Velderrain J , Peng Z , et al. Single‐cell transcriptomic analysis of Alzheimer's disease. Nature. 2019;570(7761):332‐337. 31042697 10.1038/s41586-019-1195-2 PMC6865822

[15] Green GS , Fujita M , Yang H , et al. Cellular communities reveal trajectories of brain ageing and Alzheimer's disease. Nature. 2024;633(8030):634‐645. 39198642 10.1038/s41586-024-07871-6 PMC11877878

[16] LINCS Consortium . L1000 Dataset ‐small molecule perturbagens ‐ LINCS Trans‐Center Project . 2014.

[17] Johnson TS , Yu CY , Huang Z , et al. Diagnostic evidence GAuge of single cells (DEGAS): a flexible deep transfer learning framework for prioritizing cells in relation to disease. Genome Med. 2022;14(1):e12385. 10.1186/s13073-022-01012-2 PMC8808996 35105355

[18] Lee D , Vicari JM , Porras C , et al., Plasticity of human microglia and brain perivascular macrophages in aging and Alzheimer's disease. medRxiv. Preprint. 2024; doi:10.1101/2023.10.25.23297558 42581323

[19] Brandon NJ , Millar JK , Korth C , Sive H , Singh KK , Sawa A . Understanding the role of DISC1 in psychiatric disease and during normal development. J Neurosci. 2009;29(41):12768‐12775. 19828788 10.1523/JNEUROSCI.3355-09.2009 PMC6665304

[20] Schiebout C , Lust H , Huang Y , Frost HR . Cell type‐specific interaction analysis using doublets in scRNA‐seq. Bioinform Adv. 2023;3(1):vbad120. 37745004 10.1093/bioadv/vbad120 PMC10516525

[21] McKenzie AT , Wang M , Hauberg ME , et al. Brain cell type specific gene expression and co‐expression network architectures. Sci Rep. 2018;8(1):8868. 29892006 10.1038/s41598-018-27293-5 PMC5995803

[22] Ritchie ME , Phipson B , Wu D , et al. limma powers differential expression analyses for RNA‐sequencing and microarray studies. Nucleic Acids Res. 2015;43(7):e47. 25605792 10.1093/nar/gkv007 PMC4402510

[23] Smyth G . Linear models and empirical bayes methods for assessing differential expression in microarray experiments. Stat Appl Genet Mol Biol. 2004;3:Article3. 16646809 10.2202/1544-6115.1027

[24] He B , Xiao Y , Liang H , et al. ASGARD is a single‐cell guided pipeline to aid repurposing of drugs. Nat Commun. 2023;14(1):993. 36813801 10.1038/s41467-023-36637-3 PMC9945835

[25] Wang M , Beckmann ND , Roussos P , et al. The Mount Sinai cohort of large‐scale genomic, transcriptomic and proteomic data in Alzheimer's disease. Sci Data. 2018;5(1):180185. 30204156 10.1038/sdata.2018.185 PMC6132187

[26] Duan Q , Flynn C , Niepel M , et al. LINCS Canvas Browser: interactive web app to query, browse and interrogate LINCS L1000 gene expression signatures. Nucleic Acids Res. 2014;42:W449‐60. 24906883 10.1093/nar/gku476 PMC4086130

[27] Duan Q , Reid SP , Clark NR , et al. L1000CDS2: lINCS L1000 characteristic direction signatures search engine. NPJ Syst Biol App. 2016;2(1):16015. 10.1038/npjsba.2016.15 PMC5389891 28413689

[28] Han H , Hahn S , Jeong HY , et al. LINCS L1000 dataset‐based repositioning of CGP‐60474 as a highly potent anti‐endotoxemic agent. Sci Rep. 2018;8(1):14969. 30297806 10.1038/s41598-018-33039-0 PMC6175892

[29] Karlsson M , Zhang C , Méar L , et al. A single–cell type transcriptomics map of human tissues. Sci Adv. 2021;7(31):eabh2169. 34321199 10.1126/sciadv.abh2169 PMC8318366

[30] Lonsdale J , Thomas J , Salvatore M , et al. The Genotype‐Tissue Expression (GTEx) project. Nature Gen. 2013;45(6):580‐585. 10.1038/ng.2653 PMC4010069 23715323

[31] Akol I , Izzo A , Gather F , et al. Multimodal epigenetic changes and altered NEUROD1 chromatin binding in the mouse hippocampus underlie FOXG1 syndrome. Proc Natl Acad Sci. 2023;120(2):e2122467120. 36598943 10.1073/pnas.2122467120 PMC9926245

[32] Durinck S , Moreau Y , Kasprzyk A , et al. BioMart and Bioconductor: a powerful link between biological databases and microarray data analysis. Bioinformatics. 2005;21(16):3439‐3440. 16082012 10.1093/bioinformatics/bti525

[33] Durinck S , Spellman PT , Birney E , Huber W . Mapping identifiers for the integration of genomic datasets with the R/Bioconductor package biomaRt. Nat Protocols. 2009; 4(8):1184‐1191. 19617889 10.1038/nprot.2009.97 PMC3159387

[34] Wu T , Hu E , Xu S , et al. clusterProfiler 4.0: a universal enrichment tool for interpreting omics data. Innovation. 2021;2(3):100141. 34557778 10.1016/j.xinn.2021.100141 PMC8454663

[35] Xu S , Hu E , Cai Y , et al. Using clusterProfiler to characterize multiomics data. Nat Protocols. 2024;19(11):3292‐3320. 39019974 10.1038/s41596-024-01020-z

[36] Yu G . Thirteen years of clusterProfiler Innovation. 2024;5(6):100722. 39529960 10.1016/j.xinn.2024.100722 PMC11551487

[37] Yu G , Wang L , Han Y , He Q . clusterProfiler: an R package for comparing biological themes among gene clusters. OMICS J Integr Biol. 2012;16(5):284‐287. 10.1089/omi.2011.0118 PMC3339379 22455463

[38] Stuart T , Butler A , Hoffman P , et al. Comprehensive integration of single‐cell data. Cell. 2019;177(7):1888‐1902. 31178118 10.1016/j.cell.2019.05.031 PMC6687398

[39] Cisternas P , Taylor X , Martinez P , Maldonado O , Jury N , Lasagna‐Reeves CA . The reduction of astrocytic tau prevents amyloid‐β‐induced synaptotoxicity. Brain Commun. 2022;4(5):fcac235. 36196088 10.1093/braincomms/fcac235 PMC9527666

[40] Zhang W , Liu H . MAPK signal pathways in the regulation of cell proliferation in mammalian cells. Cell Res. 2002;12(1):9‐18. 11942415 10.1038/sj.cr.7290105

[41] Francis SH , Busch JL , Corbin JD . cGMP‐dependent protein kinases and cGMP phosphodiesterases in nitric oxide and cGMP action. Pharmacol Rev. 2010;62(3):525‐563. 20716671 10.1124/pr.110.002907 PMC2964902

[42] Yoon S , Piguel N , Penzes P . Roles and mechanisms of ankyrin‐G in neuropsychiatric disorders. Mol Med. 2022;54(7):867‐877. 10.1038/s12276-022-00798-w PMC9356056 35794211

[43] Smith K , Penzes P . Ankyrins: roles in synaptic biology and pathology. Mol Cell Neurosci. 2018;91:131‐139. 29730177 10.1016/j.mcn.2018.04.010 PMC6128775

[44] Zhou T , Wang H . The role of glutamate dehydrogenase in the ageing brain. Front Pharmacol. 2025;16:1586655. 40356954 10.3389/fphar.2025.1586655 PMC12066631

[45] Zhu M , Tang M , Du Y . Identification of TAC1 associated with Alzheimer's disease using a robust rank aggregation approach. J Alzheimers Dis. 2023;91(4):1339‐1349. 36617784 10.3233/JAD-220950

[46] Morgan C , Inestrosa N . Interactions of laminin with the amyloid ß peptide: implications for Alzheimer's disease. Braz J Med Biol Res. 2001;34(5):597‐601. 11323745 10.1590/s0100-879x2001000500006

[47] Mao Y , Ge X , Frank CL , et al. Disrupted in schizophrenia 1 regulates neuronal progenitor proliferation via modulation of GSK3β/β‐Catenin signaling. Cell. 2009;136(6):1017‐1031. 19303846 10.1016/j.cell.2008.12.044 PMC2704382

[48] Kim JY , Duan X , Liu CY , et al. DISC1 regulates new neuron development in the adult brain via modulation of AKT‐mTOR signaling through KIAA1212. Neuron. 2009;63(6):761‐773. 19778506 10.1016/j.neuron.2009.08.008 PMC3075620

[49] Chen J , Liu Y , Zhou K , et al. <scp>DISC1</scp>inhibits <scp>GSK3β</scp>activity to prevent tau hyperphosphorylation under diabetic encephalopathy. BioFactors. 2023;49(1):173‐184. 36070513 10.1002/biof.1884

[50] Pei J , Braak E , Braak H , et al. Distribution of active glycogen synthase kinase 3β (GSK‐3β) in brains staged for Alzheimer's disease neurofibrillary changes. J Neuropathol Experiment Neurol. 1999;58(9):1010‐1019. 10.1097/00005072-199909000-00011 10499443

[51] Atkin T , Brandon N , Kittler J . Disrupted in schizophrenia 1 forms pathological aggresomes that disrupt its function in intracellular transport. Hum Mol Genet. 2012;21(9):2017‐2028. 22291444 10.1093/hmg/dds018

[52] Park SJ , Lee SB , Suh Y , et al. DISC1 modulates neuronal stress responses by gate‐keeping ER‐mitochondria Ca(2+) transfer through the MAM. Cell Rep. 2017;21(10):2748‐2759. 29212023 10.1016/j.celrep.2017.11.043

[53] MacAskill AF , Rinholm JE , Twelvetrees AE , et al. Miro1 is a calcium sensor for glutamate receptor‐dependent localization of mitochondria at synapses. Neuron. 2009;61(4):541‐555. 19249275 10.1016/j.neuron.2009.01.030 PMC2670979

[54] Wang X , Su B , Lee H , et al. Impaired balance of mitochondrial fission and fusion in Alzheimer's disease. J Neurosci. 2009;29(28):9090‐9103. 19605646 10.1523/JNEUROSCI.1357-09.2009 PMC2735241

[55] Kerr JS , Adriaanse BA , Greig NH , et al. Mitophagy and Alzheimer's disease: cellular and molecular mechanisms. Trends Neurosci. 2017;40(3):151‐166. 28190529 10.1016/j.tins.2017.01.002 PMC5341618

[56] Reddy PH , Tripathi R , Troung Q , et al. Abnormal mitochondrial dynamics and synaptic degeneration as early events in Alzheimer's disease: implications to mitochondria‐targeted antioxidant therapeutics. Biochim Biophys Acta. 2012;1822(5):639‐649. 22037588 10.1016/j.bbadis.2011.10.011 PMC3272314

[57] Millar JK , Mackie S , Clapcote SJ , et al. Disrupted in schizophrenia 1 and phosphodiesterase 4B: towards an understanding of psychiatric illness. J Physiol. 2007;584(2):401‐405. 17823207 10.1113/jphysiol.2007.140210 PMC2277141

[58] Baillie G . Compartmentalized signalling: spatial regulation of cAMP by the action of compartmentalized phosphodiesterases. The FEBS J. 2009;276(7):1790‐1799. 19243430 10.1111/j.1742-4658.2009.06926.x

[59] Hayashi‐Takagi A , Takaki M , Graziane N , et al. Disrupted‐in‐Schizophrenia 1 (DISC1) regulates spines of the glutamate synapse via Rac1. Nat Neurosci. 2010;13(3):327‐332. 20139976 10.1038/nn.2487 PMC2846623

[60] Wang Q , Charych EI , Pulito VL , et al. The psychiatric disease risk factors DISC1 and TNIK interact to regulate synapse composition and function. Mol Psychiatr. 2011;16(10):1006‐1023. 10.1038/mp.2010.87 PMC3176992 20838393

[61] Clapcote SJ , Lipina TV , Millar JK , et al. Behavioral phenotypes of Disc1 missense mutations in mice. Neuron. 2007;54(3):387‐402. 17481393 10.1016/j.neuron.2007.04.015

[62] Li W , Zhou Y , Jentsch JD , et al. Specific developmental disruption of disrupted‐in‐schizophrenia‐1 function results in schizophrenia‐related phenotypes in mice. Proc Natl Acad Sci U S A. 2007;104(46):18280‐18285. 17984054 10.1073/pnas.0706900104 PMC2084334

[63] Richter W , Menniti FS , Zhang H , Conti M . PDE4 as a target for cognition enhancement. Expert Opin Ther Targets. 2013;17(9):1011‐1027. 23883342 10.1517/14728222.2013.818656 PMC4066988

[64] Shankar GM , Bloodgood BL , Townsend M , Walsh DM , Selkoe DJ , Sabatini BL . Natural oligomers of the Alzheimer amyloid‐beta protein induce reversible synapse loss by modulating an NMDA‐type glutamate receptor‐dependent signaling pathway. J Neurosci. 2007;27(11):2866‐2875. 17360908 10.1523/JNEUROSCI.4970-06.2007 PMC6672572

[65] Vitolo OV , Sant'Angelo A , Costanzo V , Battaglia F , Arancio O , Shelanski M . Amyloid beta ‐peptide inhibition of the PKA/CREB pathway and long‐term potentiation: reversibility by drugs that enhance cAMP signaling. Proc Natl Acad Sci U S A. 2002;99(20):13217‐13221. 12244210 10.1073/pnas.172504199 PMC130613

[66] Beurel E , Grieco S , Jope R . Glycogen synthase kinase‐3 (GSK3): regulation, actions, and diseases. Pharmacol Ther. 2015;148:114‐131. 25435019 10.1016/j.pharmthera.2014.11.016 PMC4340754

[67] Rangaraju V , Lewis TL , Hirabayashi Y , et al. Pleiotropic mitochondria: the influence of mitochondria on neuronal development and disease. J Neurosci. 2019;39(42):8200‐8208. 31619488 10.1523/JNEUROSCI.1157-19.2019 PMC6794931

[68] Takashima A , Noguchi K , Michel G , et al. Exposure of rat hippocampal neurons to amyloid beta peptide (25‐35) induces the inactivation of phosphatidyl inositol‐3 kinase and the activation of tau protein kinase I/glycogen synthase kinase‐3 beta. Neurosci Lett. 1996;203(1):33‐36. 8742040 10.1016/0304-3940(95)12257-5

[69] Calkins MJ , Manczak M , Mao P , Shirendeb U , Reddy PH . Impaired mitochondrial biogenesis, defective axonal transport of mitochondria, abnormal mitochondrial dynamics and synaptic degeneration in a mouse model of Alzheimer's disease. Hum Mol Genet. 2011;20(23):4515‐4529. 21873260 10.1093/hmg/ddr381 PMC3209824

[70] Cummings J , Morstorf T , Zhong K . Alzheimer's disease drug‐development pipeline: few candidates, frequent failures. Alzheimers Res Ther. 2014;6(4):37. 25024750 10.1186/alzrt269 PMC4095696

[71] Karran E , De Strooper B . The amyloid cascade hypothesis: are we poised for success or failure?. J Neurochem. 2016;139:237‐252. 27255958 10.1111/jnc.13632

[72] Wang A , Fazari B , Chao OY , et al. Intra‐nasal dopamine alleviates cognitive deficits in tgDISC1 rats which overexpress the human DISC1 gene. Neurobiol Learn Mem. 2017;146:12‐20. 29107702 10.1016/j.nlm.2017.10.015

