# Supplementary material for: Single‐cell analysis reveals neuroprotective histone deacetylase inhibitor pathways
Source: Alzheimers Dement. 2026 Feb 3;22(2):e71108. doi: 10.1002/alz.71108 (PMC12865332; doi:10.1002/alz.71108)
Supplement: Supplementary file 1 — Supporting Information [file ALZ-22-e71108-s002.docx]

**Supplementary File: Single cell analysis reveals neuroprotective histone deacetylase inhibitor pathways**

Madeline Peyton, MS^a,b,c^, Nur Jury-Garfe, PhD^d,e^, Jiahui Liu, MS^a^, Caleb Beimfohr^f^, Chitra Sunil^g^, Steven Brooks, MS^a^, Pengyue Zhang, PhD, MS^a^, Sean D. McCabe, PhD, MS^a,3^, Timothy I. Richardson, PhD^c,f^, Kun Huang, PhD, MS^a,b,h^, Cristian A. Lasagna-Reeves, PhD^d,e^, Jie Zhang, PhD^h,i^, Travis S. Johnson, PhD, MSPH^a,c,h,^*

**Affiliations:**

^a^Department of Biostatistics and Health Data Science, Indiana University Indianapolis, Indianapolis, IN 46202, USA

^b^Regenstrief Institute, Indianapolis, IN 46202, USA

^c^Indiana Biosciences Research Institute, Indianapolis, IN 46202, USA

^d^Department of Anatomy, Cell Biology & Physiology, Indiana University Indianapolis, Indianapolis, IN 46202, USA

^e^Baylor College of Medicine, Houston, TX, 77030, USA

^f^Department of Medicine, Division of Clinical Pharmacology, School of Medicine, Indiana University Indianapolis, Indianapolis, IN 46202, USA

^g^Department of Environmental & Public Health Sciences, University of Cincinnati, Cincinnati, OH 45267, USA

^h^Indiana University Melvin and Bren Simon Comprehensive Cancer Center, Indianapolis, IN 46202, USA

^i^Department of Medical and Molecular Genetics, Indiana University Indianapolis, Indianapolis, IN 46202, USA

*Corresponding author: Travis S. Johnson, johnstrs@iu.edu, 410 W. 10th Street, HITS 3000, Indianapolis, IN 46202

# Supplementary Methods

## Spatial Transcriptomics

To investigate the spatial effects of TSA on the brain, a curated TSA gene signature from the LINCS L1000 [1-3] database was utilized. Spatial transcriptomics data were obtained from 10x Genomics, which included coronal sections of mouse brains from both AD models and healthy controls. The gene signature and spatial transcriptomics data were used to generate eigengenes and brain region-specific clusters, allowing the identification of areas in the mouse brain most likely to be affected by TSA treatment. Since the major neural cell types are highly conserved between mice and humans, these mouse data provide valuable insights into the potential effects of TSA on human brains. The integration of spatial transcriptomics data enhances the ability to map the effects of TSA to affected brain regions which is crucial for drug repurposing efforts. By understanding the spatial dynamics of gene expression changes induced by TSA, the therapeutic potential for treating AD was assessed.

## Tissue and Brain Region Expression Analysis

To characterize the expression patterns of DISC1 and other genes of interest across tissues and brain regions, we leveraged data from the Human Protein Atlas (HPA) [4] and Genotype-Tissue Expression (GTEx) [5] databases. The HPA database provides a comprehensive map of protein expression across tissues, organs, and cell types, while GTEx offers RNA expression profiles across multiple tissues. For each gene of interest, we extracted normalized expression values (transcripts per million, TPM) from these databases and analyzed their distribution patterns across tissues, with a particular focus on brain regions affected in AD. Additionally, scRNA-seq data from these databases were used to determine cell type-specific expression patterns, allowing for the identification of cell populations where these genes are predominantly expressed. This analysis provided valuable context for understanding the potential functional roles of these genes in normal brain function and their dysregulation in AD pathology.

# Supplementary Results

## Spatial Transcriptomics Reveals Brain Region-Specific Enrichment of TSA Signature

Spatial transcriptomics analysis using 10x Genomics Visium datasets and a TSA gene signature revealed distinct patterns of eigengene expression across different brain regions. The eigengene values, representing the TSA expression profile, were overlaid onto spatial transcriptomics data from the whole adult mouse brain, demonstrating regional variation in expression levels (**Fig. S4A**). A more focused analysis was conducted using data from specific coronal sections of the mouse brain. Eigengene values were mapped onto coronal section 1, highlighting significant enrichment in the hippocampus and cerebral cortex, as indicated by lighter shading in these areas (**Fig. S4B**). Similarly, eigengene values were mapped onto coronal section 2, which confirmed consistent patterns of enrichment in these same brain regions, reinforcing the reproducibility of the findings (**Fig. S4C**). These results suggest that the hippocampus and cortex are key regions of interest for TSA's potential therapeutic effects, offering valuable insights into its use in treating AD.

## DISC1 Expression Analysis Across Tissues and Brain Regions

Analysis of DISC1 RNA expression across multiple tissues revealed a striking pattern of enrichment (**Fig. S7A**). DISC1 exhibits its highest expression in the retina, where levels are markedly elevated compared to all other tissues. This exceptional retinal enrichment suggests a specialized role for DISC1 in visual processing and neuroretinal function. Beyond the retina, moderate DISC1 expression was observed in various brain regions, including the cerebral cortex, hippocampus, and amygdala. This distribution pattern, while less pronounced than in the retina, indicates DISC1's consistent involvement in central nervous system function. Outside the nervous system, low to moderate DISC1 expression was detected in reproductive tissues, adipose tissue, and certain immune-related organs such as the spleen and bone marrow. This broader tissue distribution suggests that DISC1 may have additional regulatory functions beyond neural activity, possibly influencing metabolic or immune-related pathways with potential relevance to neurodegenerative disorders.

When examining DISC1 expression across various brain regions, the data revealed highest expression levels in the white matter, thalamus, and pons (**Fig. S7B**). This distribution pattern aligns with DISC1's established role in microtubule-associated transport and neurodevelopmental processes. The white matter, primarily composed of myelinated axons, supports long-range neuronal connectivity, while the thalamus serves as a major relay center for sensory and cognitive processing. The pons, crucial for motor control and communication between brain regions, also exhibited elevated DISC1 expression, reinforcing its importance in neural integration and communication. In contrast, lower DISC1 expression levels were detected in the choroid plexus, hippocampal formation, and cerebellum. This finding is somewhat surprising given DISC1's previously documented role in hippocampal function and synaptic plasticity. The relatively lower expression in these regions suggests that DISC1's function here may depend more on protein interactions and subcellular localization rather than absolute RNA levels.

The single-cell RNA-seq data provides a high-resolution view of DISC1's distribution across diverse cell types (**Fig. S7C**). The results showed pronounced elevation of DISC1 expression in neuronal cells, particularly in rod photoreceptor cells. This finding reinforces the tissue-level observation of retinal enrichment and suggests a specialized role in retinal function. In addition to strong expression in photoreceptor cells, moderate DISC1 expression was observed in other neuronal cell types, including cone photoreceptors and bipolar cells. This neuronal enrichment underscores DISC1's potential role in sensory processing and neuroprotective mechanisms in the visual system, which may provide insights into its broader role in neuronal health and resilience. Conversely, lower DISC1 expression was observed in glial cells, including Schwann cells, suggesting a more limited role in myelination and glial-mediated support functions. However, its moderate presence in blood and immune cells implies a potential role in neuroimmune interactions, which could be relevant in neurodegenerative diseases such as AD.

We can analyze the median expression levels of DISC1 across various brain regions, measured in RNA-seq counts per million (**Fig. S7D**). This analysis revealed highest expression in the parahippocampal gyrus (PHG), superior temporal gyrus (STG), and frontal pole (FP), while the cerebellum exhibited the lowest expression. Notably, these high-expression regions are implicated in cognitive function, memory processing, and are among the earliest affected in AD. The parahippocampal gyrus, which exhibited the highest DISC1 expression, is critically involved in episodic memory and spatial navigation and is one of the earliest regions affected by neurofibrillary tangle accumulation in AD pathology. Similarly, the frontal pole and superior temporal gyrus both showed significant DISC1 expression. The frontal pole, associated with higher cognitive functions, experiences atrophy in AD patients that correlates with impairments in recall abilities. The superior temporal gyrus, involved in auditory processing and language comprehension, exhibits significant gene expression abnormalities in individuals with mild to moderate dementia. DISC1's prominent expression in these regions further supports its potential involvement in AD-related cognitive decline.

# References

1. - Todd Golub, A.S., *- L1000 Dataset -small molecule perturbagens- LINCS Trans-Center Project*. 2014.

2. Duan, Q., et al., *LINCS Canvas Browser: interactive web app to query, browse and interrogate LINCS L1000 gene expression signatures.* Nucleic Acids Res, 2014. **42**(Web Server issue): p. W449–60.

3. Duan, Q., et al., *L1000CDS2: LINCS L1000 characteristic direction signatures search engine.* npj Systems Biology and Applications, 2016. **2**(1): p. 16015.

4. Karlsson, M., et al., *A single–cell type transcriptomics map of human tissues.* Science Advances, 2021. **7**(31): p. eabh2169.

5. Lonsdale, J., et al., *The Genotype-Tissue Expression (GTEx) project.* Nature Genetics, 2013. **45**(6): p. 580–585.

6. He, B., et al., *ASGARD: A Single-cell Guided pipeline to Aid Repurposing of Drugs.* ArXiv, 2021.

7. Grubman, A., et al., *A single-cell atlas of entorhinal cortex from individuals with Alzheimer's disease reveals cell-type-specific gene expression regulation.* Nat Neurosci, 2019. **22**(12): p. 2087–2097.

8. Mathys, H., et al., *Single-cell transcriptomic analysis of Alzheimer's disease.* Nature, 2019. **570**(7761): p. 332–337.

9. Green, G.S., et al., *Cellular communities reveal trajectories of brain ageing and Alzheimer's disease.* Nature, 2024. **633**(8030): p. 634–645.

10. Lee, D., et al., *Plasticity of Human Microglia and Brain Perivascular Macrophages in Aging and Alzheimer’s Disease*. 2023, Cold Spring Harbor Laboratory.

# Supplementary Tables

**Table S1.** Drug repurposing candidates from entorhinal cortex analysis. Drug candidates identified through ASGARD [6] analysis of the Grubman et al. [7] dataset showing significant FDR values (<0.1) across different cell types. Drug coverage represents the percentage of total cell types affected by each compound. Cell type abbreviations: Neu (neurons), Dou (doublets), Oli (oligodendrocytes), Mic (microglia), unID (unidentified cells), Opc (oligodendrocyte progenitor cells), Ast (astrocytes), End (endothelial cells). Cell type coverage indicates the percentage of cells within each specific cell type. FDR values represent false discovery rate-adjusted p-values for drug-cell type associations.

| Drug | Drug coverage | Cell type | Cell type coverage | FDR |
| --- | --- | --- | --- | --- |
| TSA | 77.91 | Neu | 3.73 | 2.52E-05 |
|  |  | Dou | 4.42 | 4.44E-04 |
|  |  | Oli | 69.76 | 9.94E-03 |
| SB-939 | 16.2 | Mic | 2.58 | 7.66E-05 |
|  |  | unID | 9.2 | 1.07E-03 |
|  |  | Dou | 4.42 | 5.07E-03 |
| vorinostat | 13.62 | Dou | 4.42 | 2.28E-10 |
|  |  | unID | 9.2 | 7.90E-02 |
| belinostat | 13.62 | Dou | 4.42 | 7.29E-06 |
|  |  | unID | 9.2 | 6.99E-05 |
| JNK-IN-5A | 11.78 | Mic | 2.58 | 2.18E-03 |
|  |  | unID | 9.2 | 7.90E-02 |
| Mirdametinib | 8.99 | Neu | 3.73 | 1.01E-12 |
|  |  | Opc | 2.68 | 1.47E-07 |
|  |  | Mic | 2.58 | 5.93E-04 |
| GSK-1059615 | 7.07 | Ast | 7.07 | 6.50E-03 |
| AZ-628 | 6.31 | Mic | 2.58 | 1.59E-05 |
|  |  | Neu | 3.73 | 7.49E-03 |
| rigosertib | 2.58 | Mic | 2.58 | 7.35E-04 |

**Table S2.** Drug repurposing candidates from prefrontal cortex analysis. Drug candidates identified through ASGARD [6] analysis of the Mathys et al. [8] scRNA-seq dataset. All compounds shown have significant FDR values (<0.1) in at least one cell type. Drug coverage indicates the percentage of total cell types affected. Cell type abbreviations: Opc (oligodendrocyte progenitor cells), Mic (microglia), Per (pericytes).

| Drug | Drug coverage | Cell type | Cell type coverage | FDR |
| --- | --- | --- | --- | --- |
| CGP-60474 | 3.63 | Opc | 3.63 | 2.72E-05 |
| GSK-1059615 | 3.63 | Opc | 3.63 | 6.39E-04 |
| TSA | 3.63 | Opc | 3.63 | 1.01E-02 |
| staurosporine | 3.63 | Opc | 3.63 | 2.06E-02 |
| tanespimycin | 3.63 | Opc | 3.63 | 3.19E-02 |
| BRD-K22828860 | 3.63 | Opc | 3.63 | 7.82E-02 |
| Mirdametinib | 2.69 | Mic | 2.69 | 8.74E-10 |
| dasatinib | 0.21 | Per | 0.21 | 1.67E-02 |

**Table S3.** Drug repurposing candidates from aged prefrontal cortex. Drug candidates identified through ASGARD [6] analysis of the Green et al. [9] scRNA-seq dataset. All compounds shown have significant FDR values (<0.1) in at least one cell type. Drug coverage indicates the percentage of total cell types affected. Cell type abbreviations: Oligo (oligodendrocyte), Ex_cux2plus (Excitatory neurons, CUX2+), Ex_cux2minus (Excitatory neurons, CUX2-), Inh (Inhibitory neurons), Astro (astrocytes).

| Drug | Drug coverage | Cell type | Cell type coverage | FDR |
| --- | --- | --- | --- | --- |
| CGP-60474 | 79.77 | Oligo | 25.07 | 1.00E-08 |
|  |  | Ex_cux2plus | 22.8 | 7.95E-05 |
|  |  | Inh | 15.37 | 4.53E-04 |
|  |  | Ex_cux2minus | 16.53 | 5.03E-03 |
| staurosporine | 63.24 | Oligo | 25.07 | 1.76E-02 |
|  |  | Ex_cux2plus | 22.8 | 4.51E-02 |
|  |  | Inh | 15.37 | 6.99E-02 |
| MK-1775 | 47.87 | Ex_cux2plus | 22.8 | 9.75E-05 |
|  |  | Oligo | 25.07 | 5.92E-04 |
| tanespimycin | 47.87 | Oligo | 25.07 | 5.65E-03 |
|  |  | Ex_cux2plus | 22.8 | 6.71E-02 |
| chelerythrine | 25.07 | Oligo | 25.07 | 1.09E-04 |
| JNK-IN-5A | 25.07 | Oligo | 25.07 | 5.92E-04 |
| ponatinib | 25.07 | Oligo | 25.07 | 1.62E-02 |
| mitoxantrone | 25.07 | Oligo | 25.07 | 3.19E-02 |
| AGK-2 | 25.07 | Oligo | 25.07 | 4.56E-02 |
| rebastinib | 25.07 | Oligo | 25.07 | 7.49E-02 |
| GSK-461364 | 22.8 | Ex_cux2plus | 22.8 | 1.24E-02 |
| NVP-BGJ398 | 22.8 | Ex_cux2plus | 22.8 | 6.71E-02 |
| cimetidine | 22.8 | Ex_cux2plus | 22.8 | 6.71E-02 |
| GSK-1059615 | 13.79 | Astro | 13.79 | 1.18E-07 |
| MLN-0128 | 13.79 | Astro | 13.79 | 8.25E-03 |
| SB-939 | 5.36 | Microglia | 5.36 | 3.12E-02 |
| belinostat | 5.36 | Microglia | 5.36 | 5.86E-02 |
| vorinostat | 5.36 | Microglia | 5.36 | 7.92E-02 |

**Table S4.** Top-ranked drug candidates significant across all three independent

AD datasets. Drug candidates identified as significant (FDR < 0.1) in all three independent single-cell RNA sequencing datasets: Grubman et al. [7] entorhinal cortex, Mathys et al. [8] prefrontal cortex, and Green et al. [9] prefrontal cortex. Drugs are ranked by their maximum drug therapeutic score across the three datasets. Drug therapeutic scores were calculated using the ASGARD [6] framework, which integrates differential gene expression patterns between disease and control samples with known drug-gene interactions from the Connectivity Map (CMap) database. Higher drug scores indicate stronger predicted therapeutic potential based on inverse correlation between drug-induced gene expression signatures and disease-associated expression changes. For each drug, results from all three datasets are shown: drug therapeutic score, nominal p-value, and false discovery rate (FDR). Empty cells in the 'Drug' column indicate continuation of the same drug across datasets.

| Drug | Dataset | Drug Score | P-value | FDR |
| --- | --- | --- | --- | --- |
| CGP-60474 | Green | 2.43E+00 | 1.17E-22 | 4.07E-19 |
|  | Grubman | 3.83E-09 | 3.97E-04 | 8.12E-02 |
|  | Mathys | 1.66E-01 | 2.72E-11 | 4.74E-08 |
| GSK-1059615 | Green | 6.66E-01 | 7.74E-14 | 8.98E-11 |
|  | Grubman | 9.32E-02 | 6.78E-09 | 2.95E-06 |
|  | Mathys | 4.84E-02 | 1.76E-06 | 8.77E-04 |
| TSA | Green | 5.27E-02 | 1.23E-03 | 8.60E-02 |
|  | Grubman | 5.69E-01 | 1.36E-20 | 2.37E-17 |
|  | Mathys | 3.44E-02 | 7.77E-07 | 4.50E-04 |

**Table S5.** Drug candidates after doublet cell re-annotation analysis. Drug repurposing candidates identified from the Grubman et al. [7] dataset following re-annotation of doublet cells based on their top two contributing cell identities. Drug coverage represents the percentage of total annotated cell populations affected by each compound. Cell type abbreviations include single cell types (Opc, Mic, Ast, Oli, End, unID) and doublet combinations (neu-opc: neuron-oligodendrocyte progenitor, end-mic: endothelial-microglia, mic-opc: microglia-oligodendrocyte progenitor, end-opc: endothelial-oligodendrocyte progenitor). Cell type coverage indicates the percentage representation within each specific population. FDR values represent statistical significance of drug-cell type associations.

| Drug | Drug coverage | Cell type | Cell type coverage | FDR |
| --- | --- | --- | --- | --- |
| mirdametinib | 92.63 | Opc | 9.52 | 7.48E-08 |
|  |  | unID | 1.45 | 1.04E-06 |
|  |  | Mic | 3.9 | 2.19E-04 |
|  |  | Ast | 18.61 | 2.52E-04 |
|  |  | neu-opc | 0.06 | 6.75E-04 |
|  |  | end-mic | 0.15 | 8.11E-04 |
|  |  | Oli | 58.94 | 3.49E-02 |
| TSA | 71.19 | end-mic | 0.15 | 1.03E-07 |
|  |  | unID | 1.45 | 1.04E-06 |
|  |  | mic-opc | 0.13 | 2.47E-03 |
|  |  | end-opc | 0.16 | 2.67E-03 |
|  |  | Oli | 58.94 | 3.99E-03 |
|  |  | End | 0.84 | 1.94E-02 |
|  |  | Opc | 9.52 | 5.24E-02 |
| rigosertib | 9.52 | Opc | 9.52 | 2.88E-02 |
| AZ-628 | 1.45 | unID | 1.45 | 1.63E-03 |
| XMD-885 | 0.15 | end-mic | 0.15 | 6.01E-02 |
| mocetinostat | 0.13 | mic-opc | 0.13 | 2.47E-03 |
| entinostat | 0.13 | mic-opc | 0.13 | 1.68E-02 |
| ponatinib | 0.13 | mic-opc | 0.13 | 1.68E-02 |
| geldanamycin | 0.06 | neu-opc | 0.06 | 3.74E-02 |

**Table S6.** Cell-type-specific targeting profile of top drug candidates across three independent AD datasets. Cell-type-specific drug effects for candidates identified as significant (FDR < 0.1) across three independent single-cell RNA sequencing datasets: Grubman et al. entorhinal cortex, Mathys et al. prefrontal cortex, and Green et al. prefrontal cortex. For each drug, only cell types where the drug showed significant effects (FDR < 0.1) are displayed. Cell type coverage indicates the percentage of cells in each dataset belonging to that cell type. Drug coverage indicates the percentage of cell types within each dataset where the drug demonstrated significant therapeutic potential. Within each dataset, cell types are ordered by their abundance (cell type coverage). Drugs are ordered by their maximum drug therapeutic score across the three datasets. Drug therapeutic scores were calculated using ASGARD separately for each cell type, enabling identification of cell-type-specific therapeutic mechanisms. Abbreviations: Ast, astrocytes; End, endothelial cells; Ex, excitatory neurons; Inh, inhibitory neurons; Mic, microglia; Oli, oligodendrocytes; Opc, oligodendrocyte precursor cells; unID, unidentified cells.

| Drug | Dataset | Cell type | Cell type coverage | Drug coverage | FDR |
| --- | --- | --- | --- | --- | --- |
| CGP-60474 | Mathys | Opc | 3.63 | 3.63 | 2.72E-05 |
|  | Green | Ex | 39.33 | 79.77 | 5.93E-05 |
|  |  | Oli | 25.07 | 79.77 | 1.00E-08 |
|  |  | Inh | 15.37 | 79.77 | 4.53E-04 |
| GSK-1059615 | Mathys | Opc | 3.63 | 3.63 | 6.39E-04 |
|  | Green | Ast | 13.79 | 13.79 | 1.18E-07 |
| TSA | Grubman | Oli | 58.94 | 71.19 | 3.99E-03 |
|  |  | OPC | 9.52 | 71.19 | 5.24E-02 |
|  |  | unID | 1.45 | 71.19 | 1.04E-06 |
|  |  | End | 0.84 | 71.19 | 1.94E-02 |
|  |  | End-Opc | 0.16 | 71.19 | 2.67E-03 |
|  |  | End-Mic | 0.15 | 71.19 | 1.03E-07 |
|  |  | Mic-Opc | 0.13 | 71.19 | 2.47E-03 |
|  | Mathys | Opc | 3.63 | 3.63 | 1.01E-02 |

**Table S7.** Shared dysregulated signaling pathways in AD across independent datasets. Significantly enriched signaling pathways identified in both the Grubman et al. [7], Mathys et al. [8] and Green et al. [9] datasets. Cell-type specific enrichment is reported, with -log10(FDR) shown for each pathway-cell type pairing. Ex = excitatory neurons, In = inhibitory neurons, Ast = astrocytes, Mic = microglia, Neu = neurons, Oli = oligodendrocytes, Opc = oligodendrocyte progenitor cells, Dou = doublets, Vasc = vascular cells.

| Pathway | Dataset | Cell type | -log_10_(FDR) |
| --- | --- | --- | --- |
| Estrogen signaling pathway | Green | In | 1.54 |
|  |  | Vasc | 1.89 |
|  | Grubman | Ast | 1.37 |
|  |  | Dou | 1.63 |
|  |  | Oli | 1.35 |
| MAPK signaling pathway | Green | Mic | 2.29 |
|  |  | Vasc | 3.35 |
|  | Grubman | Ast | 1.79 |
|  |  | Oli | 1.44 |
|  | Mathys | In | 2.34 |
| Oxytocin signaling pathway | Green | Vasc | 2.5 |
|  | Mathys | Ex | 1.9 |
|  |  | In | 2.07 |
|  |  | Oli | 1.67 |
| PI3K-Akt signaling pathway | Green | Vasc | 3.35 |
|  | Grubman | Dou | 1.63 |
| Rap1 signaling pathway | Green | Vasc | 4.2 |
|  | Grubman | Oli | 1.44 |
| cAMP signaling pathway | Grubman | Neu | 1.65 |
|  | Mathys | Ex | 1.73 |
|  |  | In | 1.61 |
| cGMP-PKG signaling pathway | Green | Vasc | 1.34 |
|  | Mathys | Ast | 2.41 |
|  |  | Ex | 2.9 |
|  |  | In | 1.47 |

# Supplementary Figures


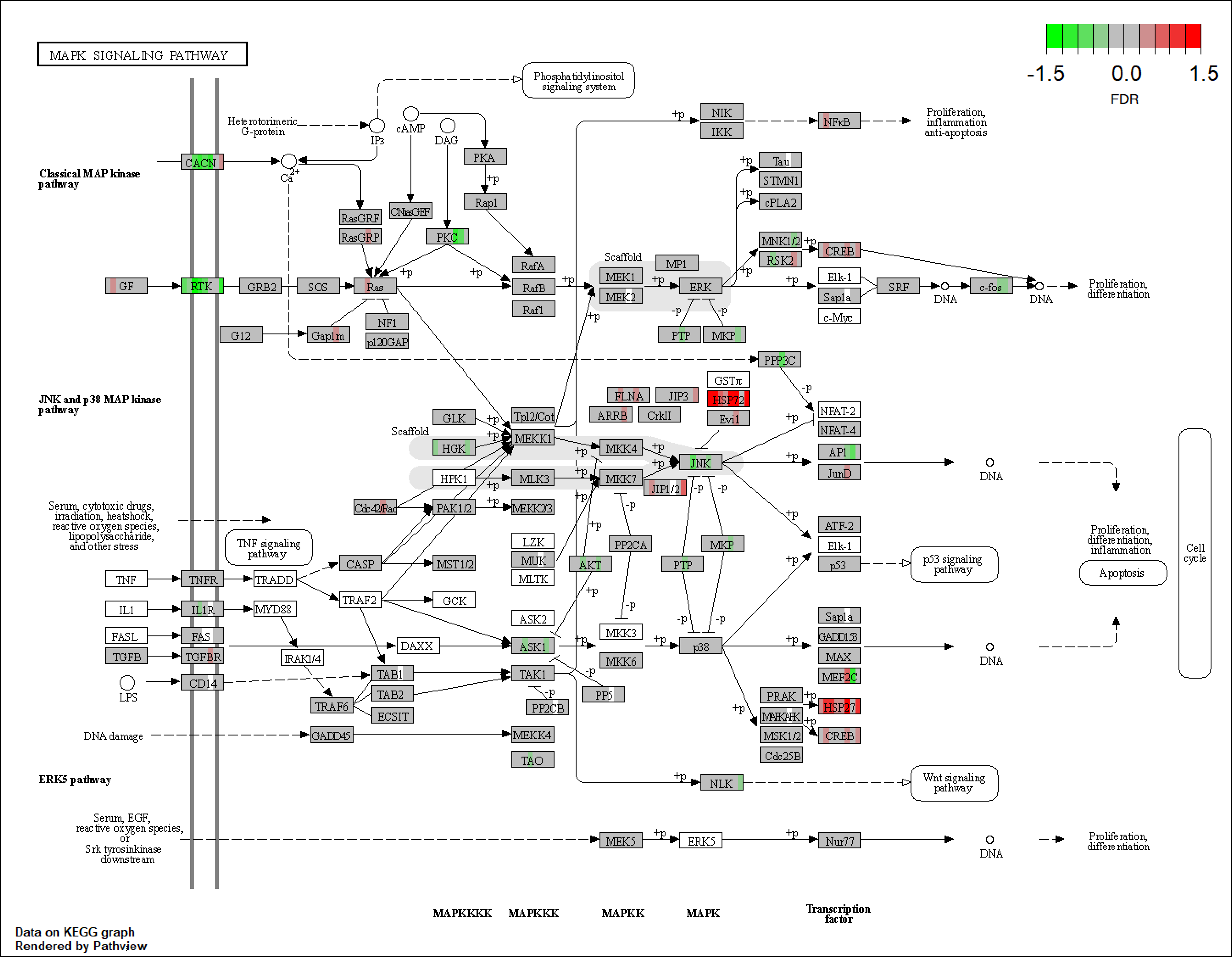


**Fig. S1** Mitogen-Activated Protein Kinase (MAPK) signaling pathway in Homo sapiens visualized with KEGG graph. Differential gene expression is shown for each cell type with a color-coded scale representing the False Discovery Rate (FDR). Each gene box is divided to represent expression across cell types from left to right: oligodendrocytes, unidentified cells, astrocytes, oligodendrocyte progenitor cells, neurons, endothelial cells, microglia, and doublet cells.


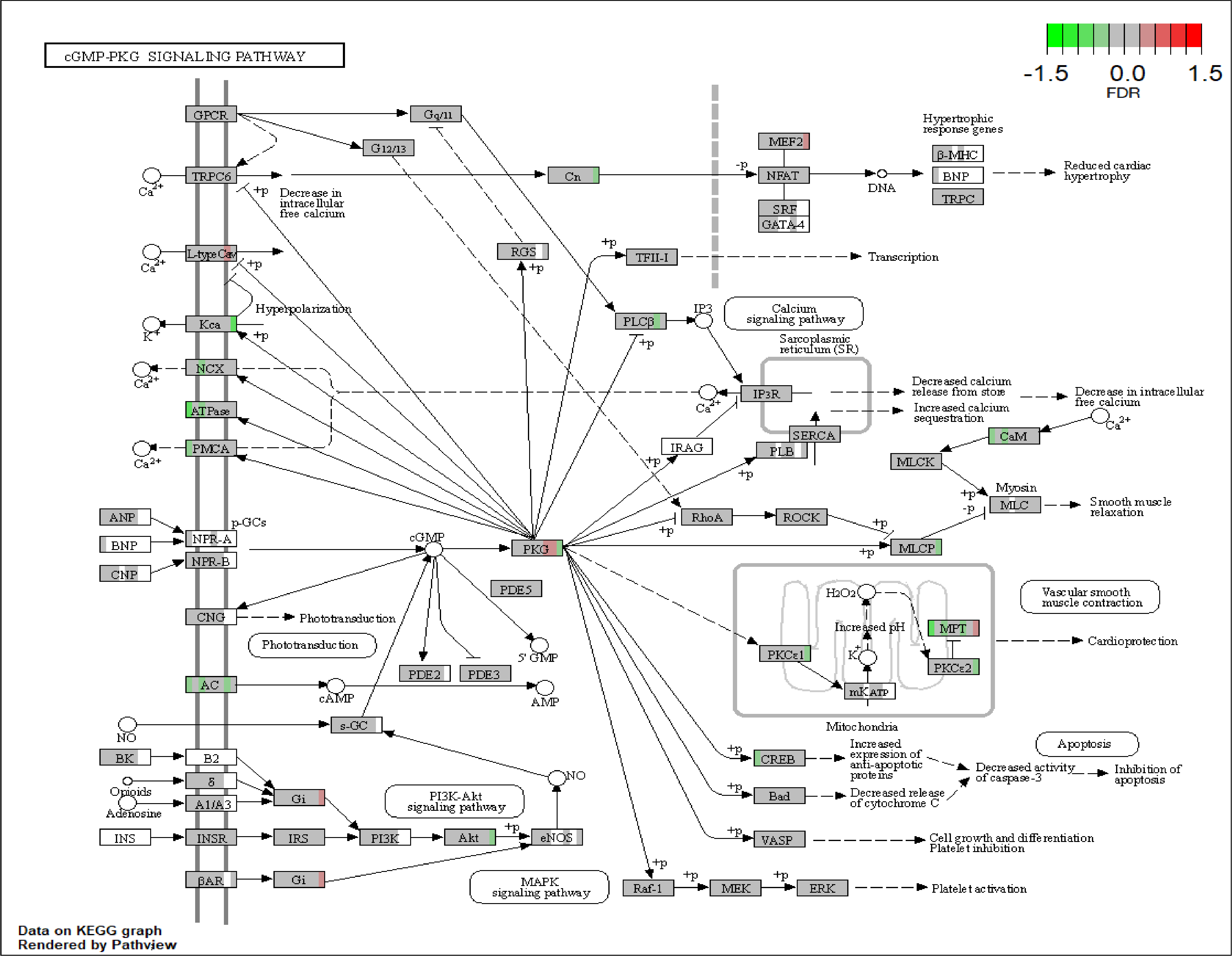


**Fig. S2** Cyclic guanosine monophosphate - protein kinase G (cGMP-PKG) signaling pathway in Homo sapiens visualized using KEGG graph representation. Differential gene expression is shown for each cell type with a color-coded scale representing the FDR. Each gene box is divided to represent expression across cell types from left to right: excitatory neurons, oligodendrocytes, inhibitory neurons, microglia, oligodendrocyte progenitor cells, astrocytes, pericytes, and endothelial cells.


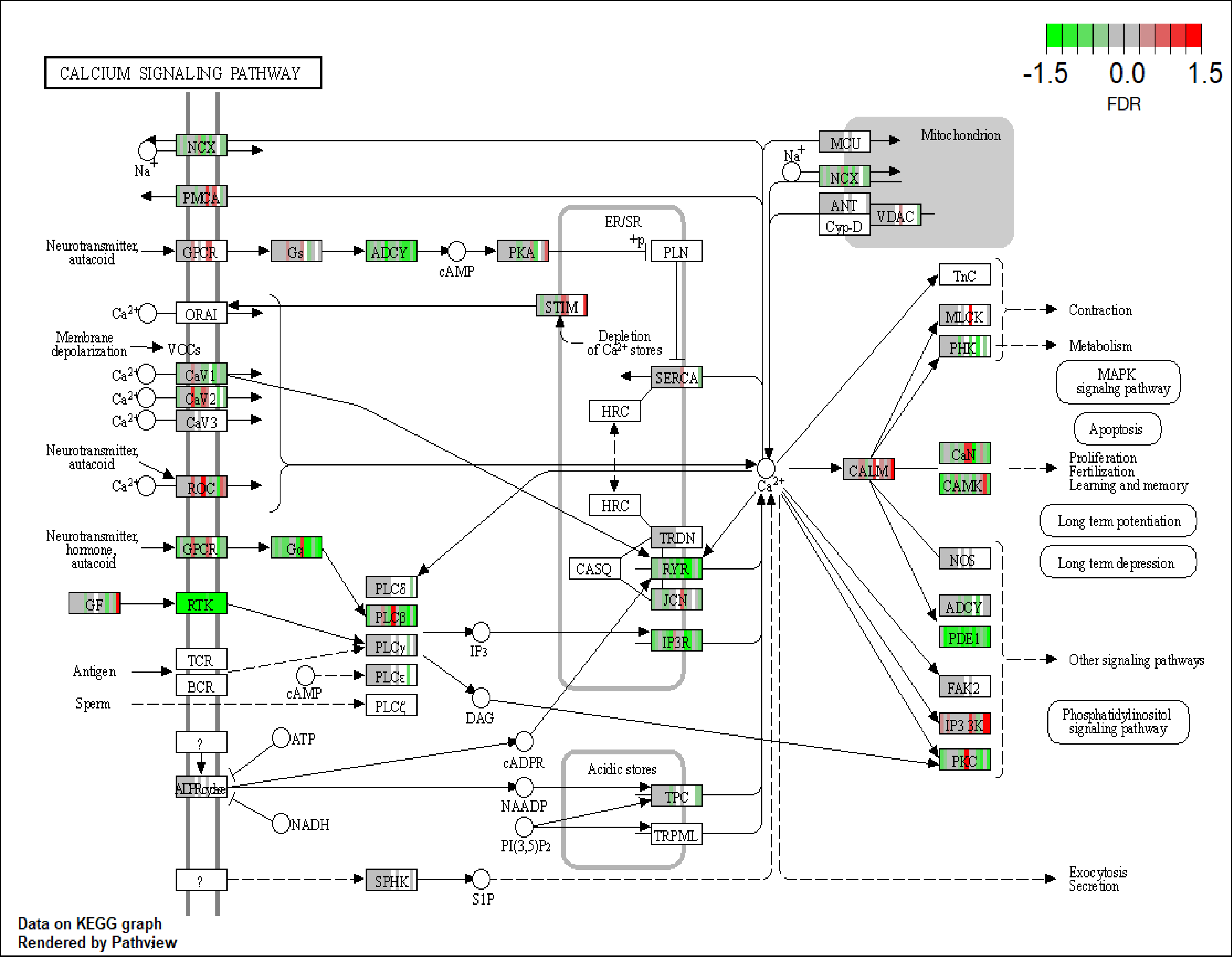


**Fig. S3** Calcium signaling pathway in Homo sapiens visualized using KEGG graph representation. Differential gene expression is shown for each cell type with a color-coded scale representing the FDR. Each gene box is divided to represent expression across cell types from left to right: oligodendrocytes, unidentified cells, astrocytes, oligodendrocyte progenitor cells, neurons, endothelial cells, microglia, microglia-oligodendrocytes, oligodendrocytes-oligodendrocyte progenitor cells, microglia-oligodendrocyte progenitor cells, astrocyte-neurons, endothelial-oligodendrocyte progenitor cells, endothelial-microglia, and neuron-oligodendrocyte progenitor cells.


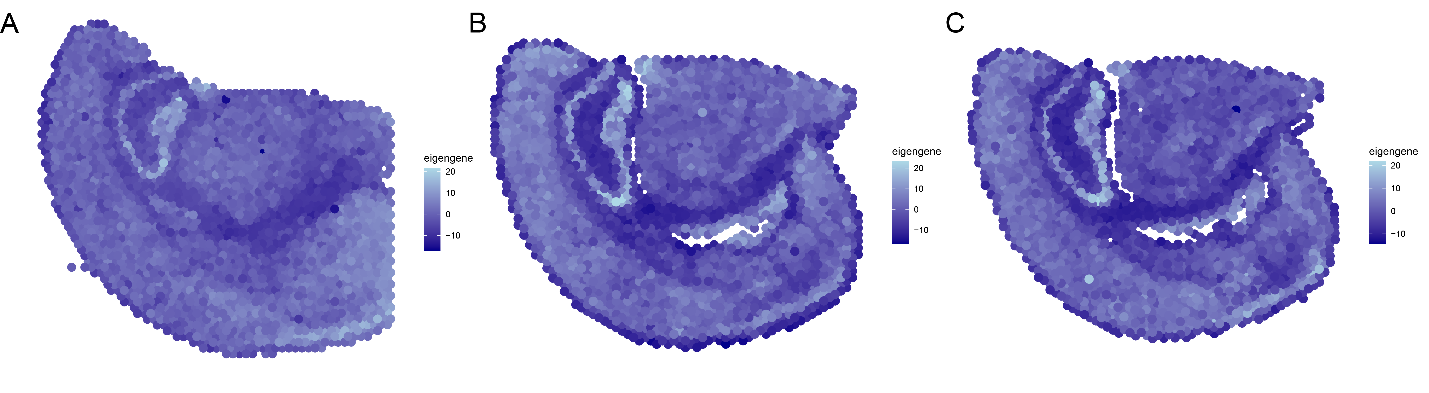


**Fig. S4** Eigengene Values in Spatial Transcriptomics of Three Mouse Brain Samples **A)** Eigengene values overlaid onto spatial transcriptomics of the adult mouse brain **B)** Eigengene values overlaid onto spatial transcriptomics of the mouse brain coronal section 1 **C)** Eigengene values overlaid onto spatial transcriptomics of the mouse brain coronal section 2

**Fig. S5** Cell prioritization and drug repurposing analysis across three independent AD single-cell datasets (A-C) Distribution of AD-association scores (hazard scores) for neurons stratified by AD diagnosis status across three independent datasets: (A) Grubman et al. entorhinal cortex dataset showing all neuronal populations, (B) Mathys et al. prefrontal cortex dataset showing excitatory (Ex) and inhibitory (In) neurons, and (C) Green et al. aged prefrontal cortex dataset showing CUX2-positive excitatory neurons, CUX2-negative excitatory neurons, and inhibitory neurons. Scores reflect similarity to known AD transcriptomic signatures, with higher scores indicating greater AD-association. (D-F) Ranking positions of HDAC inhibitors in L1000CDS² queries across datasets and neuronal populations. (D) Reverse queries identifying compounds that oppose disease signatures. (E) Mimic queries identifying compounds that reproduce disease signatures. (F) Total count of HDAC inhibitor signatures appearing in the top 50 ranked compounds for each query type and dataset. (G-H) UpSet plots showing intersection of differentially expressed genes across datasets and neuronal populations. (G) Upregulated genes in high AD-associated neurons. (H) Downregulated genes in high AD-associated neurons. Horizontal bars indicate set size for each dataset; vertical bars indicate intersection size with connecting dots showing which datasets contribute to each intersection. (I) Heatmap of log2 fold changes for selected differentially expressed genes across datasets and neuronal populations, highlighting genes with consistent dysregulation patterns or cell-type-specific effects. (J) Gene Ontology Biological Process enrichment analysis for downregulated genes, showing -log10(FDR) values for significantly enriched pathways across datasets.

**Fig. S6** Cross-species conservation of TSA-induced transcriptional responses in mouse hippocampal and human iPSC-derived cortical neurons A) Venn diagram showing overlap of genes significantly upregulated by TSA treatment (p < 0.05, log₂FC > 0.58) in mouse primary hippocampal neurons (n = 3,084 genes) and human iPSC-derived cortical neurons (n = 1,572 genes), with 856 genes showing conserved upregulation. Venn diagram showing overlap of significantly downregulated genes in both systems, with 813 genes showing conserved downregulation. B) Scatter plot of log₂ fold-changes for orthologous genes, demonstrating correlation of TSA effects across species. Conserved genes (present in both datasets with same direction) are shown in red; species-specific genes are shown in gray. Pearson r = 0.663 for all orthologs, r = 0.829 for conserved genes. C) Heatmap displaying normalized log₂ fold-changes of the top 30 conserved TSA-responsive genes (ranked by mean absolute log₂FC), demonstrating consistent regulation patterns across mouse and human neuronal systems. D) Gene Ontology Biological Process enrichment analysis of 1,669 genes showing conserved TSA responses across mouse and human neurons. Top enriched pathways include regulation of neuron projection development, axonogenesis, synapse organization, and cognition. E) Gene Ontology Molecular Function enrichment analysis showing enrichment in DNA-binding transcription factor binding, cytoskeletal protein binding (actin and tubulin), and histone kinase activity.


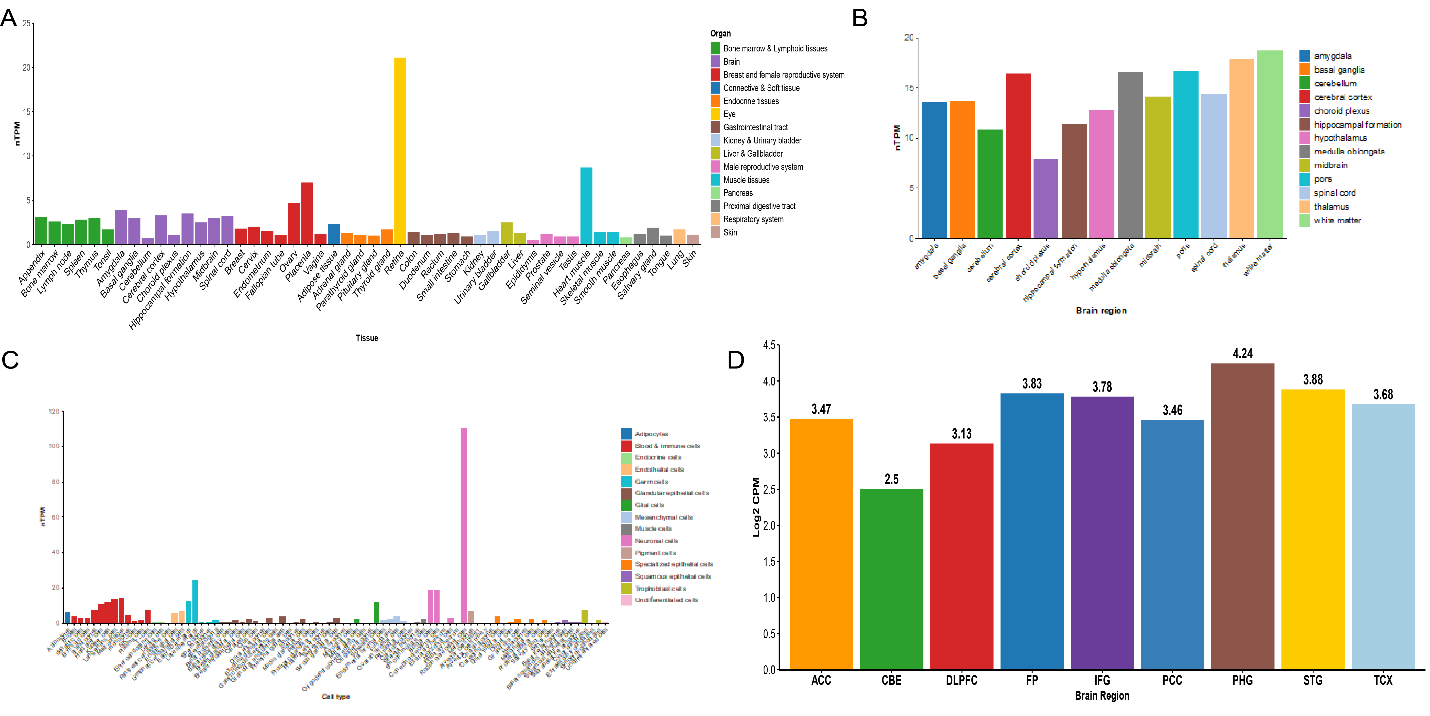


**Fig. S7** DISC1 Expression Distribution Across Tissues and Brain Regions in AD Context **A)** RNA expression of DISC1 across multiple tissues displayed as normalized transcripts per million (nTPM), showing remarkably high expression in the retina and moderate expression in brain tissues (purple bars) **B)** Brain region-specific DISC1 expression reveals highest levels in white matter, thalamus, and pons, with moderate expression in other neural regions, suggesting specialized roles in neural connectivity and sensory processing **C)** Single-cell RNA expression analysis demonstrating pronounced DISC1 enrichment in rod photoreceptor cells (tall pink bar), with moderate expression in various neuronal populations and lower expression in glial cells **D)** Median expression levels of DISC1 across various brain regions measured in RNA-seq counts per million (CPM, log2-transformed). The parahippocampal gyrus (PHG), superior temporal gyrus (STG), and frontal pole (FP) show highest expression, all areas implicated in cognitive function and early AD pathology, suggesting DISC1's potential neuroprotective role in these vulnerable regions


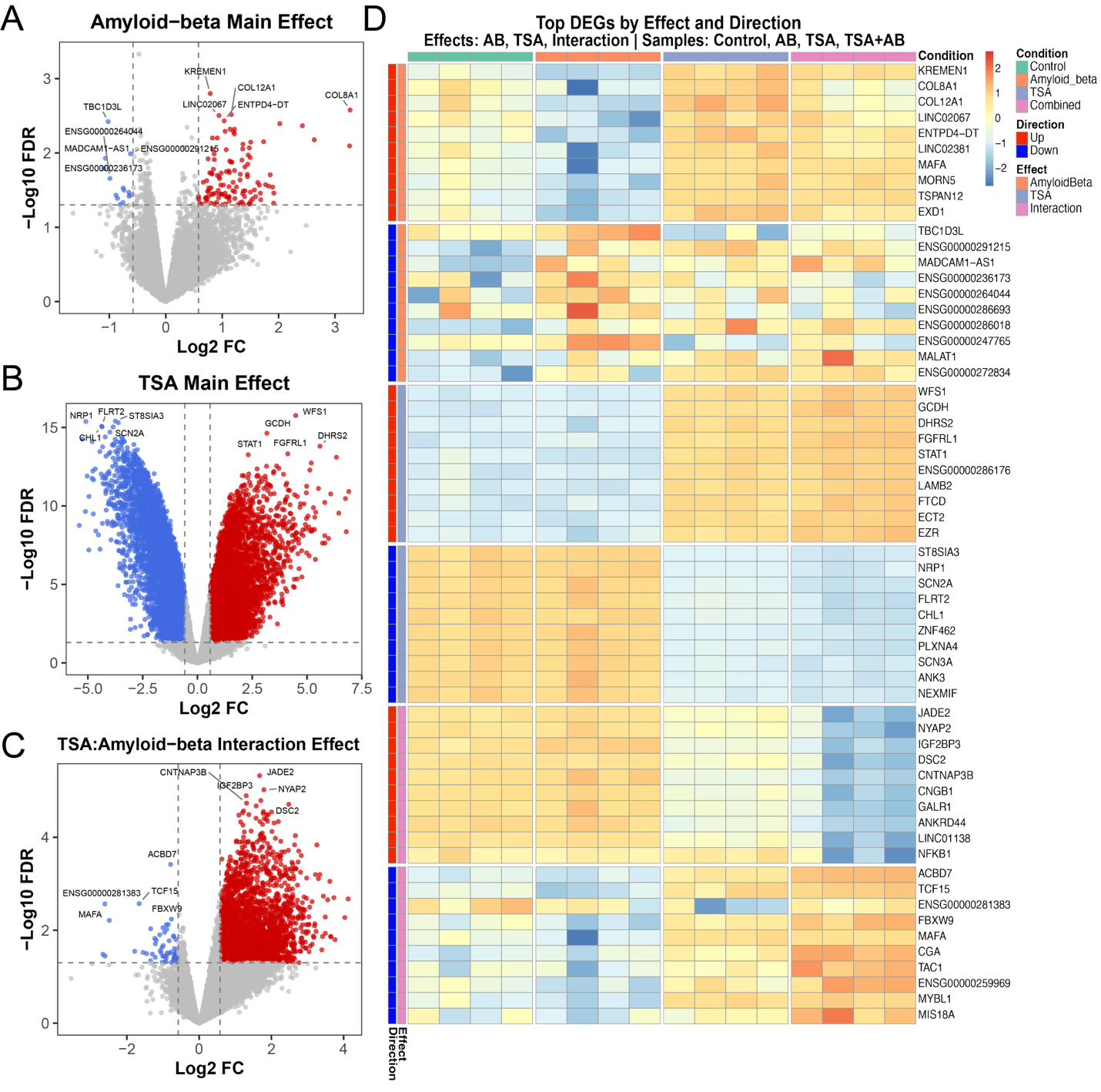


**Fig. S8** Differential Gene Expression Analysis of TSA and Amyloid-Beta Treatment in Neural Cells **A)** Volcano plot showing differentially expressed genes in response to amyloid-beta treatment. Blue points represent significantly down-regulated genes while red points represent significantly up-regulated genes (p < 0.05, |log2FC| > 0.58) **B)** Volcano plot showing gene expression changes induced by TSA treatment **C)** Volcano plot demonstrating the interaction effect between TSA and amyloid-beta treatments **D)** Heatmap of top differentially expressed genes across all experimental conditions, clustered by effect and expression pattern. Color bars indicate treatment conditions and gene characteristics (direction of regulation and primary effect). Several Ensembl IDs in this analysis could not be mapped to conventional gene names as they represent genomic regions that have not been sufficiently characterized or annotated in current databases.

# Supplementary files

**Files S1-S18.** L1000CDS² drug repurposing query results and differential gene expression signatures for AD-associated neuronal populations across three independent datasets.

**File S1.** Grubman et al. entorhinal cortex neurons - L1000CDS² mimic query results

**File S2.** Grubman et al. entorhinal cortex neurons - L1000CDS² reverse query results

**File S3.** Grubman et al. entorhinal cortex neurons - differential gene expression results

**File S4.** Mathys et al. prefrontal cortex excitatory neurons - L1000CDS² mimic query results

**File S5.** Mathys et al. prefrontal cortex excitatory neurons - L1000CDS² reverse query results

**File S6.** Mathys et al. prefrontal cortex excitatory neurons - differential gene expression results

**File S7.** Mathys et al. prefrontal cortex inhibitory neurons - L1000CDS² mimic query results

**File S8.** Mathys et al. prefrontal cortex inhibitory neurons - L1000CDS² reverse query results

**File S9.** Mathys et al. prefrontal cortex inhibitory neurons - differential gene expression results

**File S10.** Green et al. aged prefrontal cortex CUX2-positive excitatory neurons - L1000CDS² mimic query results

**File S11.** Green et al. aged prefrontal cortex CUX2-positive excitatory neurons - L1000CDS² reverse query results

**File S12.** Green et al. aged prefrontal cortex CUX2-positive excitatory neurons - differential gene expression results

**File S13.** Green et al. aged prefrontal cortex CUX2-negative excitatory neurons - L1000CDS² mimic query results

**File S14.** Green et al. aged prefrontal cortex CUX2-negative excitatory neurons - L1000CDS² reverse query results

**File S15.** Green et al. aged prefrontal cortex CUX2-negative excitatory neurons - differential gene expression results

**File S16.** Green et al. aged prefrontal cortex inhibitory neurons - L1000CDS² mimic query results

**File S17.** Green et al. aged prefrontal cortex inhibitory neurons - L1000CDS² reverse query results

**File S18.** Green et al. aged prefrontal cortex inhibitory neurons - differential gene expression results

**File S19.** TSA vs DMSO differential gene expression results

**File S20.** **Conserved TSA-responsive genes across mouse and human neuronal systems.** List of 1,669 genes with 1:1 mouse-human orthology showing significant differential expression (|log₂FC| > 0.58, p < 0.05) in response to TSA treatment in both mouse primary hippocampal neurons and human iPSC-derived cortical neurons, with log₂ fold-changes and statistical values for each species.

**File S21**. **Microglial subtype-specific differential gene expression in fresh tissue (Lee et al. FreshMG cohort).** Differential expression analysis results for 13 microglial subtypes from the Lee et al. FreshMG cohort (543,012 myeloid cells from 137 postmortem brain specimens). Each microglial subtype is represented by two sheets: genes upregulated (|up) and downregulated (|dn) in that subtype relative to all other myeloid cells. Subtypes include: disease-associated microglia (ADAM_GPNMB), inflammatory/adaptive states (Adapt_AIF1, Adapt_CCL3, Adapt_HIF1A, Adapt_HIST, Adapt_HSPA1A, Adapt_IFI44L, Adapt_TMEM163), homeostatic microglia (Homeo_FRMD4A, Homeo_PICALM), perivascular macrophages (PVM_CD163), proliferative microglia (Prolif_MKI67), and expanded microglia (exAM_ERN1). Columns: featurekey (gene symbol), log2Mean (mean expression in target subtype), log2Mean_other (mean expression in all other subtypes), log2FC (log2 fold change), percentage (% of cells expressing in target subtype), percentage_other (% of cells expressing in other subtypes), percentage_fold_change (fold change in expression percentage), auroc (Area Under Receiver Operating Characteristic curve for subtype discrimination), mwu_U (Mann-Whitney U statistic), mwu_pval (Mann-Whitney U test p-value), mwu_qval (Mann-Whitney U test FDR-adjusted q-value).

**File S22.** Microglial subtype-specific differential gene expression in frozen tissue (Lee et al. PsychAD cohort). Differential expression analysis results for 13 microglial subtypes from the Lee et al. PsychAD cohort (289,493 myeloid nuclei from 1,470 donors). Structure and subtypes identical to File S20. This parallel analysis from frozen tissue validates the reproducibility of microglial subtype-specific gene signatures across tissue preparation methods. Column definitions identical to File S20.

**File S23.** **Venn diagram gene intersections: TSA treatment, microglia, and AD neurons (Figure 5D).** Complete gene lists for overlapping regions in **Fig. 5D** Venn diagram showing upregulated genes shared between TSA-treated neurons, microglial subtypes, and AD-associated neurons. Includes DISC1 as the central three-way intersection gene and all pairwise intersection sets.

# List of abbreviations

AD - Alzheimer's disease

ADAM - Disease-associated microglia

AGE-RAGE - Advanced glycation end products-receptor for advanced glycation end products

AIF1 - Allograft inflammatory factor 1

ANK3 - Ankyrin-3

APOE - Apolipoprotein E

APP - Amyloid precursor protein

ASGARD - A Single Cell Guided Pipeline to Aid Repurposing of Drugs

Ast - Astrocytes

ATP1A2 - ATPase Na+/K+ transporting subunit alpha 2

AUROC - Area Under Receiver Operating Characteristic

Aβ - β-amyloid

BACE1 - Beta-secretase 1

cAMP - Cyclic adenosine monophosphate

CCL3 - C-C motif chemokine ligand 3

CDC - Centers for Disease Control and Prevention

CDK - Cyclin-dependent kinase

cGMP - Cyclic guanosine monophosphate

CNS - Central nervous system

COL24A1 - Collagen type XXIV alpha 1 chain

CREB - cAMP response element-binding protein

CRH - Corticotropin releasing hormone

CSF - Cerebrospinal fluid

CUX2 - Cut like homeobox 2

DEG - Differentially expressed gene

DEGAS - Diagnostic Evidence GAuge of Single cells

DHFR - Dihydrofolate reductase

DISC1 - Disrupted in schizophrenia 1

DIV - Days in vitro

DNA - Deoxyribonucleic acid

Dou - Doublets

DPYD - Dihydropyrimidine dehydrogenase

EDIL3 - EGF like repeats and discoidin domains 3

End - Endothelial cells

EPAC - Exchange protein activated by cAMP

ERK - Extracellular signal-regulated kinase

ETV1 - ETS variant transcription factor 1

Ex - Excitatory neurons

FDA - Food and Drug Administration

FDR - False discovery rate

FN1 - Fibronectin 1

FRMD4A - FERM domain containing 4A

GABA - Gamma-aminobutyric acid

GAP43 - Growth associated protein 43

GLUL - Glutamate-ammonia ligase

GO - Gene Ontology

GPNMB - Glycoprotein nmb

GSK3β - Glycogen synthase kinase 3 beta

HDAC - Histone deacetylase

HIF - Hypoxia-inducible factor

HPA - Human Protein Atlas

HPC - High-performance computing

HSPA1A - Heat shock protein family A member 1A

IFI44L - Interferon induced protein 44 like

IGFBP5 - Insulin like growth factor binding protein 5

In - Inhibitory neurons

Inh - Inhibitory neurons

iPSC - Induced pluripotent stem cell

ISOX - HDAC6 inhibitor

JAK-STAT - Janus kinase-signal transducer and activator of transcription

JNK - c-Jun N-terminal kinase

JUN - Jun proto-oncogene

LAMP5 - Lysosomal associated membrane protein family member 5

LINCS - Library of Integrated Network-Based Cellular Signatures

MALAT1 - Metastasis associated lung adenocarcinoma transcript 1

MAPK - Mitogen-activated protein kinase

MBP - Myelin basic protein

Mic - Microglia

MITF - Melanocyte inducing transcription factor

MKI67 - Marker of proliferation Ki-67

MOBP - Myelin associated oligodendrocyte basic protein

MSBB - Mount Sinai Brain Bank

MTRNR2L - MT-RNR2 like

mTOR - Mechanistic target of rapamycin

MTS - Metabolic activity assay

NEAT1 - Nuclear paraspeckle assembly transcript 1

Neu - Neurons

NF-κB - Nuclear factor kappa B

NFT - Neurofibrillary tangles

NIA - National Institute on Aging

NT - Non-treated

Oli - Oligodendrocytes

Opc - Oligodendrocyte precursor cells

P2RY12 - Purinergic receptor P2Y12

PCA - Principal component analysis

PDE - Phosphodiesterase

PI3K-Akt - Phosphoinositide 3-kinase-protein kinase B

PICALM - Phosphatidylinositol binding clathrin assembly protein

PKA - Protein kinase A

PVM - Perivascular macrophages

QKI - Quaking homolog, KH domain RNA binding

Rap1 - Ras-related protein 1

RASD2 - RASD family member 2

RNA - Ribonucleic acid

ROSMAP - Religious Orders Study and Memory and Aging Project

SAHA - Suberoylanilide hydroxamic acid

scRNA-seq - Single-cell RNA sequencing

SLC1A2 - Solute carrier family 1 member 2

SPDYE - Speedy/RINGO cell cycle regulator family member E

SST - Somatostatin

ST - Spatial transcriptomics

STAT - Signal transducer and activator of transcription

STMN2 - Stathmin 2

TMEM163 - Transmembrane protein 163

TMM - Trimmed mean of M-values

TNF - Tumor necrosis factor

TREM2 - Triggering receptor expressed on myeloid cells 2

TSA - Trichostatin-A

TSHZ2 - Teashirt zinc finger homeobox 2

TUBA1A - Tubulin alpha 1a

TUBA1B - Tubulin alpha 1b

UMAP - Uniform Manifold Approximation and Projection

Vasc - Vascular cells

VEGF - Vascular endothelial growth factor

WFS1 - Wolfram syndrome 1

# Author Contributions

M.P. designed the study, performed computational drug repurposing analysis, conducted single-cell RNA sequencing data processing and analysis, performed integrative cross-dataset analysis, analyzed iPSC-derived neuronal transcriptomic data, generated all figures and tables, and wrote the manuscript. N.J.G. performed all wet laboratory experiments including iPSC-derived cortical neuron culture, cell viability assays, synaptic integrity assessments, and RNA extraction for transcriptomic analysis, and wrote the corresponding experimental methods sections. J.L. conducted the comprehensive analysis of microglial subtypes using the Lee et al. [10] dataset for both fresh and frozen tissue samples, performed differential expression analysis across microglial populations, and wrote the methods section for microglial characterization. C.B. performed Gene Ontology enrichment analysis of DEGs from TSA-treated hippocampal neurons and contributed to the interpretation of pathway analysis results presented in Figure 5. C.S. conducted spatial transcriptomics analysis using 10x Genomics Visium datasets, performed TSA gene signature mapping onto brain tissue sections, and contributed to the spatial analysis methods and results presented in the supplementary materials. S.B. provided thorough review of the manuscript writing and conducted statistical review of the analyses. T.I.R. provided chemistry, pharmacology, and biological expertise and knowledge and reviewed and edited the manuscript. P.Z., S.D.M., and K.H. served as advisors providing statistical knowledge and expertise throughout the study. C.A.L.R. served as advisor to N.J.G. and provided wet laboratory expertise for experimental design and validation studies. J.Z. served as advisor and contributed genetics and genomics expertise to the study design and data interpretation. T.S.J. served as primary advisor and provided statistics, genomics, and bioinformatics expertise, supervised the overall project, contributed to study design and data interpretation, and edited the manuscript. All authors reviewed and approved the final manuscript.

# Availability of data and materials

The datasets supporting the conclusions of this article are available in the following repositories:

- The Grubman et al. single-cell RNA sequencing dataset is available in the NCBI Gene Expression Omnibus (GEO) repository, <http://www.ncbi.nlm.nih.gov/geo/query/acc.cgi?acc=GSE138852>.
- The Mathys et al. single-cell RNA-seq dataset is available in the Synapse repository, [http://www.synapse.org/ - !Synapse:syn18485175](http://www.synapse.org/#!Synapse:syn18485175).
- The Green et al. RNA-seq dataset is available in the Synapse repository, <https://www.synapse.org/#!Synapse:syn31512863>.
- The Mount Sanai Brain Bank (MSBB) study is available in the Synapse repository, [http://www.synapse.org/ - !Synapse:syn3159438](http://www.synapse.org/#!Synapse:syn3159438).
- The Lee et al. FreshMG and PsychAD single-cell datasets are available in the Synapse repository, <http://www.synapse.org/#!Synapse:syn52795292>.
- The RNA-seq dataset for TSA-treated hippocampal neurons is available in the NCBI GEO repository, <http://www.ncbi.nlm.nih.gov/geo/query/acc.cgi?acc=GSE189117>.
- The Visium spatial transcriptomics dataset used in this study is available from the 10x Genomics Datasets Portal: <http://www.10xgenomics.com/datasets/aggregate-of-mouse-brain-sections-cytassist-for-ffpe-2-standard>
- LINCS L1000 drug perturbation data were obtained from the NCBI GEO repositories:
  - GSE70138: <http://www.ncbi.nlm.nih.gov/geo/query/acc.cgi?acc=GSE70138>
  - GSE92742: <http://www.ncbi.nlm.nih.gov/geo/query/acc.cgi?acc=GSE92742>
- Human tissue expression data were accessed from the Human Protein Atlas, <http://www.proteinatlas.org>, and the GTEx Portal, <http://www.gtexportal.org>, both of which are publicly available for academic use.
- The transcriptomic data generated from iPSC-derived cortical neurons subjected to TSA and Aβ treatments is available in the NCBI GEO repository under accession number GSE302545, <https://www.ncbi.nlm.nih.gov/geo/query/acc.cgi?acc=GSE302545>
